# Five years of veterinary forensic necropsies in the Netherlands: a retrospective analysis of non-human cases

**DOI:** 10.1007/s00414-026-03840-y

**Published:** 2026-06-03

**Authors:** Monique Verkerk, Thomas P. Shehata

**Affiliations:** 1 Stichting Forensisch Dierenonderzoek, Dorpsstraat 253, Warmenhuizen, 1749 AE The Netherlands; 2 Department of Biomedical Engineering and Physics, Amsterdam UMC, location AMC, University of Amsterdam, Meibergdreef 9, Amsterdam, 1105 AZ The Netherlands; 3 Department of Human Biology, University of Cape Town, Cape Town, WC South Africa; 4 Wildlife Forensic Academy, R27, West Coast, 7345 South Africa; 5 Royal Rotterdam Zoological & Botanical Gardens, Blijdorplaan 8, Rotterdam, 3041 JJ The Netherlands

**Keywords:** Veterinary forensic science, Animal abuse, Necropsy, Neglect and deprivation, Postmortem

## Abstract

In this study a five-year retrospective analysis (2020–2024) of veterinary forensic necropsies in the Netherlands is presented, based on 346 necropsies out of 390 total submissions to Stichting Forensisch Dierenonderzoek (Forensic Animal Research Foundation), involving suspected non-natural deaths with potential forensic relevance. The objective was to characterize animal abuse cases by assessing species involvement, presumptive causes and manners of death based on gross findings, and regional, temporal, and procedural patterns. Forensic necropsies were performed according to standardized internal protocols, and data were analysed using descriptive and inferential statistics. The majority of examined animals were companion species, particularly dogs (41.8%) and cats (28.5%). Presumptively classified non-natural deaths accounted for 66.8% of cases, while natural deaths represented 9.5%, accidents 8.4%, and undetermined cases 15.3%. Among non-natural deaths, blunt force trauma (30.3%) and neglect or deprivation (26.4%) were the most frequent causes, based on gross necropsy findings. Seasonal variation in decomposition stage and regional disparities in submissions were observed. Submission and packaging practices improved modestly over time, yet forensic standards were not consistently met. Legal outcomes were retrieved for 80 cases: most resulted in minor penalties such as fines or community service, with only one case leading to imprisonment exceeding six months. These findings underscore the dominance of violent and neglect-related deaths in Dutch veterinary forensic caseloads and emphasize the need for standardized protocols, interdisciplinary collaboration, and institutional support for veterinary forensic science in the Netherlands.

**Graphical abstract:**

**Figure:**
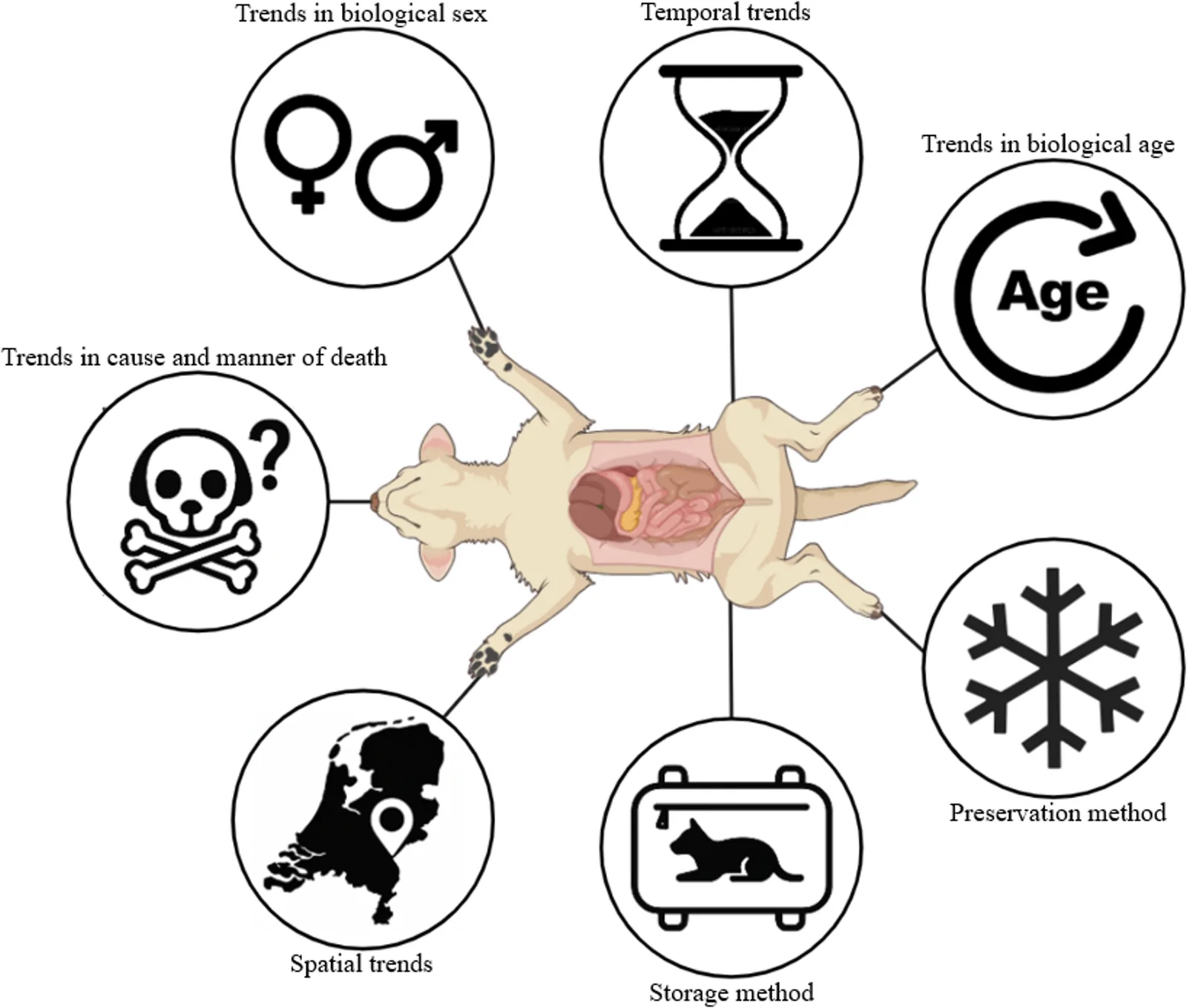

**Supplementary Information:**

The online version contains supplementary material available at 10.1007/s00414-026-03840-y.

## Highlights

This is the first systematic study on veterinary forensic necropsies in the Netherlands.

The majority of cases involved companion animals, particularly dogs and cats.

Blunt force trauma and neglect were the most prevalent presumptive causes of non-natural death.

Presumptive non-natural deaths accounted for two-thirds of all submitted cases.

Regional and seasonal trends were identified in case submission and cause of death.

**Supplementary Information:**

The online version contains supplementary material available at 10.1007/s00414-026-03840-y.

## Introduction

### Interpersonal violence and animal abuse

Interpersonal violence is a significant public health and social concern with severe societal and individual consequences [1]. Over the past two decades, a substantial body of research has demonstrated a consistent association between interpersonal violence and animal abuse [2–7]. Acts of cruelty toward animals are increasingly recognized as indicators of broader patterns of violence, including domestic abuse, child abuse, and other criminal behaviours [8–12].

In the Netherlands, the relationship between interpersonal violence and animal abuse gained attention in 2009, when research revealed that 60% of veterinarians treating companion animals (*N* = 108) had observed signs of abuse in their practice [13]. Follow-up research further demonstrated that domestic violence and animal abuse frequently coexist within households, highlighting the need for early detection and documentation by professionals such as veterinarians, social workers, and law enforcement officers [14]. These findings emphasize the importance of veterinary forensic investigations not only for establishing cause and manner of death, but also for generating evidence that contributes to both legal proceedings and violence-prevention strategies [15, 16].

### Veterinary forensics in the Netherlands

Veterinary forensic pathology applies postmortem investigative methods to determine the cause and manner of death in animals, differentiating between natural and non-natural causes such as trauma, neglect, or poisoning. In the Netherlands, these investigations play a key role in enforcing the *Wet Dieren* (Animal Law) and relevant sections of the Dutch Penal Code, which criminalize intentional or negligent harm to animals (see Supplementary Material 1) [17–21]. Reports generated from necropsies are frequently used in court cases to substantiate claims of abuse and establish forensic evidence that can link suspects to crimes [17]. Forensic findings can support legal proceedings and strengthen the evidentiary basis for prosecution.

Veterinary forensics is a developing field in the Netherlands, and most cases of suspected abuse are investigated through collaboration between police authorities and independent forensic organizations, such as Stichting Forensisch Dierenonderzoek (Forensic Animal Research Foundation). These institutions perform postmortem examinations, assist with evidence interpretation, and provide expert testimony in court when required.

### A retrospective analysis of veterinary forensic cases in the Netherlands

Although veterinary forensic medicine has gained recognition internationally [22–27], systematic analyses of Dutch veterinary forensic casework have not yet been published. To date, there is no comprehensive dataset describing the prevalence, causes, and judicial outcomes of animal abuse cases in the Netherlands. This absence of consolidated national data limits the development of evidence-based prevention strategies, enforcement policies, and professional training programs.

The present study provides the first multi-year retrospective overview of necropsies conducted between January 2020 and December 2024 by Stichting Forensisch Dierenonderzoek (Forensic Animal Research Foundation), the primary institution performing veterinary forensic investigations in the Netherlands. A smaller number of cases are handled by Utrecht University and Wageningen University & Research [17].

The main objective of this study is to characterize the causes and manners of death in animals submitted under suspicion of abuse or other non-natural causes, identify temporal and geographical trends, and biological profiles and evaluate the evidentiary and legal outcomes of these investigations. By analysing patterns in deaths, investigative findings, and case outcomes, this study provides the first structured insight into the scope and nature of veterinary forensic practice in the Netherlands. The findings aim to support law enforcement and judicial authorities, inform policy development, and contribute to the standardization and advancement of veterinary forensic pathology nationally and internationally.

## Materials & methods

### Case selection and inclusion criteria

Between January 2020 and December 2024, a total of 390 cases involving 438 animals were submitted to Stichting Forensisch Dierenonderzoek (Forensic Animal Research Foundation). Of these, 346 animals (79.0%) underwent a complete necropsy, while the remaining cases were assessed through digital forensic analysis, which included the evaluation of photographic or video evidence provided by law enforcement authorities, animal welfare organizations, or members of the public when carcasses were unavailable or animals were still alive.

Inclusion criteria required that cases involve animals suspected of non-natural or abuse-related deaths, with submissions intended for potential legal evaluation. All cases were treated as having forensic potential, meaning they were processed using standardized internal protocols to preserve evidentiary integrity within our facility, including systematic documentation, photography, and sample collection for ancillary testing (e.g., toxicology).

However, the chain of custody prior to submission was not consistently documented by submitting parties, which may affect the legal robustness and interpretability of findings. Necropsies were nevertheless conducted under controlled forensic conditions to support reconstruction of events based on available evidence (e.g., injuries, decomposition stage, or contextual information), in line with established definitions of forensic science [28].

Cases were primarily submitted by law-enforcement authorities, municipal animal-welfare services, and non-governmental organizations, with occasional contributions from private veterinarians or individual citizens. Background information provided at submission was often limited, typically including species, and in some instances breed, sex, geographic location, and the suspected type of abuse. Ownership status (e.g., stray versus owned) was rarely indicated.

**Fig. 1:**
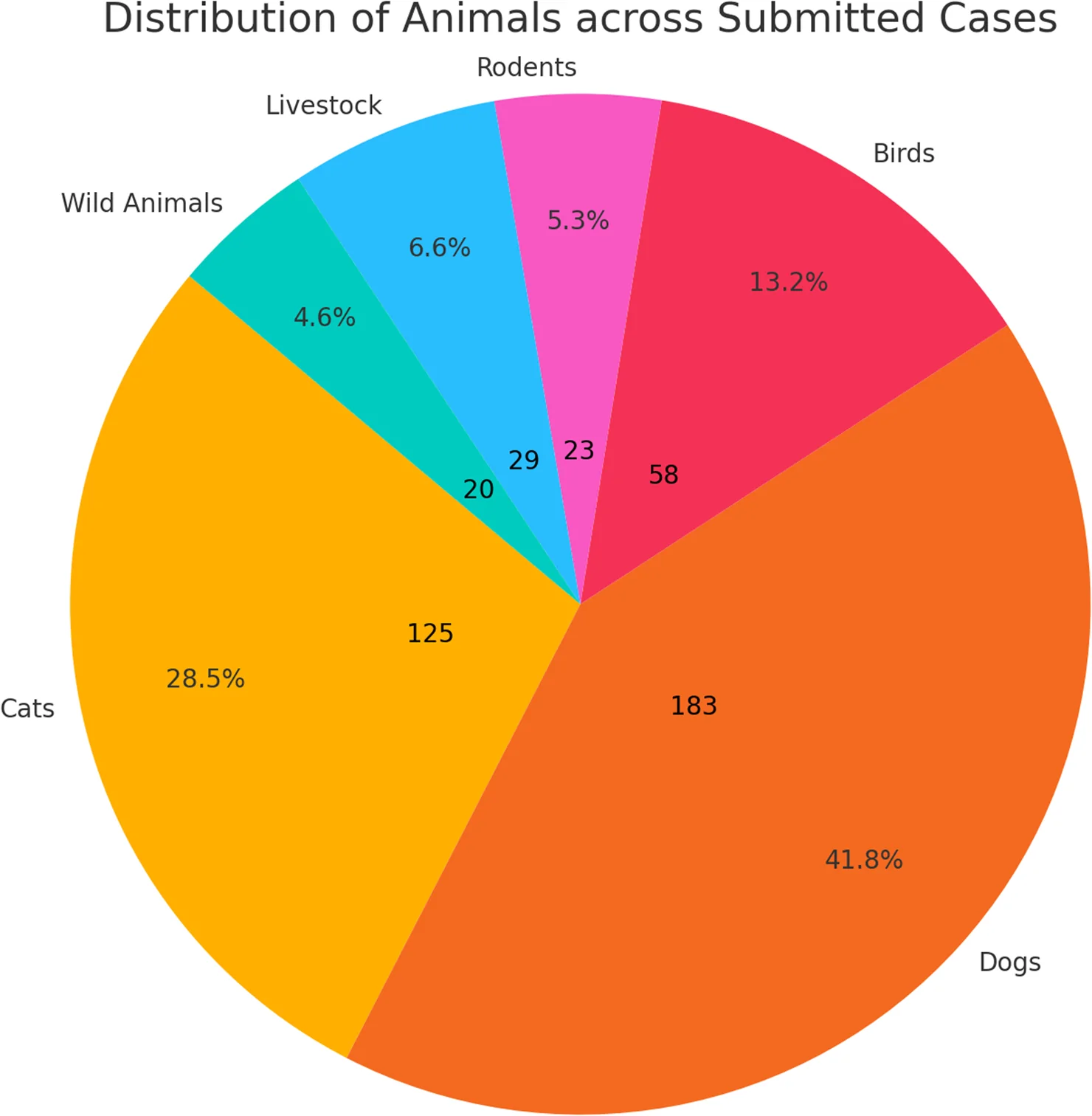
A pie chart of the distribution of animals across submitted cases (*N* = 438), categorized in the categories dogs (orange), cats (yellow), birds (red), livestock (blue), rodents (pink) and wild animals (green)

Dogs constituted the majority of submitted species (*n* = 183/438, 41.8%), followed by cats (*n* = 125/438, 28.5%) and birds (*n* = 58/438, 13.2%) across both necropsy and digital forensic cases (Fig. 1).

Full-body radiography was performed in cases with suspected firearm involvement, and imaging findings were incorporated into the forensic reports. All necropsies and classifications were performed by a single qualified veterinary forensic scientist using standardized internal protocols. No second-examiner review or formal assessment of interobserver variability was conducted, which may affect reproducibility.

### Necropsy procedure

A comprehensive postmortem examination was performed on each animal following a standardized forensic protocol. When possible, the breed was confirmed, and animals were scanned for subcutaneous microchips or examined for external identification such as ear tags. If sex and age were unknown, they were estimated during the examination.

Age estimation was based on dentition, including tooth eruption and wear, classifying animals into three age groups: juvenile (less than ten months), adult (eleven months to ten years), and senior (older than ten years). For companion animals, it was also assessed whether they were neutered.

The decomposition stage was assessed, when requested by the submitting agency, based on gross morphological characteristics using an internally defined operational scheme (Supplementary Material 2).

The external examination included systematic documentation of coat condition, body weight, dental and nutritional status, nail integrity, oral cavity, eyes and ears, neck mobility, torso condition, anogenital region, and any observable injuries or distinguishing features. Following the external examination, a systematic internal examination was conducted to assess organ condition, structural integrity, and potential pathological changes. The abdominal cavity was assessed for abnormalities, including the presence of blood, fluid, haemorrhages, or contusions. The intestinal tract, bladder, kidneys, adrenal glands, spleen, stomach, liver, and diaphragm were examined for structural or macroscopic pathological changes, as well as content where applicable. The thoracic cavity was inspected for blood, fluid, haemorrhages, or contusions. The heart was evaluated for macroscopic abnormalities. The lungs, oesophagus, and neck muscles were examined for any deviations from normal anatomy. Special attention was given to the trachea for the presence of fluid or foam. The skull and brain were also examined for abnormalities. Any additional findings were documented.

Photographic documentation was performed throughout the examination, and the packaging method at submission was recorded. Tissue and content samples were collected and preserved (e.g., formalin-fixed or frozen) for potential toxicological or other ancillary analyses; however, such analyses were only performed upon request by judicial authorities.

### Determination of cause and manner of death

Cause and manner of death were determined based exclusively on gross necropsy findings. Histopathological examination and ancillary testing (e.g., toxicology, microbiology) were not performed within the authors’ institution. When additional analyses were indicated (e.g., for suspected poisoning or subtle lesions), tissues, organs, or contents (e.g., liver, stomach contents, blood) were collected and preserved (e.g., formalin-fixed or frozen) for retrieval by the submitting agency, which was responsible for external laboratory submission. Results from such analyses were not available to the authors and were therefore not incorporated into the dataset. Accordingly, all determinations should be interpreted as operational and presumptive classifications rather than definitive forensic diagnoses. Gross examination alone may not detect conditions such as intoxication, subtle trauma, asphyxia, infectious processes, or postmortem artifacts in all cases, and may therefore introduce a risk of misclassification.

The cause of death was defined as the primary pathological process considered to have led to death, based on macroscopic findings. The manner of death was categorized as natural, non-natural, accidental, or undetermined using a forensic veterinary framework. Non-natural deaths were further subdivided into the following categories: blunt force trauma, sharp force trauma, firearm injuries, asphyxia, bite injuries, fire/explosion injuries, suspected poisoning, and neglect and deprivation. Neglect and deprivation - including withholding of food, water, shelter, or veterinary care - were classified as non-natural deaths in accordance with established forensic veterinary frameworks [22, 29, 30].

### Data analysis

All quantitative analyses were performed using descriptive and inferential statistics with a significance threshold of α = 0.05. Frequencies, proportions, and cross-tabulations were calculated for species, presumptive manner and cause of death, decomposition stage, and submitting agency.

For variables with missing or undetermined values, a complete-case approach was applied: cases were excluded only from analyses involving the variable for which data were absent, and retained in all other analyses. Missing data are reported as separate categories in descriptive summaries and their reasons are described in the relevant Results & Discussion sections.

To explore spatial and temporal trends, one-sample t-tests were conducted for each province, comparing the number of cases to the national mean. Logistic regression models were applied to assess year-to-year trends in cause of death categories, adjusted by Bonferroni correction. Chi-square tests and ANOVAs were used to explore associations between categorical variables (species, cause of death, decomposition stage, and submitting agency).

Given the absence of reliable population-based denominators (e.g., companion animal populations), geographic analyses reflect patterns in submitted cases rather than incidence rates.

Results are presented using bar charts, line graphs, pie charts, heat maps, and stacked area plots to visualize distributions across time, species, and geographic regions.

## Results & discussion

### Number, distribution, and temporal trends of cases

Understanding the geographic and temporal distribution of forensic necropsy cases is essential for identifying regional submission patterns, evaluating trends over time, and informing targeted interventions. Differences in case submissions between provinces and municipalities may reflect regional variation in awareness, enforcement activity, and access to forensic services, rather than true differences in the incidence of animal abuse.

A total of 390 cases were submitted for forensic analysis over five years, of which 44 involved digital evidence. Necropsies were performed on 346 animals; hereafter, case number, submitted animal, and necropsy refer exclusively to these 346 necropsy cases. All cases represent submission-based forensic investigations rather than population-normalized incidence data.

#### Number and distribution of cases

**Fig. 2:**
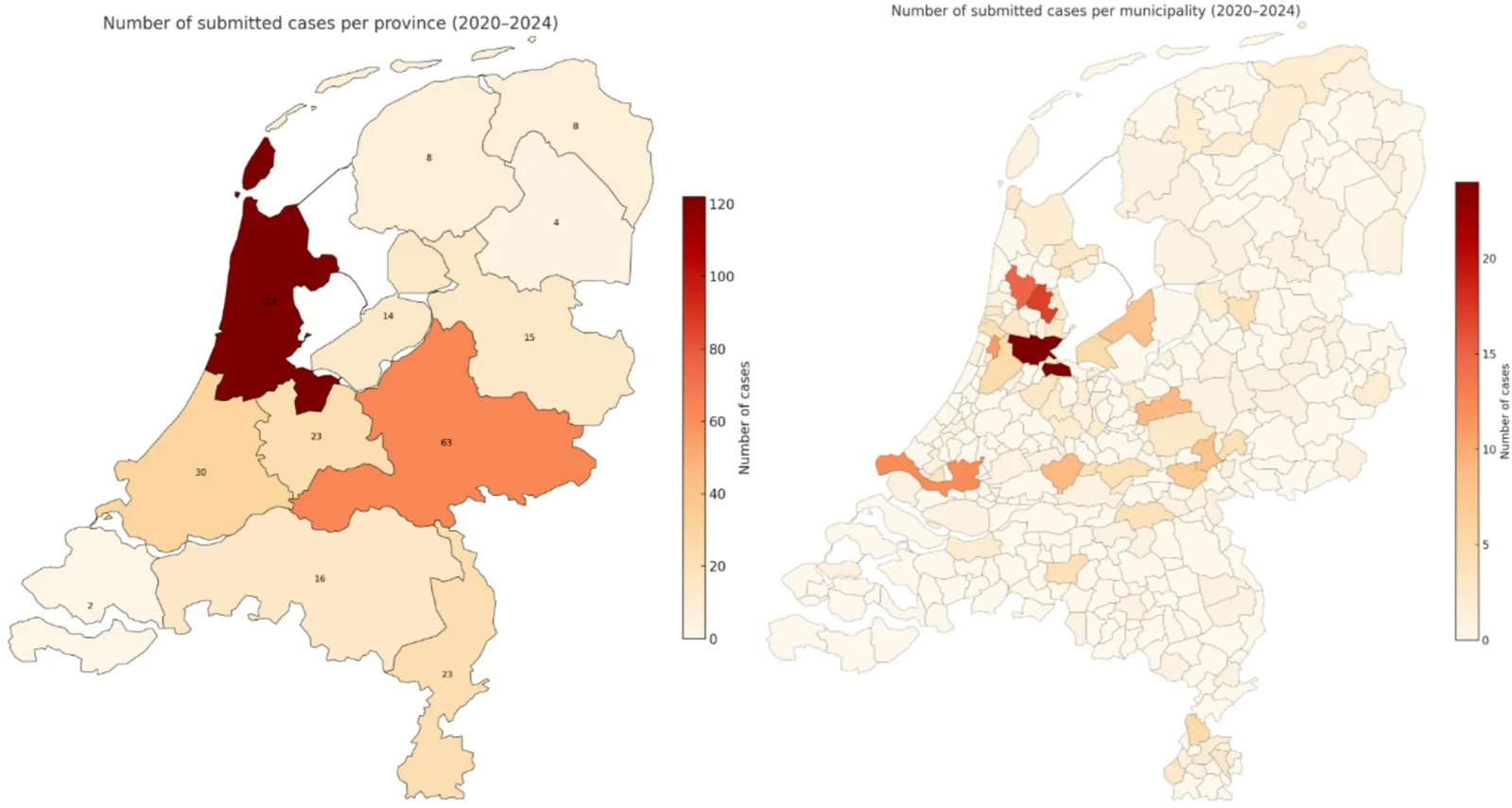
**A** and **B**. The number of cases per province (**A**: left) and municipality (**B**: right) submitted between January 2020 and December 2024 (*N* = 328, 18 unknown). The provincial and municipal boundaries used for the maps are based on the 2025 administrative division of the Netherlands

Submission numbers (*N* = 346) varied by province, with Noord-Holland (*n* = 122) and Gelderland (*n* = 63) reporting the most, and Zeeland the fewest (*n* = 2); province was unknown in 18 necropsies, for example in cases submitted by national-level police units rather than regional divisions (Fig. 2A). Statistical comparisons using one-sample t-tests indicated significantly higher submission numbers in Noord-Holland (t(10) = -20.67, *p* < 0.001) and Gelderland (t(10) = -3.91, *p* = 0.003), while other provinces did not differ significantly from the mean of the remaining regions.

These differences should be interpreted as submission patterns rather than incidence of animal abuse. They may reflect regional variation in reporting behaviour, awareness, enforcement intensity, availability of forensic services, and underlying human or companion animal population density. Normalization by relevant denominators (e.g., human or pet population per province) was not performed in this study. Future studies integrating demographic and epidemiological data could clarify the drivers of these geographic patterns in submissions.

At municipal level, Amsterdam submitted the most cases (6.9%), followed by Purmerend (5.3%), Alkmaar (4.7%), Rotterdam (3.8%), and Haarlem (3.5%) (Fig. 2B); municipality was unknown for 28 necropsies. One-sample t-tests showed significantly higher submissions from Amsterdam (24 necropsies, t = -88.12, *p* < 0.001), Purmerend (17, t = -52.56, *p* < 0.001), Alkmaar (15, t = -44.28, *p* < 0.001), and Rotterdam (12, t = -32.74, *p* < 0.001) compared to other municipalities.

Large cities’ prominence may reflect population size and human–animal interaction density, while Purmerend’s high submissions may be attributable to its historical proximity to the forensic foundation, increasing awareness and access. Most municipalities had few cases, producing a skewed distribution dominated by larger cities, likely influenced by population, local animal welfare networks, and reporting protocols. Spatial cluster analysis could clarify localized hotspots.

Overall, 27% of submissions originated from villages and 73% from urban areas, suggesting differences in detection and reporting rather than incidence. Normalized population-based analyses would be required to confirm any true spatial differences.

#### Temporal trends in number of submitted cases

**Fig. 3:**
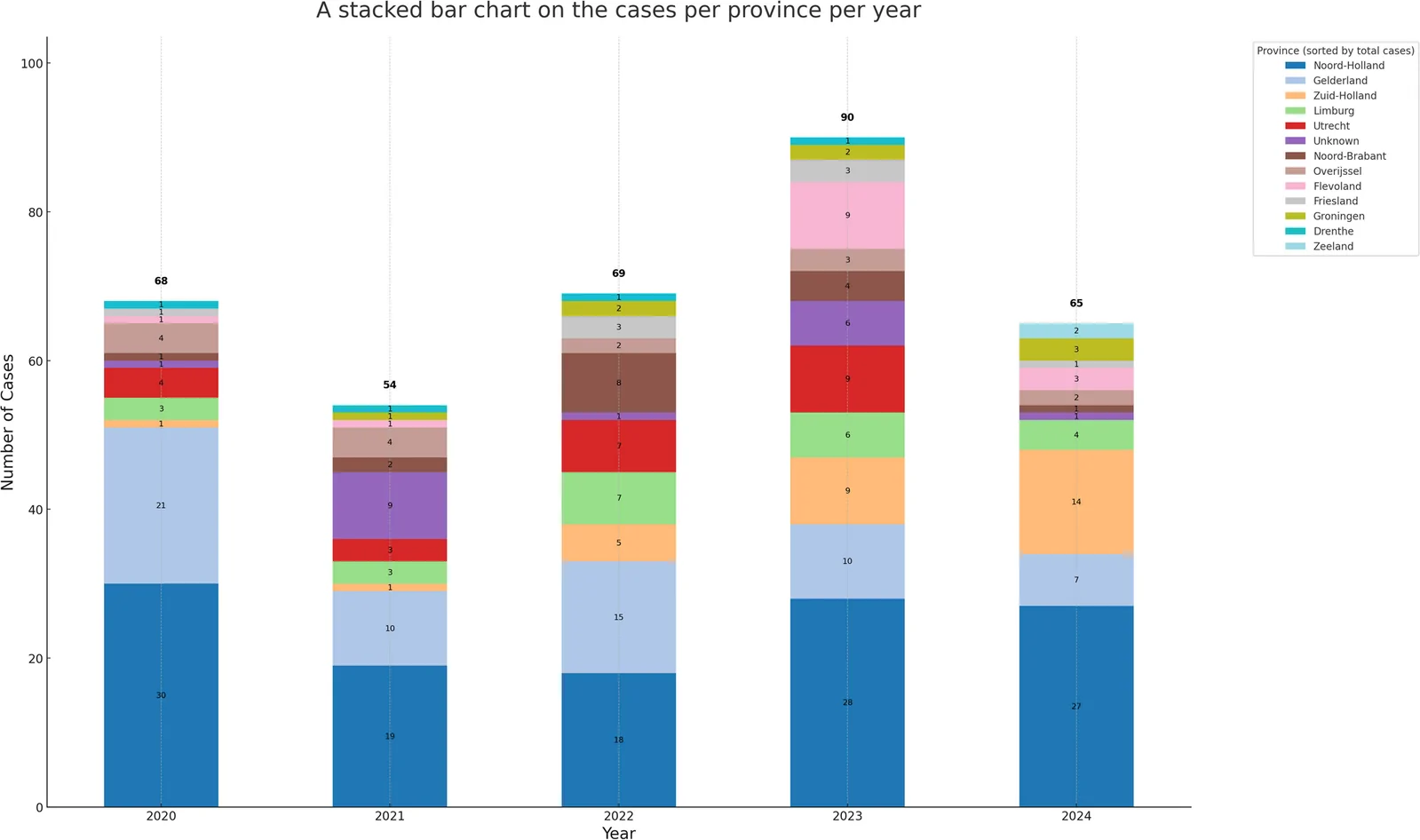
The number of cases per province per year and the total number of necropsies per year (*N* = 346). The total number of cases per year is annotated above each bar, with each coloured segment representing the contribution of a specific province

Figure 3 shows annual submissions per province (2020–2024) (*N* = 346 cases). Total submissions fluctuated over time, peaking in 2023 (*n* = 90) and declining slightly in 2024 (*n* = 65/346), with the lowest count in 2021 (*n* = 54). Noord-Holland consistently had the highest submissions, particularly in 2020 (*n* = 30) and 2024 (*n* = 27), followed by Gelderland. Zuid-Holland showed a rising trend, peaking in 2024 (*n* = 14), possibly attributable to a new designated public prosecutor increasing formal submissions. Zeeland, Drenthe, and Groningen consistently contributed low numbers.

Temporal variation may reflect regional differences in reporting, enforcement, forensic awareness, and external societal factors rather than true changes in incidence. The 2021 decline may be linked to COVID-19 lockdowns, which may have reduced detection and reporting opportunities, which was also seen in cases of child abuse [31, 32]. The increase in 2022–2023 may be consistent with a surge in pet ownership during the pandemic [33], followed by abandonment, neglect, and abuse as owners struggled with behavioural or financial challenges [33]. Similar international patterns were reported in the UK and elsewhere [34, 35]. However, these associations are speculative within the limitations of retrospective submission data.

The 2024 decrease may relate to local police restructuring (as informal reports indicated internal restructuring within several Dutch law enforcement units during that year), normalization post-pandemic, competing priorities (e.g., cybercrime or organized crime), or seasonal effects. Future studies combining administrative data and submitting agency interviews could clarify drivers of annual submission variation.

### Submission, chain of evidence, decomposition, and storage

The origin of forensic submissions and the integrity of the chain of evidence are critical for the admissibility and reliability of forensic results in legal contexts. Identifying which parties most frequently submit cases (e.g., police, veterinarians, animal ambulances) can reveal strengths and weaknesses in the reporting network. Additionally, the condition and handling of animal remains on arrival impact the quality of the examination and interpretation.

#### Submitting agencies and operational timelines

Most cases were submitted by police and governmental agencies (*n* = 260, 74.9% of *N* = 346 cases), followed by private individuals (*n* = 32, 9.2%) and animal ambulances or welfare organizations (*n* = 12, 3.5%). Veterinarians submitted very few cases (*n* = 1, 0.3%), which may reflect procedural reporting pathways, where veterinary suspicion is typically escalated to law enforcement, as well as professional hesitancy possibly attributable to potential reputational or financial risks (Supplementary Material 3A). Lack of clarity on legal responsibilities and insufficient training in identifying and documenting non-accidental injuries may also contribute to systemic underreporting [6, 7, 36]. Addressing this requires targeted training, legal guidance, and institutional support. In 42 cases, the submitting agency could not be definitively determined due to incomplete documentation or ambiguity regarding the primary submitting party when multiple agencies were involved. Some cases involved multiple submitting parties.

Cases submitted by police and governmental agencies (mean 6.2 days, σ = 8.1) took significantly longer to execute than those from non-governmental submitters, including private individuals and animal welfare organizations (mean 3.8 days, σ = 4.3; t(df) = 2.54, *p* = 0.014). Since all necropsies were processed under standardized internal protocols, this difference may reflect external logistical factors such as transportation delays or administrative authorization procedures rather than differences in forensic processing.

#### The decomposition stage of the submitted animals

Submitted cases (*N* = 346 cases) showed considerable variation in decomposition. Most animals were in early/beginning decomposition (*n* = 78, 22.5%) or advanced decomposition (*n* = 69, 19.9%), with fewer animals exhibiting severe/partial skeletonization (*n* = 46/346, 13.3%), moderate decomposition (*n* = 28, 8.1%), mummified/dried remains (*n* = 4, 1.2%), skeletal remains/very advanced decomposition (*n* = 1, 0.3%). Out of 346 animals, 41 (11.8%) showed no signs of decomposition, whereas the decomposition stage of 79 animals (22.8%) could not be determined due to the investigative context or because assessment was not feasible given the limited material available (e.g., submission of only partial remains) (Supplementary Material 3B). These findings indicate that many animals were submitted after significant postmortem interval, which may affect lesion detectability and interpretation.

An ANOVA showed no significant differences in execution time across decomposition stages (F(6, n) = 1.68, *p* = 0.125), indicating that, within the workflow, carcasses in different stages of decomposition are not associated with systematically different processing times. Furthermore, a chi-squared test showed no association between submitting agency and decomposition stage (χ²(12) = 5.01, *p* = 0.958). This suggests that police, private parties, and animal welfare organizations submitted carcasses in similar states of decomposition. Together, these findings support the notion that neither the type of submitting agency nor the condition of the carcass at arrival alone explains variation in processing time. Instead, observed differences in turnaround time (e.g., the longer durations observed for police submissions) are likely driven by logistical factors, such as delays in the actual delivery of carcasses or the time to get a submission approved by the public prosecutor.

Spearman’s correlation showed a weak but significant positive association between decomposition stage and both month (ρ = 0.17, *p* = 0.006) and season (ρ = 0.18, *p* = 0.004), with warmer months linked to more advanced decomposition. Categorizing decomposition as low (fresh/early), medium (moderate/advanced), and high (skeletal/mummified) revealed low-stage cases mostly in winter/early spring, and high-stage cases peaking in summer and autumn. Autumn peaks may reflect delayed discovery rather than recent deaths. The observed pattern underscores the forensic value of prompt scene response and refrigerated storage, especially during high-temperature months, to mitigate decomposition and preserve critical anatomical and trace evidence.

#### Packaging method & Evidence submission

**Fig. 4:**
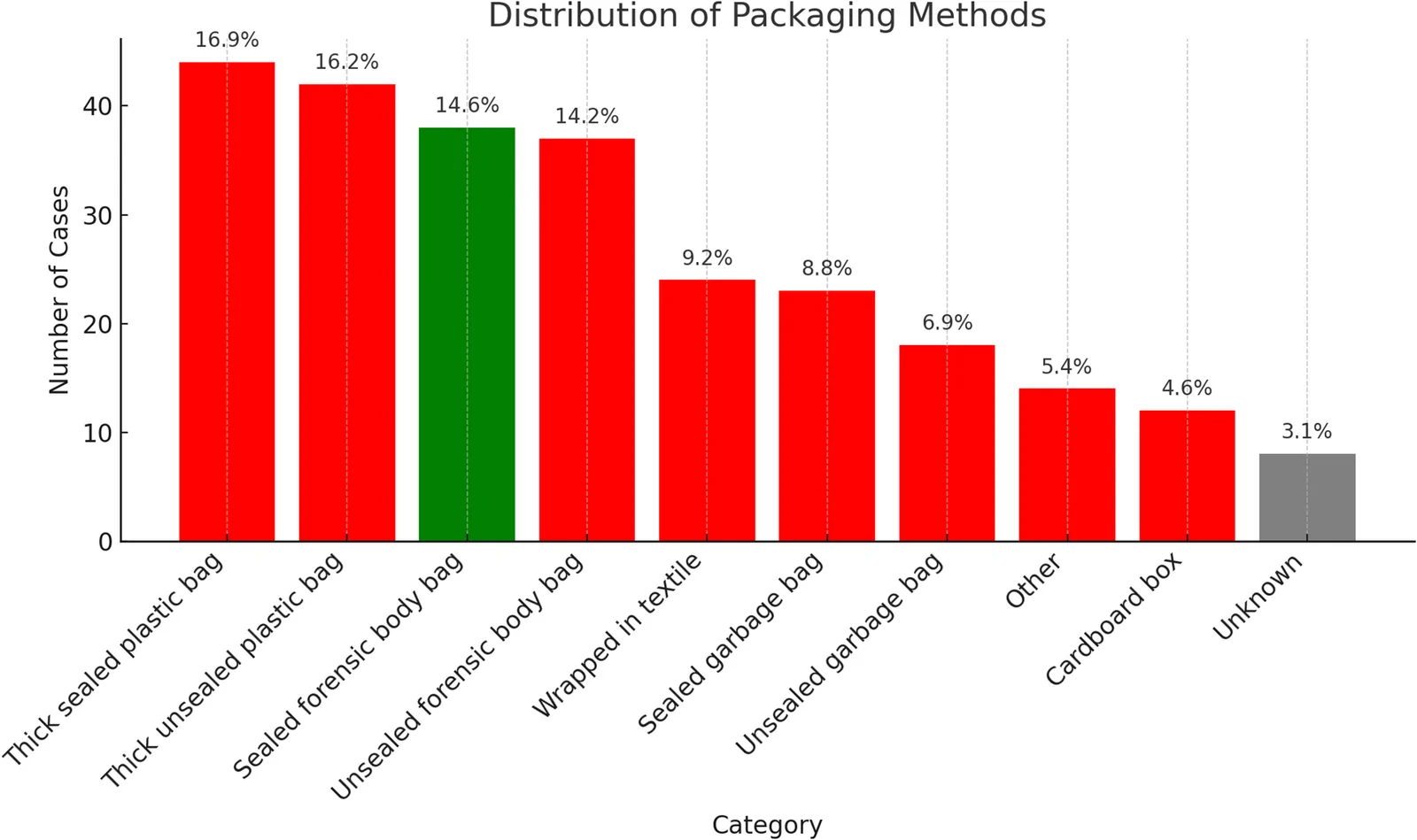
Distribution of packaging methods used by the police and governmental agencies for the submission of animal remains in forensic cases (*N* = 260). In green the proper packaging method, a sealed forensic body bag, in red the non-compliant packaging methods, and in grey the cases when packaging method was unknown. The percentages are noted above the bars

**Fig. 5:**
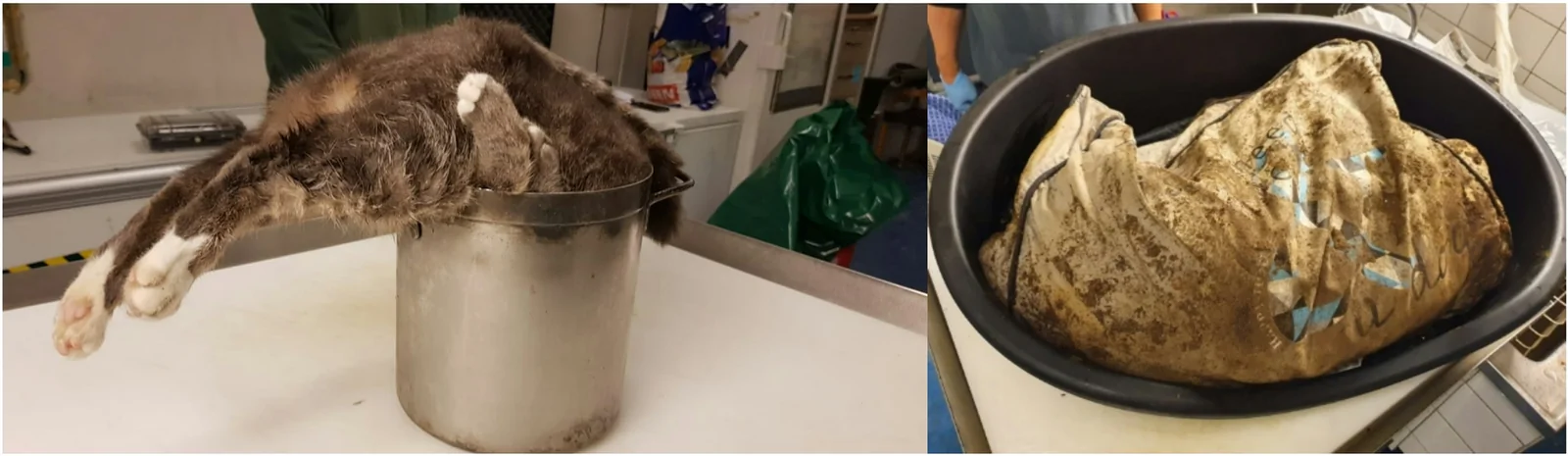
Examples of non-compliant packaging: on the left side a cat submitted in a metal pot and on the right side a dog submitted in a dog bed

Packaging practices varied widely across cases submitted by police and governmental agencies (*N* = 260). Only 14.6% of cases (*n* = 38) were submitted in sealed forensic body bags, while 14.2% used unsealed body bags (*n* = 37), and the majority (82.3%, *n* = 214) were submitted using packaging that did not meet standardized forensic containment criteria. In 3.1% of cases, the packaging was absent/unrecorded (e.g., when necropsies were conducted on-site and no transport packaging was involved) (Fig. 4). The category “Other” included non-standard containers such as metal pots, dog beds, paper bags, and plastic containers (Fig. 5).

To ensure consistency in classification, packaging methods were categorized as *non-compliant* when they did not meet standardized forensic handling criteria for animal remains, as defined in the police forensic evidence handling framework (Supplementary Material 4). This operational classification was applied consistently across all temporal and spatial analyses in this study.

The observed variability in packaging suggests inconsistencies in the handling of forensic evidence and a need for clearer operational guidelines within police and governmental agencies. From a forensic perspective, differences in packaging may influence preservation of biological and trace evidence during transport and storage. However, the present study did not assess the direct impact of packaging method on downstream analytical outcomes.

The observed variability may reflect operational heterogeneity in evidence handling procedures. This may include differences in training and familiarity with forensic handling protocols in animal cases, variability in case workflows, and differences in how animal-related cases are integrated within broader investigative processes.

In addition, several cases included materials such as lodged weapons, ropes, human hairs, or other trace evidence that were not consistently documented or systematically analysed by forensic law enforcement for further forensic analysis. This may have limited the availability of corroborative evidence in those cases.

While law enforcement presence during forensic necropsy is not legally required in the Netherlands for either human or veterinary cases, it is commonly applied in human forensic examinations. In the veterinary cases included in this study, police presence during necropsy was infrequent. From an operational perspective, investigator presence at the examination stage may influence opportunities for immediate case clarification and structured transfer of physical evidence.

#### Temporal and spatial trends in packaging method

**Fig. 6:**
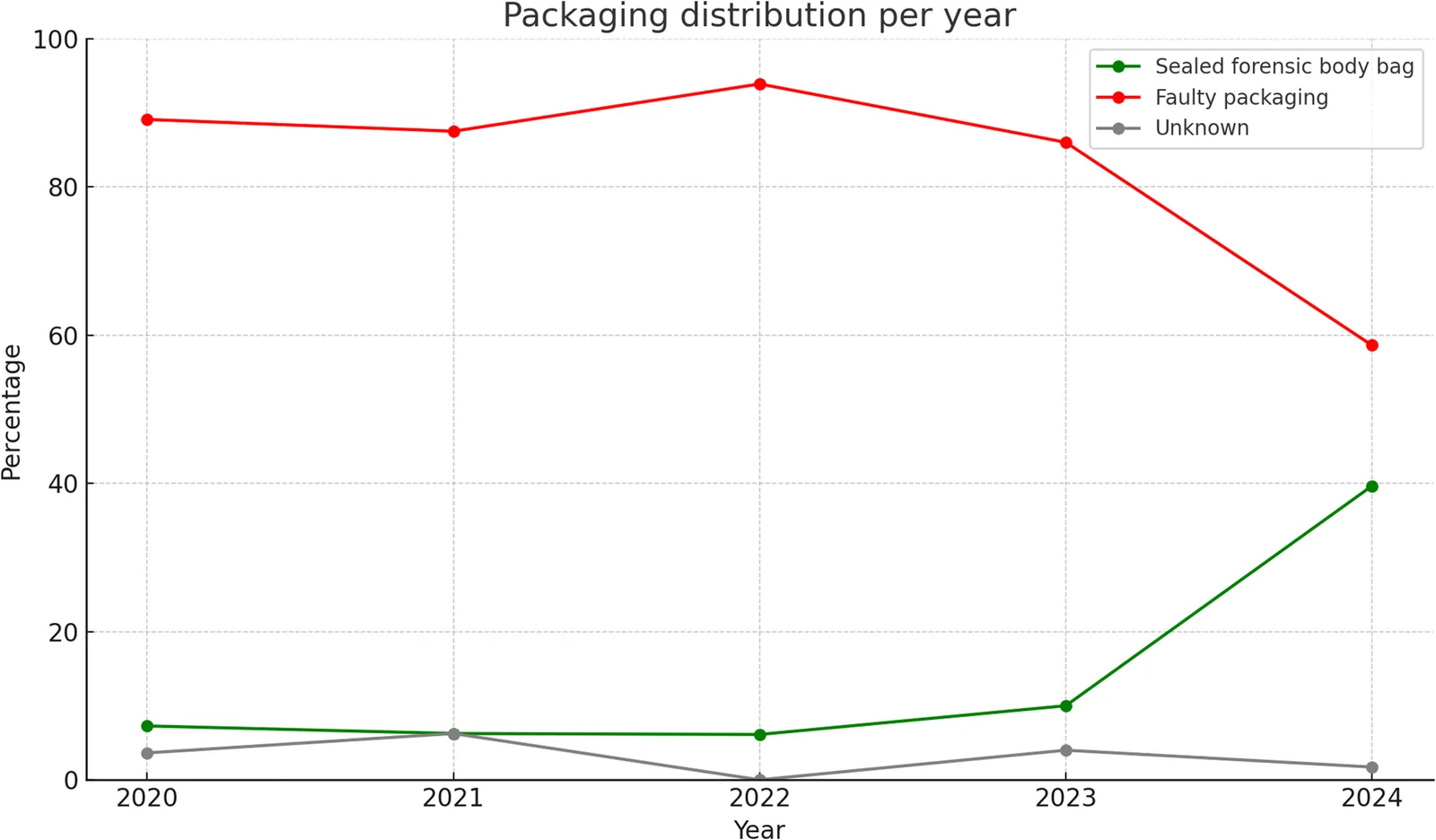
Annual distribution of packaging quality in police-submitted forensic cases from 2020 to 2024 (*N* = 260), showing trends in the use of sealed forensic body bags (green), faulty packaging (red), and unknown packaging methods (grey)

Between 2020 and 2024, non-compliant packaging, was predominant in police-submitted cases (*N* = 260 cases), peaking at 93.5% in 2022, but decreasing to 58.5% in 2024 (Fig. 6). The use of sealed forensic body bags remained limited until 2024, when it increased to 40%, while unknown packaging remained below 6% throughout the study period. These trends suggest gradual improvement, which may be attributable to increased awareness, updated protocols, or training.

Regional variation in packaging methods was observed when restricting analysis to provinces with more than ten submissions per region. Within this subset, Zuid-Holland (28.9%) and Limburg (23.6%) showed the highest proportions of compliant packaging, followed by Utrecht (16.7%). Lower proportions were observed in Noord-Holland (12.7%), Gelderland (11.3%), and Noord-Brabant (4.2%). High case volume did not guarantee proper procedures. Overall, these regional differences may reflect variability in the local application of forensic handling practices. The differences are unlikely to reflect resource availability alone, but may also stem from variations in local leadership, training frequency, inter-agency collaboration, and organizational culture.

#### Storage conditions

**Fig. 7:**
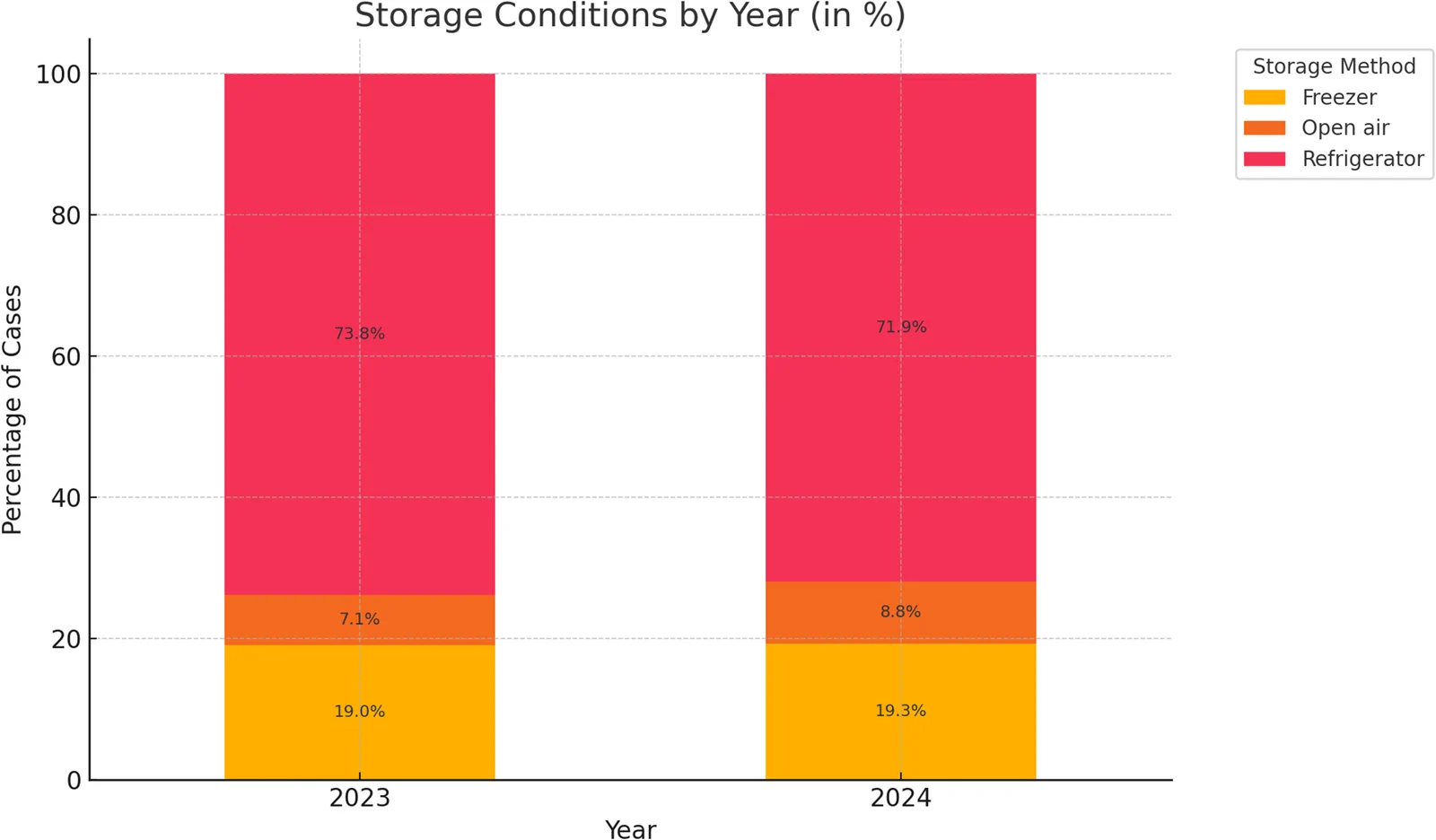
Distribution of storage methods for submitted forensic cases (*N* = 99). The bar chart (right) presents storage conditions by year (2023–2024), expressed as percentages

The majority of forensic cases submitted by the police and governmental agencies, of which storage conditions were known (*N* = 99 cases, mostly in 2023 and 2024), were stored under refrigerated conditions (*n* = 72, 72.7%). Freezer storage accounted for 19.2% (*n* = 19) of cases, while 8.1% were kept in open air prior to submission (*n* = 8). The bar chart (Fig. 7) presents the distribution of storage conditions across 2023 and 2024. Refrigeration remained the predominant storage method in both years, although a slight decrease was observed from 73.8% in 2023 to 71.9% in 2024. Use of freezer storage and open air remained relatively stable, with minor increases in 2024 (freezer: from 7.1% to 8.8%; open air: from 19.0% to 19.3%).

While refrigeration is preferred, a notable proportion of animals were stored under suboptimal conditions, potentially compromising tissue preservation, decomposition assessment, and cause-of-death determination. A subset of cases stored under freezing conditions may be explained by workflows in which animals are initially received by third-party facilities, such as crematoria, prior to forensic screening. In such pathways, storage decisions may be made before any formal forensic assessment of the case, which may result in freezing of cases that are later submitted for forensic examination. This interpretation is based on case context and cannot be systematically verified within the available dataset.

In 2023–2024, regional differences persisted in police storage of animals prior to forensic analysis. Among provinces with ≥ 10 cases, Noord-Holland had the highest refrigeration rate at 85.3%, followed by Zuid-Holland (78.9%) and Gelderland (69.2%). Despite relatively high compliance, a notable portion of cases in all provinces were not stored under ideal forensic conditions. These gaps may reflect differences in police facilities, equipment availability, and internal workflows.

### Cause and manner of death

The forensic determination of cause and manner of death is central to understanding the types of violence inflicted upon animals and guiding criminal prosecution. Patterns in trauma or pathology can reveal whether intentional harm, neglect, or natural causes are more prevalent in the forensic caseload.

The findings presented in the following sections should be interpreted with caution. As detailed above, the chain of custody was not always fully maintained or documented by submitting agencies prior to arrival at our facility, and packaging/storage practices were often suboptimal. These factors may compromise the evidentiary integrity of some traces (e.g., injuries or decomposition evidence), potentially affecting the accuracy or legal defensibility of determinations. While our internal protocols ensured standardized examination and reconstruction of scenarios from available traces, incomplete pre-submission handling introduces uncertainty, emphasizing that conclusions are probabilistic and reliant on expert interpretation rather than absolute proofs. Furthermore, the results presented in this section are based exclusively on gross necropsy findings, without histopathology or ancillary testing, and should therefore be interpreted with caution. These determinations represent presumptive rather than confirmed causes or manners of death, as gross observations alone may not detect subtle pathologies such as intoxication, asphyxia, infectious processes, or postmortem artifacts, potentially leading to misclassification. The absence of follow-up validation (e.g., diagnostic accuracy assessments or interobserver consistency) further limits the forensic reproducibility of these findings. In addition, all necropsies and classifications were performed by a single examiner. The absence of independent second review or interobserver agreement testing represents a limitation that may affect reproducibility and generalizability and could introduce systematic classification bias.

#### Manner of death

The majority of cases (*N* = 346) were classified as presumptive non-natural deaths based on gross necropsy findings only (*n* = 231, 66.8%), including abuse, neglect, or deliberate harm. A smaller proportion of cases were classified as undeterminable (*n* = 53, 15.3%), indicating that the manner of death could not be conclusively established based on the available evidence. Natural deaths accounted for 33 cases (9.5%), and accidents for 29 (8.4%), almost all involving blunt force trauma from traffic incidents (*n* = 28/29, 96.6%), with one case possibly attributable to fire/explosion (Supplementary Material 3C). These results presumptively indicate that forensic submissions predominantly involve suspected unnatural deaths, emphasizing their importance in abuse and neglect investigations.

#### Temporal trends in manner of death

Throughout the five-year period, presumptive non-natural deaths remained the dominant classification. In 2020 and 2021, ~ 70% of cases were classified as non-natural, declining to just under 59% in 2023, coinciding with more undeterminable cases. In 2024, the proportion of non-natural cases rose sharply to nearly 79%, which may reflect improved triage, submission quality, or forensic processing. These temporal variations may reflect changes in case submission patterns, completeness of available evidence, and decomposition state at the time of examination. Given the retrospective design and lack of confirmatory testing, these trends should be interpreted as descriptive rather than indicative of changes in underlying occurrence.

#### Seasonality in manner of death

**Fig. 8:**
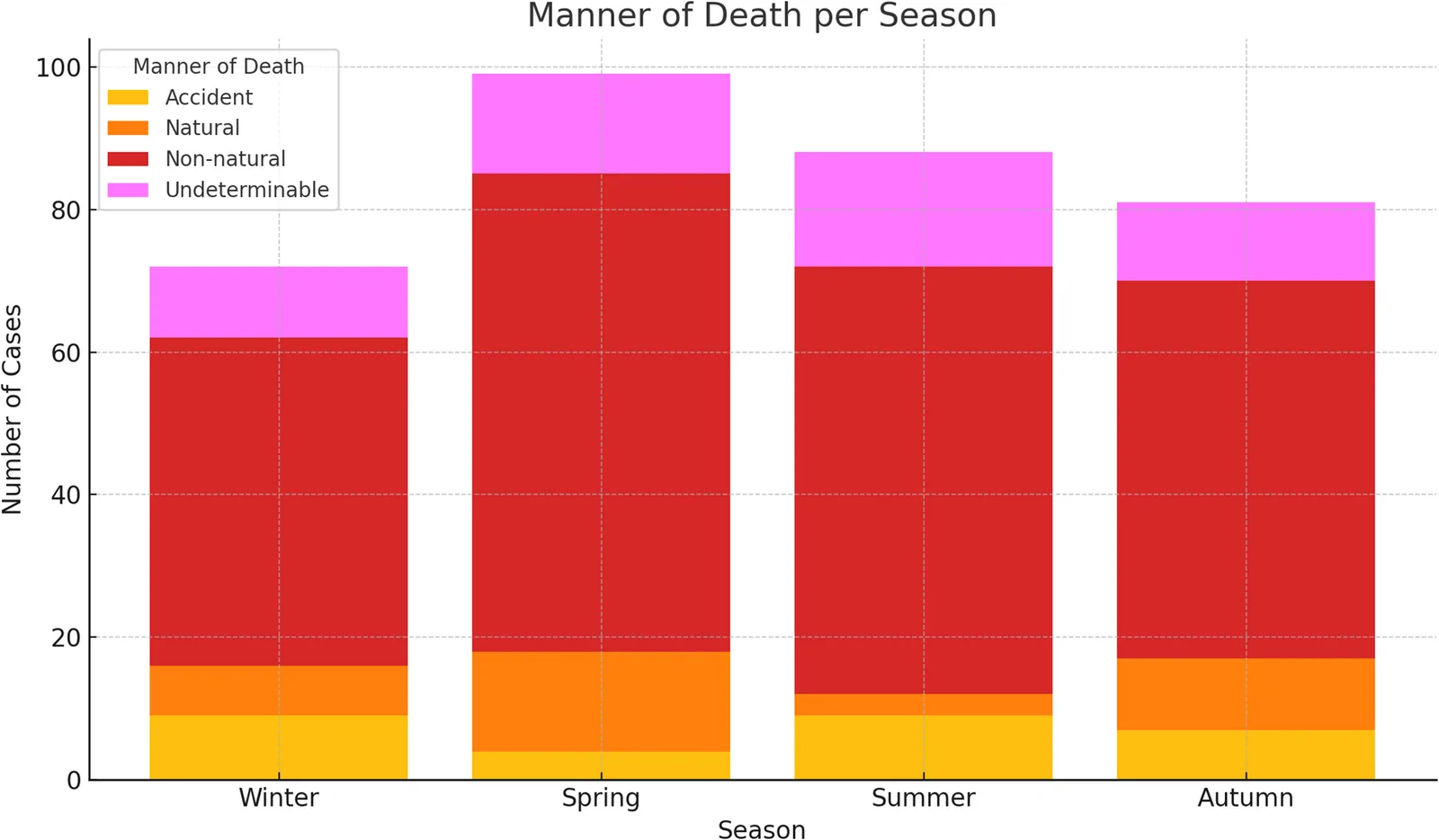
Seasonal distribution of forensic animal cases by manner of death (*N* = 337). Each stacked bar shows the number of cases per season classified as “accident,” “natural,” “non-natural,” or “undeterminable”

A total of 337 cases with known death dates were analysed by season: spring (*n* = 98), summer (*n* = 86), autumn (*n* = 81), and winter (*n* = 72) (Fig. 8). A chi-square test showed no significant seasonal deviation (χ²(3) = 4.50, *p* = 0.21), suggesting variations may be random. Non-natural deaths dominated in all seasons, with the highest in spring (*n* = 67/98), which may be attributable to increased outdoor activity, greater detection of remains, or heightened sensitivity and reporting behaviour as the weather improves. Accidental deaths peaked in winter and autumn (*n* = 16/153). Case reviews suggest that most winter and autumn accidents involved traffic collisions, particularly affecting free-roaming cats. Shorter daylight hours, poor visibility, and harsher environmental conditions likely increase the risk of animals being struck by vehicles during the colder months. However, no statistical seasonality was found, similarly to previous research in Japan [37]. Natural and undeterminable deaths remained relatively stable. These findings suggest that while the overall forensic caseload is relatively stable year-round, certain patterns, such as a spring peak in presumptive non-natural deaths, may point to seasonally modulated risk factors.

#### Relation stage of decomposition and determination of manner of death

Cases with advanced decomposition were significantly more likely to be classified as “undeterminable” for manner of death. A chi-square test confirmed a strong association between decomposition stage and determinability (χ²(6) = 22.79, *p* = 0.0009), indicating that increasing postmortem degradation is associated with reduced ability to assign manner of death based on gross necropsy findings.

#### Non-natural cause of death

Among the cases presumptively classified as non-natural deaths (*N* = 231), blunt force trauma was, similarly to studies in other countries [22, 38], the most common based on gross necropsy findings (*n* = 70, 30.3%), likely because it can be inflicted impulsively with everyday objects and often mimics accidental injury, complicating detection and investigation [25]. This was followed by neglect and deprivation (*n* = 61, 26.4%), asphyxiation (*n* = 40, 17.3%), sharp force trauma (*n* = 19, 8.2%), and firearm injuries (*n* = 18, 7.8%). Other causes included suspected poisoning (*n* = 11, 4.8%), bite injuries (*n* = 10, 4.3%), and fire/explosion (*n* = 2, 0.9%) (Supplementary Material 3D). Firearm-related injuries were relatively rare in comparison to findings from other studies [22, 26, 30, 39–42], which may reflect strict Dutch firearm regulations as well as pre-submission triage by law enforcement. In some instances, firearm-related deaths may be resolved at the scene due to overt macroscopic findings, resulting in non-submission for forensic necropsy. Suspected poisoning was infrequently assigned based on gross necropsy findings. This may partly reflect limited availability of toxicological confirmation, as biological samples were not consistently submitted by law enforcement agencies for further analysis. As a result, the true frequency of toxicologically confirmed poisoning cannot be assessed from the present dataset.

**Fig. 9:**
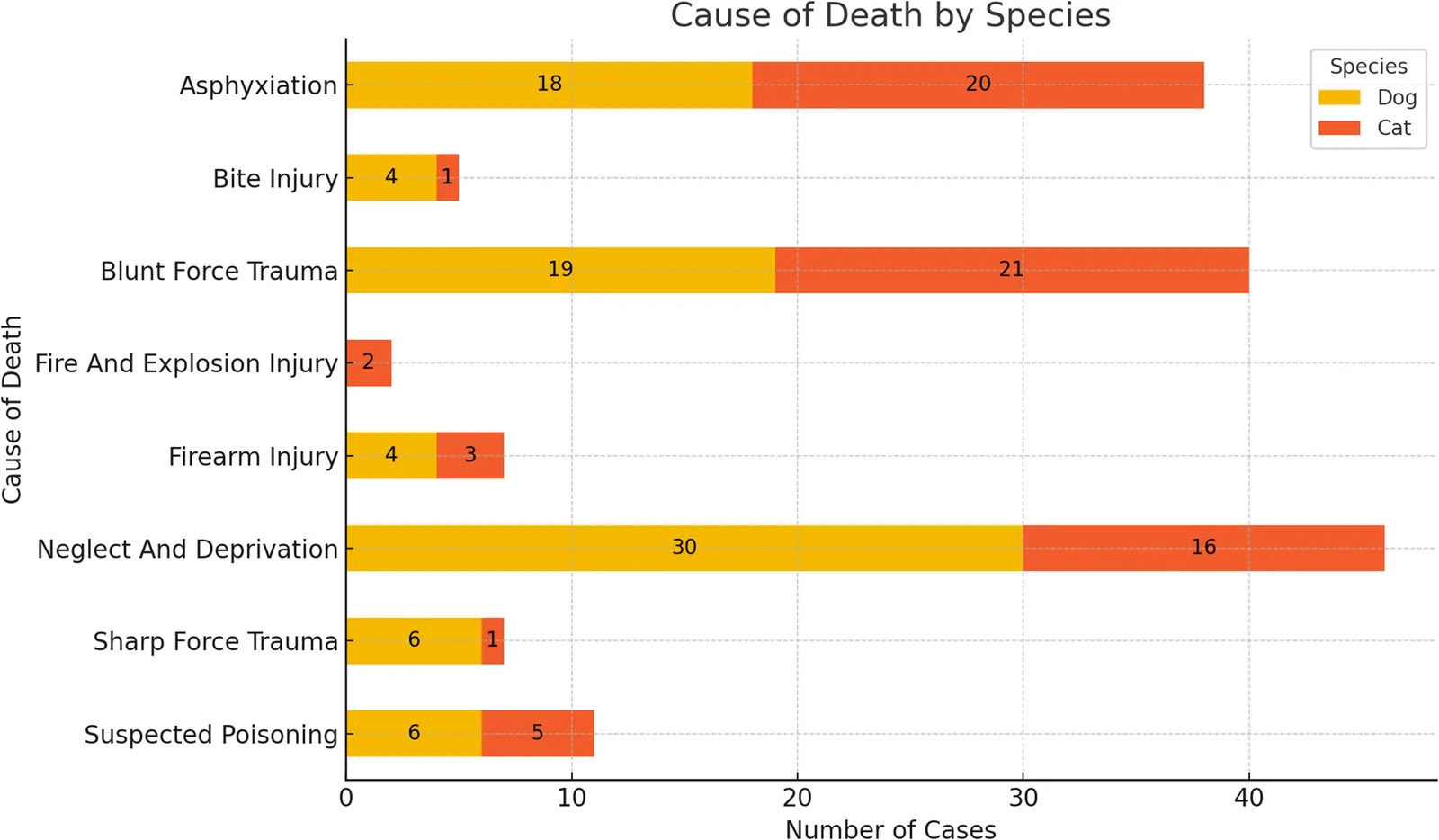
Distribution of presumptive non-natural causes of death by species, showing the number of cases (*N* = 156) per cause for dogs (light orange) and cats (dark orange)

Figure 9 shows the distribution of presumptive non-natural causes of death for cats and dogs. Blunt force trauma affected both dogs (*n* = 19) and cats (*n* = 21). Neglect/deprivation was most common in dogs (*n* = 30) but also significant in cats (*n* = 16). Asphyxiation was evenly distributed (dogs: *n* = 18; cats: *n* = 20). Sharp force trauma occurred more in dogs (*n* = 6 vs. *n* = 1), and fire/explosion injuries were rare (cats only, *n* = 2). Firearm injuries, bite injuries, and suspected poisoning were present in both species but low in number. A chi-square test showed no significant association between species and presumptive cause of death (*p* = 0.18), indicating similar cause distributions across dogs and cats.

Overall, these distributions represent presumptive classifications based on gross necropsy findings and should be interpreted within the methodological limitations of the study.

#### Trends in non-natural cause of death

**Fig. 10:**
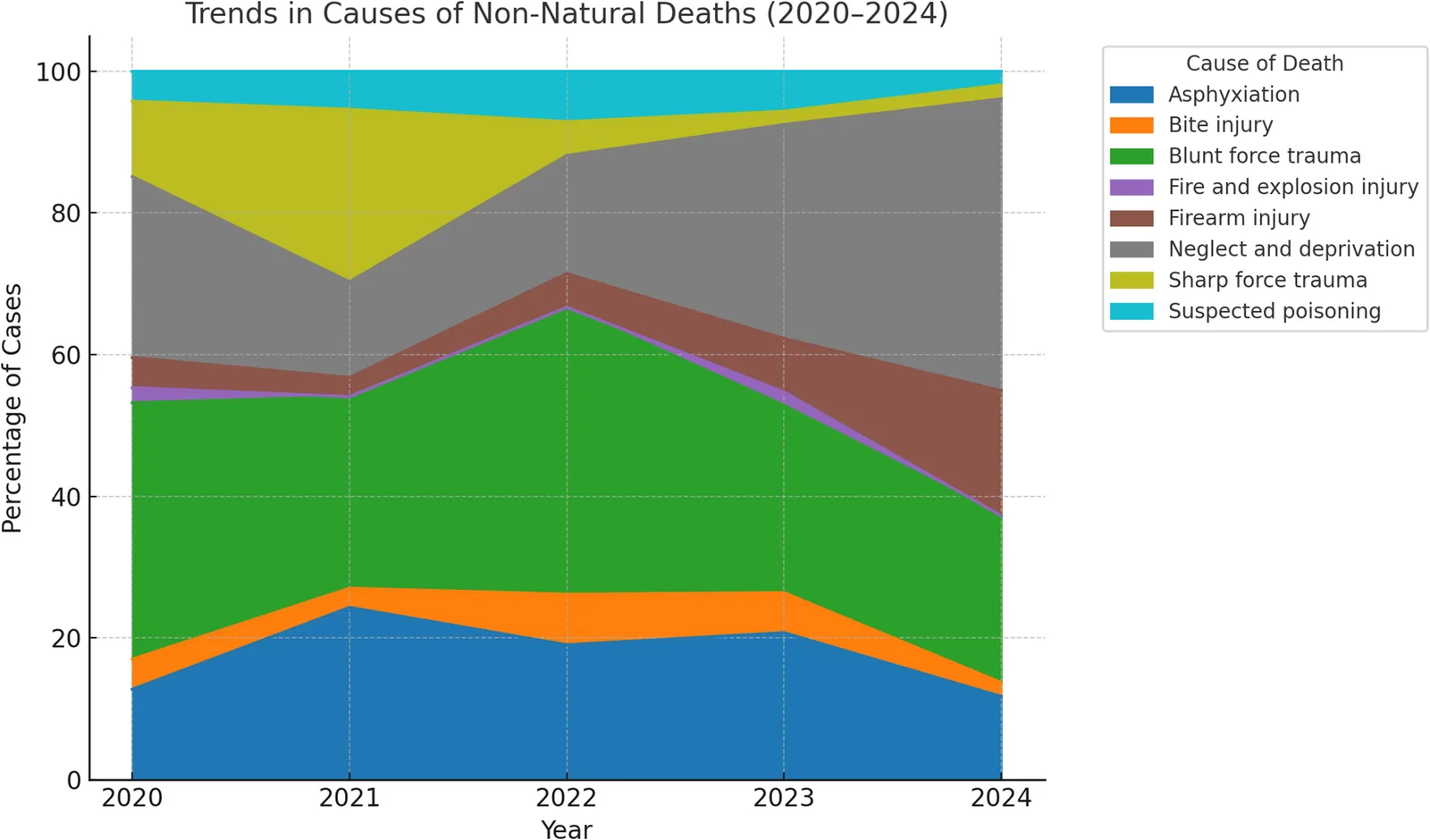
Stacked area chart showing trends in presumptive causes of non-natural deaths among submitted forensic cases (2020–2024). Data are shown as percentage of total cases (*N* = 231) per year

Figure 10 shows yearly trends in presumptive non-natural causes of death from 2020 to 2024 (*N* = 231). Blunt force trauma was the most frequently assigned category early on, peaking at 40.5% in 2022, then declining to 23.5% by 2024. Neglect and deprivation rose sharply, from 13.5% in 2021 to 41.2% in 2024, becoming the leading cause that year. Firearm injuries spiked in 2024 (17.6%), while asphyxiation, bite injuries, and suspected poisoning remained low and stable.

The increase in neglect may reflect post-pandemic pet ownership, where some owners struggled to provide adequate care [33], and rising veterinary costs, up ~ 40% over five years in the Netherlands [43, 44], leading to delayed or foregone care [33]. The decline in sharp force trauma may indicate substitution by firearms, possibly attributable to availability or perceived efficiency.

Logistic regression with Bonferroni correction showed only sharp force trauma had a statistically significant decreasing trend (*p* = 0.0043, adjusted *p* = 0.034). Increases in neglect and firearm injuries were not significant after correction (adjusted *p* = 0.164 and 0.114). Other causes remained stable. While overall distributions were largely consistent, the rise in neglect and firearm injuries highlights emerging concerns and the need for ongoing monitoring and preventive strategies.

Although these patterns are consistent with international veterinary forensic literature, the absence of histopathology and ancillary testing means that the relative frequencies reported here should be viewed as indicative rather than definitive.

#### Geographical pattern of non-natural causes of death

**Fig. 11:**
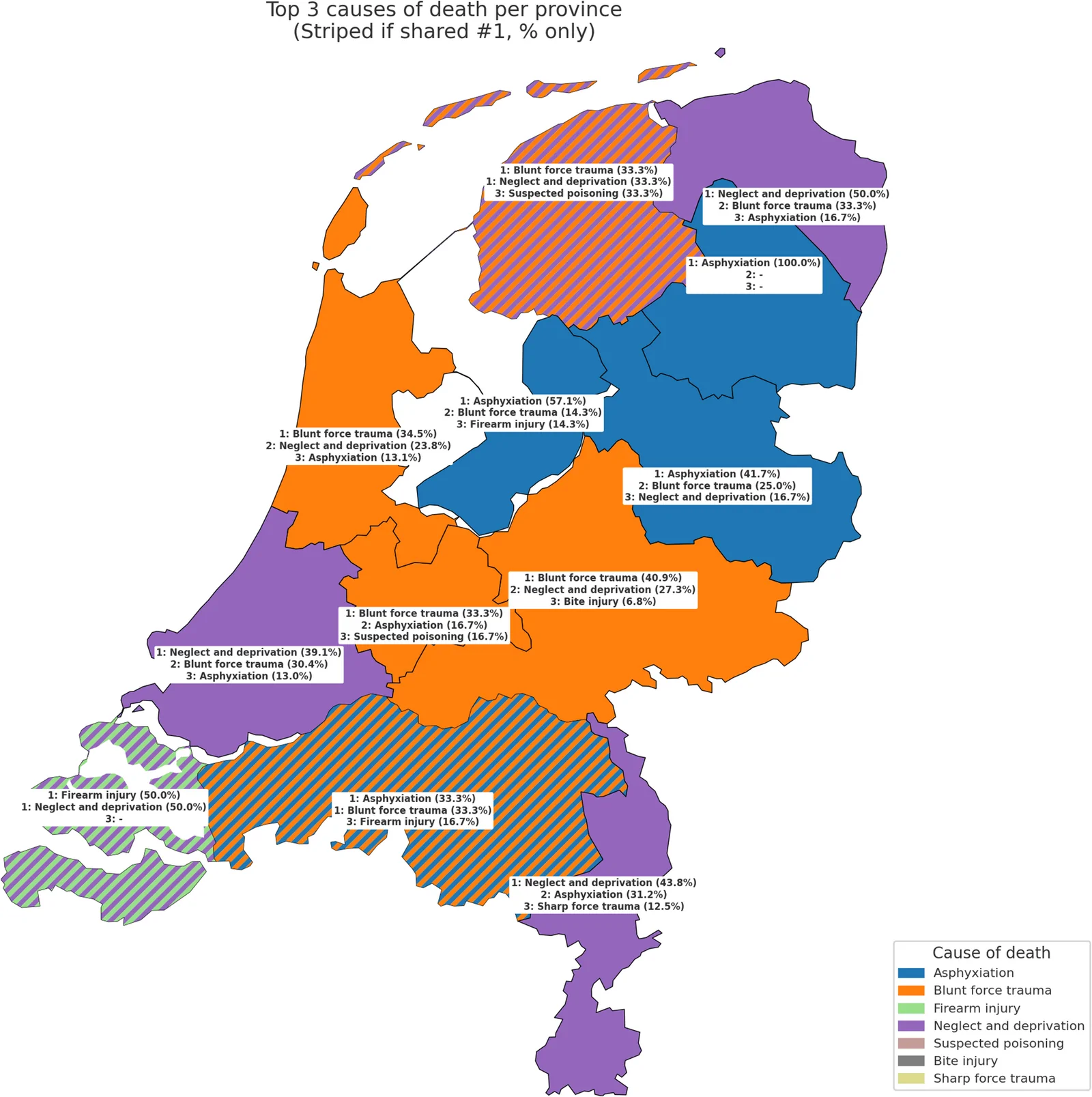
Top three presumptive non-natural causes of death per province (*N* = 231). Striped shading indicates provinces where two causes shared the top rank. Percentages reflect the proportion of cases within each province

Figure 11 shows the top three presumptive non-natural causes of death per province (*N* = 231). While visually some regional variation appears - blunt force trauma predominating in Noord-Holland, Gelderland, and Noord-Brabant, and neglect in Friesland, Limburg, and Zeeland - statistical analysis found no significant differences (χ² = 90.90, *p* = 0.284). Standardized residuals did not exceed |2|, indicating no cause was significantly over- or underrepresented in any province [45]. This suggests that, despite potential species differences by region, the types of presumptive non-natural deaths are broadly consistent across the Netherlands, supporting a uniform approach to forensic investigation and intervention strategies. These findings describe patterns in submitted cases rather than provincial differences in the true incidence of non-natural deaths, because normalization by relevant denominators was not possible.

### Species, age, and sex

Identifying trends in the type of animals involved in forensic cases can help clarify victim profiles and inform prevention strategies. Analysing species, breed, age, and sex can reveal whether specific populations (e.g., young or female animals) are disproportionately affected.

#### Species

Among presumptive non-natural deaths (*N* = 231), dogs were most frequent (*n* = 87, 37.7%), followed by cats (*n* = 68, 29.4%), reflecting patterns seen in other European and American studies [22, 25, 26, 30, 38, 40, 41, 46]. Dogs’ higher visibility in public may increase reporting, while cats’ tendency to hide when injured likely reduces submissions. Together, companion animals made up over two-thirds of non-natural deaths. Birds (*n* = 34, 14.7%) and rodents (*n* = 19, 8.2%) were less common, and livestock (*n* = 18, 7.8%) and wild animals (*n* = 5, 2.2%) were rare (Supplementary Material 3E). These lower frequencies may reflect differences in detection, reporting, or submission practices - particularly for wildlife or farm animals, which are often managed or perceived differently from pets. This distribution aligns with broader patterns of animal ownership and human-animal interaction, where companion animals are more closely monitored and more likely to be submitted for forensic evaluation following suspicious or unexplained death [22, 25, 26, 30, 38, 40, 41, 46].

#### Geographical pattern of submitted species

**Fig. 12:**
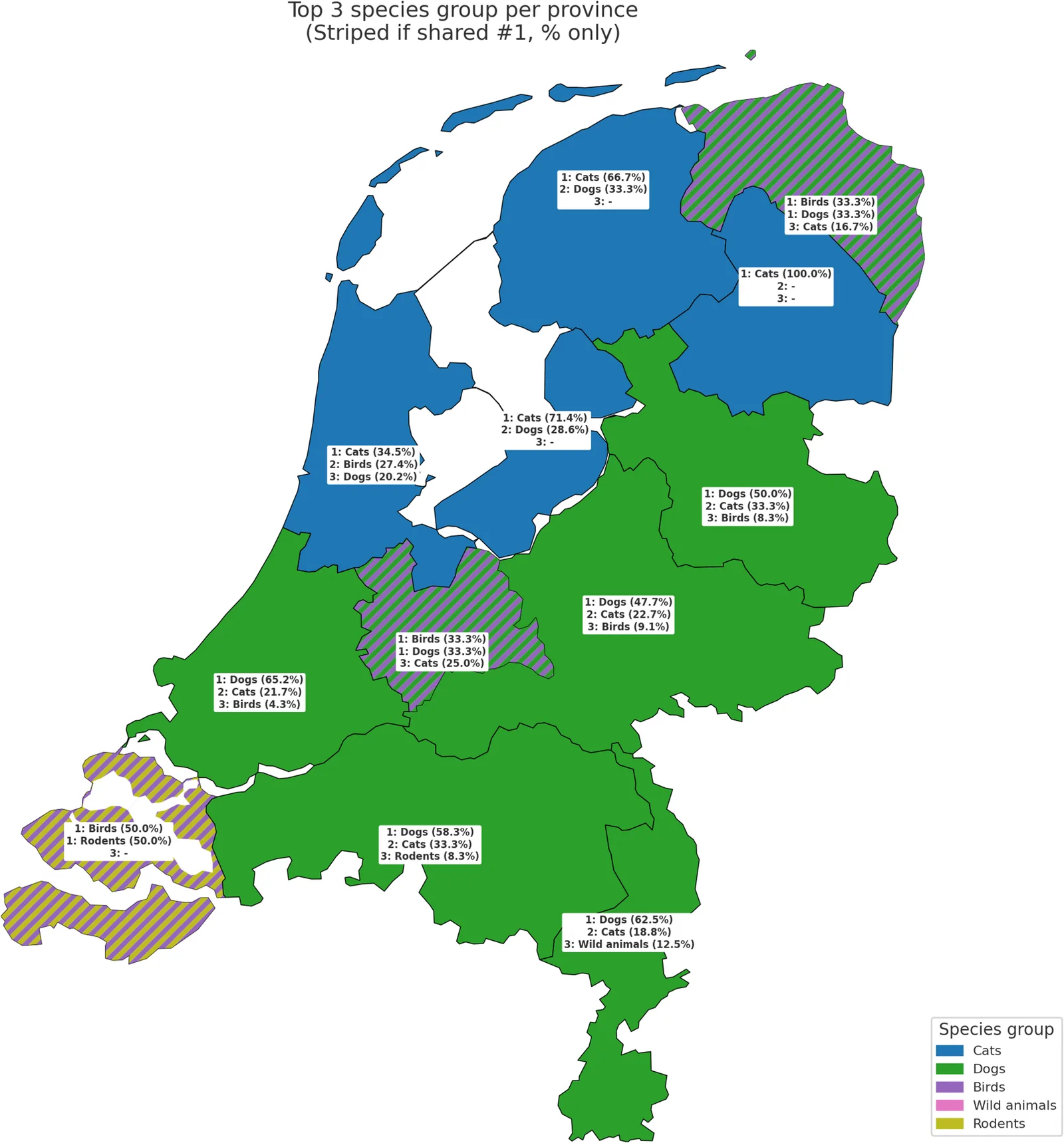
Top three submitted species groups per province (*N* = 231). Striped shading indicates shared first place. Percentages reflect the proportion of cases within each province

Figure 12 shows the top three submitted species with presumptive non-natural manner of death per province (*N* = 231). Dogs dominated in southern and eastern provinces (47.7% in Gelderland to 65.2% in Zuid-Holland), while cats were most common in northern provinces such as Friesland (66.7%), Drenthe (100%), and Overijssel (71.4%). Birds were more frequent in coastal or urbanized provinces (Noord-Holland, Zeeland), and wild animals or rodents appeared only in the top three in southern provinces (Limburg, Zeeland, Noord-Brabant).

A chi-square test confirmed a significant association between province and species (χ² = 80.12, *p* = 0.042). Standardized residuals identified specific deviations: Flevoland had more cats than expected (2.01), Groningen and Limburg more wild animals (2.37, 2.75), and Noord-Holland more birds (2.45) but fewer dogs (–2.69). These findings describe differences in the composition of submitted forensic cases across provinces. As population denominators and species distribution data in the general animal population were not available, no inference can be made regarding underlying incidence. The observed variation may reflect differences in submission patterns, reporting practices, or case selection processes across regions, but these factors cannot be disentangled within the present dataset.

#### Temporal trend in submitted species

**Fig. 13:**
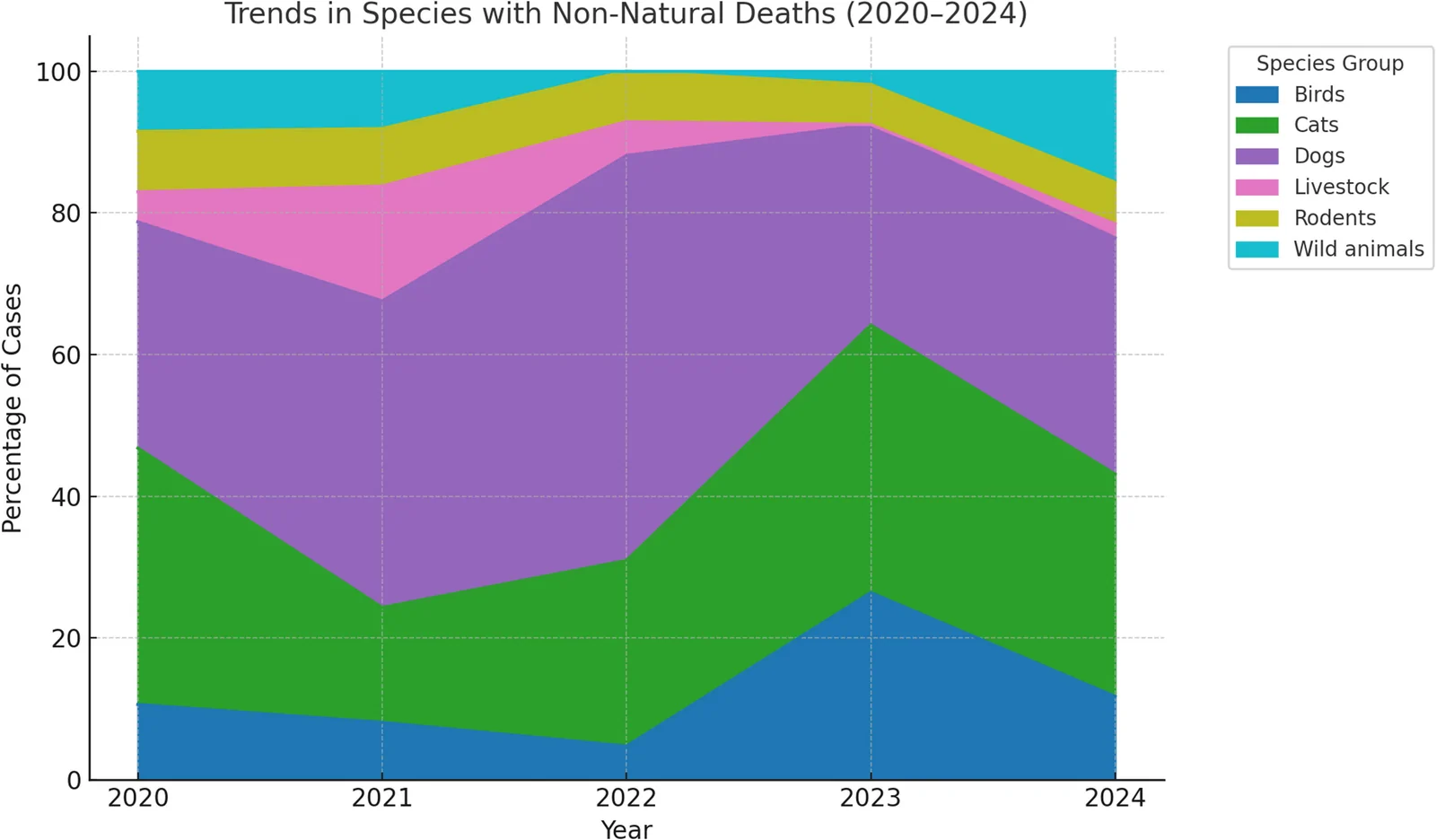
Stacked area chart showing the percentage distribution of species groups among cases with presumptive non-natural deaths from 2020 to 2024 (*N* = 231)

Figure 13 shows the annual distribution of species among presumptive non-natural deaths (*N* = 231). Dogs and cats consistently dominated submissions, peaking at 57.1% for dogs in 2022 and 37.7% for cats in 2020, but both fluctuated without clear trends. Other species showed more notable short-term shifts: birds increased to 26.4% in 2023 before declining, wild animals rose to 15.7% in 2024 after near absence from 2021 to 2023, livestock were notable in 2021 (16.2%) but declined thereafter, and rodents remained low and stable.

Logistic regression with Bonferroni correction showed no statistically significant trends for any species over the five-year period. The strongest, though non-significant, change was a decrease in livestock (*p* = 0.0602; adjusted *p* = 0.361). These findings suggest that while year-to-year shifts in species composition are apparent, they do not represent consistent, statistically verifiable trends over time. However, the visual increase in wild animals and birds in recent years may still warrant attention in future monitoring.

#### Case selection by species

Total submissions (*N* = 346) were compared with cases presumptively classified as non-natural deaths (*N* = 231) by species. Rodents (82.6%), livestock (62.1%), and birds (58.6%) showed high rates of non-natural findings, whereas wild animals (25.0%) and dogs (47.5%) were lower, which may reflect that some species, particularly wild animals, are often submitted despite limited forensic or judicial yield. In contrast, submissions for rodents and livestock appear more targeted, which may reflect clearer evidence of human involvement. A chi-square test indicated no statistically significant differences between species (χ² = 5.68, *p* = 0.339), though these patterns may still inform case selection, resource allocation, and triage in forensic practice.

#### Breed

Among cats with presumptive non-natural deaths (*N* = 68), European Shorthairs dominated (*n* = 47, 69.1%), with other breeds at 22.1% (*n* = 15) and 8.8% undetermined (*n* = 6), which is in line with previous studies in other countries [22, 26].

**Fig. 14:**
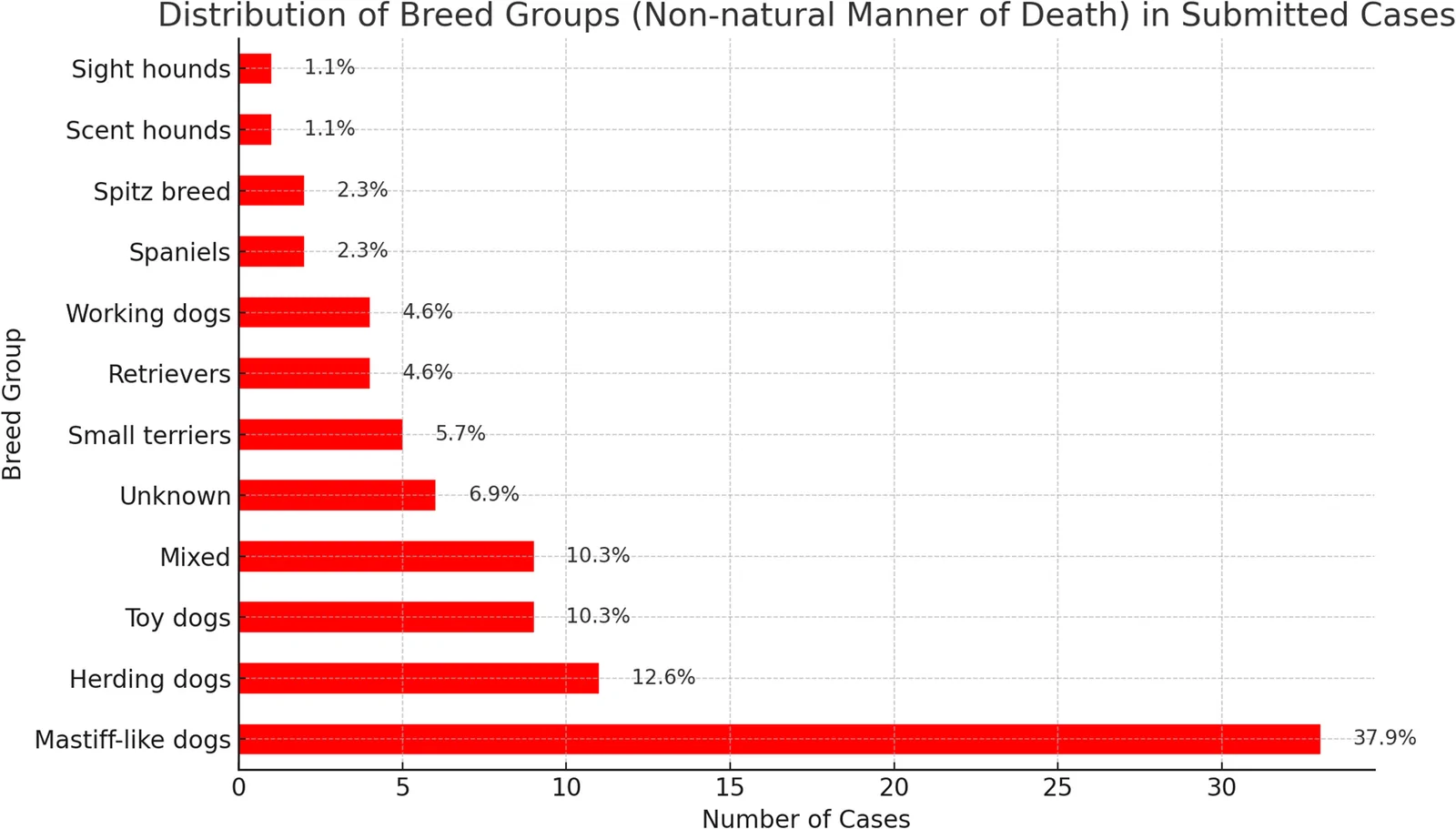
Distribution of dog breed groups in forensic cases with a presumptive non-natural manner of death (*N* = 87). Percentages reflect the proportion of each group among all submitted dog cases

For dogs (*N* = 87), mastiff-like breeds were most represented (37.9%, *n* = 33) (Fig. 14). This overrepresentation is consistent with evidence indicating dog breeds considered potentially dangerous are more vulnerable to abuse and is in line with similar studies in other countries [22, 25, 26]. Notably, many of the breeds in this category are commonly associated with illegal dogfighting practices [39, 47], which further underscores their vulnerability to abuse. Herding dogs accounted for 12.6% (*n* = 11), possibly reflecting traits such as high reactivity and excitability that may increase conflict with owners [48]. Toy breeds represented 10.3% (*n* = 9), possibly reflecting a combination of physical fragility and higher ownership in urban or socioeconomically disadvantaged settings, which are associated with increased abuse risk [49]. Scent and sight hounds were least represented (1.2%, *n* = 1 each), and breed could not be determined in 6.9% (*n* = 6), indicating limitations in breed identification based on postmortem material or incomplete documentation. The categorization was based on the breed groups listed in [50].

These patterns may indicate potential breed-related risk factors or biases in submission and reporting, but interpretation should consider confounding factors such as breed popularity, population distribution, lifestyle, and socioeconomic context.

#### Age

Among 149 presumptively abused animals of which age could be estimated, 13.4% were juveniles (*n* = 20), 79.9% adults (*n* = 119), and 6.7% seniors (*n* = 10), indicating a predominance of adults. A Shapiro-Wilk test (W = 0.82, *p* = 0.159) and Q-Q plot suggested no significant deviation from normality, though caution is warranted given only three age categories. This pattern aligns with studies from other countries reporting mostly adult abuse victims [22, 38, 51], possibly reflecting longer exposure to abusive environments. Several other studies suggest that younger animals may be especially vulnerable to abuse [25, 51, 52]. Young animals are often described as more restless, less manageable, and more likely to irritate their owners, potentially provoking aggressive responses. They are also physically more fragile and less capable of defending themselves or fleeing. Additionally, their exploratory behaviour may increase their risk of encountering external threats, such as aggression from neighbours [25]. The current findings do not contradict these concerns but also do not provide evidence for a disproportionate risk in any specific age group. Importantly, in the absence of reference data on the general age distribution of the pet population in the Netherlands, no firm conclusions can be drawn regarding over- or underrepresentation. The distribution observed here may reflect the true distribution of age groups among animals in the general population, or it may not.

To investigate whether age category is associated with the presumptive cause of death, a chi-square test was conducted. The results yielded a p-value of 0.88, indicating that there is no statistically significant association between age category and cause of death. This suggests that, based on the available data, age does not appear to be a determining factor in the cause of death within the sampled population.

While age estimation based on tooth eruption provides a practical approximation, it lacks the precision of imaging-based methods [53], which were not feasible in the present study.

#### Biological sex

Among cats with a presumptive non-natural cause of death with known sex (*N* = 52), 30 were male and 22 were female, which is in line with similar other studies [22, 25, 51]. This distribution also did not differ significantly from parity (*p* = 0.33). Among dogs with a presumptive non-natural cause of death with known sex (*N* = 78), 34 were male and 44 were female, which is different from some studies [22, 25, 26, 30], but in line with other studies [40, 51]. A binomial test showed no significant deviation from a 1:1 sex ratio (*p* = 0.31). These findings indicate that the sex distribution in both species did not significantly depart from an equal male-to-female ratio, which is consistent with previous studies [26, 30, 38]. Also, the relationship between sex and presumptive cause of death was tested. The chi-square test produced a p-value of 0.43, indicating that there is no significant correlation between sex and presumptive cause of death.

### Legal outcomes

Understanding the judicial outcomes of forensic animal cases is essential for evaluating the impact and relevance of veterinary forensic work within the broader criminal justice system. To assess legal outcomes of the forensic cases included in this study, such as prosecution status, sanctions, and case resolution, formal data requests were submitted to the Dutch police, the *Openbaar Ministerie* (Public Prosecution Service), and *De Rechtspraak* (Council for the Judiciary). These requests did not result in systematically accessible datasets, as institutions either declined data sharing, indicated that the information could not be provided, or did not respond within the study period. Moreover, as authors of official forensic reports, we receive no systematic feedback on legal outcomes.

As a result, systematic evaluation of legal outcomes based on institutional reporting was not possible. Instead, legal outcome data were obtained through an informal data request via the Dutch police, which formed the basis of the analyses presented below. Case matching was performed using case numbers provided in the forensic dataset and queried within the national police system. This process resulted in 80 matched cases; however, the reasons for the limited number of matches could not be determined from the available information. It is therefore unclear whether this reflects incomplete linkage between databases, differences in registration systems, or other administrative factors. Legal outcomes were not independently verified against court records, and 22 of the 80 matched cases were still under investigation or had not yet reached trial at the time of data extraction, meaning their outcomes may still change. All results presented in this section should be interpreted with these limitations in mind.

#### Legal outcomes

A total of 80 forensic animal cases could be matched in the Dutch national police system, of which 58 had resolved legal outcomes at the time of data extraction. The remaining 22 cases were still under investigation or had not yet reached trial.

**Fig. 15:**
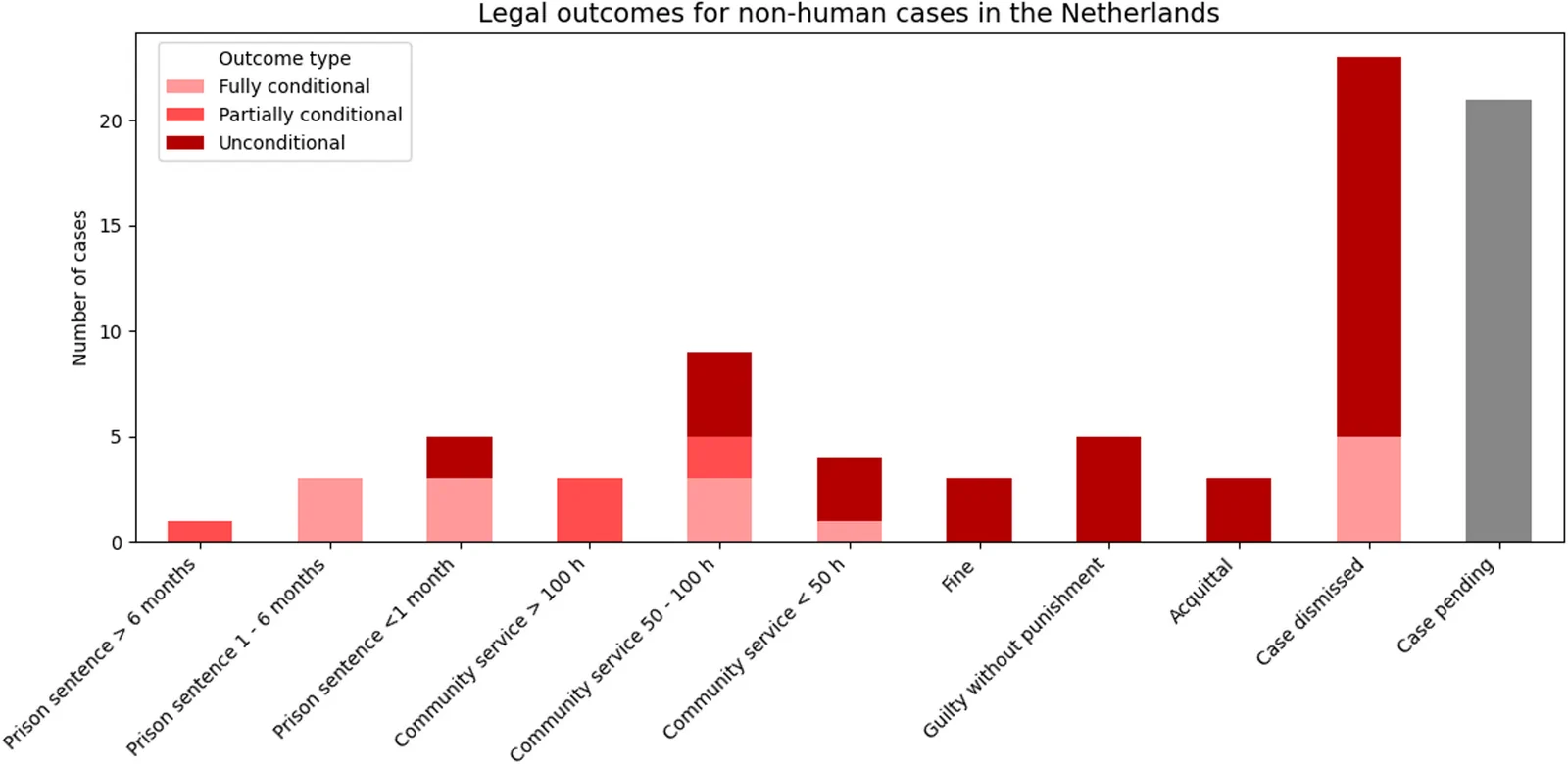
Distribution of legal outcomes for 80 submitted non-human cases that could be found in the Dutch national police system. Each stacked bar shows the number of cases per legal outcome classified as “fully conditional,” “partially conditional,” or “unconditional”

Among resolved cases, the most frequent outcome was dismissal (*n* = 23), followed by community service (50–100 h, *n* = 9) and short prison sentences (< 1 month, *n* = 5). Prison terms over six months were rare (*n* = 1). Most sentences were unconditional (*n* = 59), with fewer partially (*n* = 6) or fully conditional (*n* = 15) sentences), in which punishment was either partially or fully suspended on the condition of good behaviour or fulfilment of other legal obligations (Fig. 15).

Additional judicial measures were imposed in 13 cases, including bans on keeping animals (*n* = 8), which ranged in duration from two years to a lifelong ban, mandatory reporting to probation (*n* = 4), and compulsory forensic treatment (*n* = 1). These supplementary sanctions indicate recognition by the courts of the potential risk of recidivism or underlying psychological or behavioural issues in certain offenders.

Although some convictions were achieved, the penalties imposed in these cases were generally mild when compared to the maximum sentences allowed under Dutch animal welfare legislation. (Conditional) prison sentences, when given, were typically less than 6 months, and many cases resulted in (conditional) community service or fines. Even in instances involving severe violence or prolonged neglect, harsher penalties were rare.

#### Species and legal punishment

**Fig. 16:**
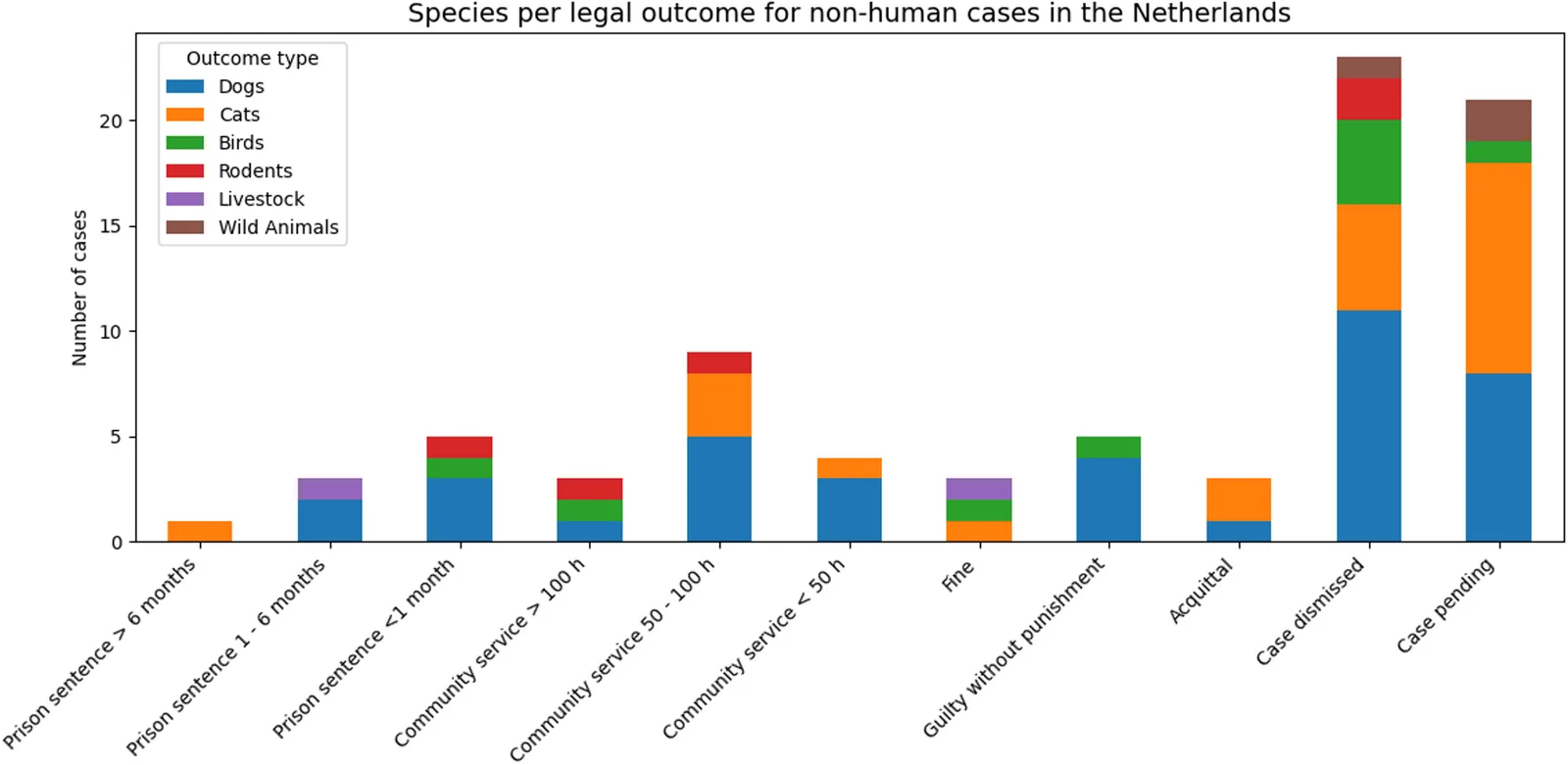
Distribution of legal outcomes for 80 submitted non-human cases that could be found in the Dutch national police system. Each stacked bar shows the animal species the case concerned

A total of 59 resolved cases were analysed to assess whether species was associated with legal outcome. Of these, 33 cases resulted in sanctions (e.g., fines, community service, imprisonment) and 26 did not (e.g., case dismissal, acquittal). Dogs were the most common species involved (*n* = 30), followed by cats (*n* = 13), birds (*n* = 8), rodents (*n* = 5), livestock (*n* = 2), and wild animals (*n* = 1). Among dog cases, 18 led to convictions. Chi-square analysis showed no significant association between species and legal outcome (χ²(5) = 3.18, *p* = 0.671) (Fig. 16). These results may reflect that, within the analysed subset, legal outcomes were not statistically different across species categories.

#### Cause of death and legal punishment

**Fig. 17:**
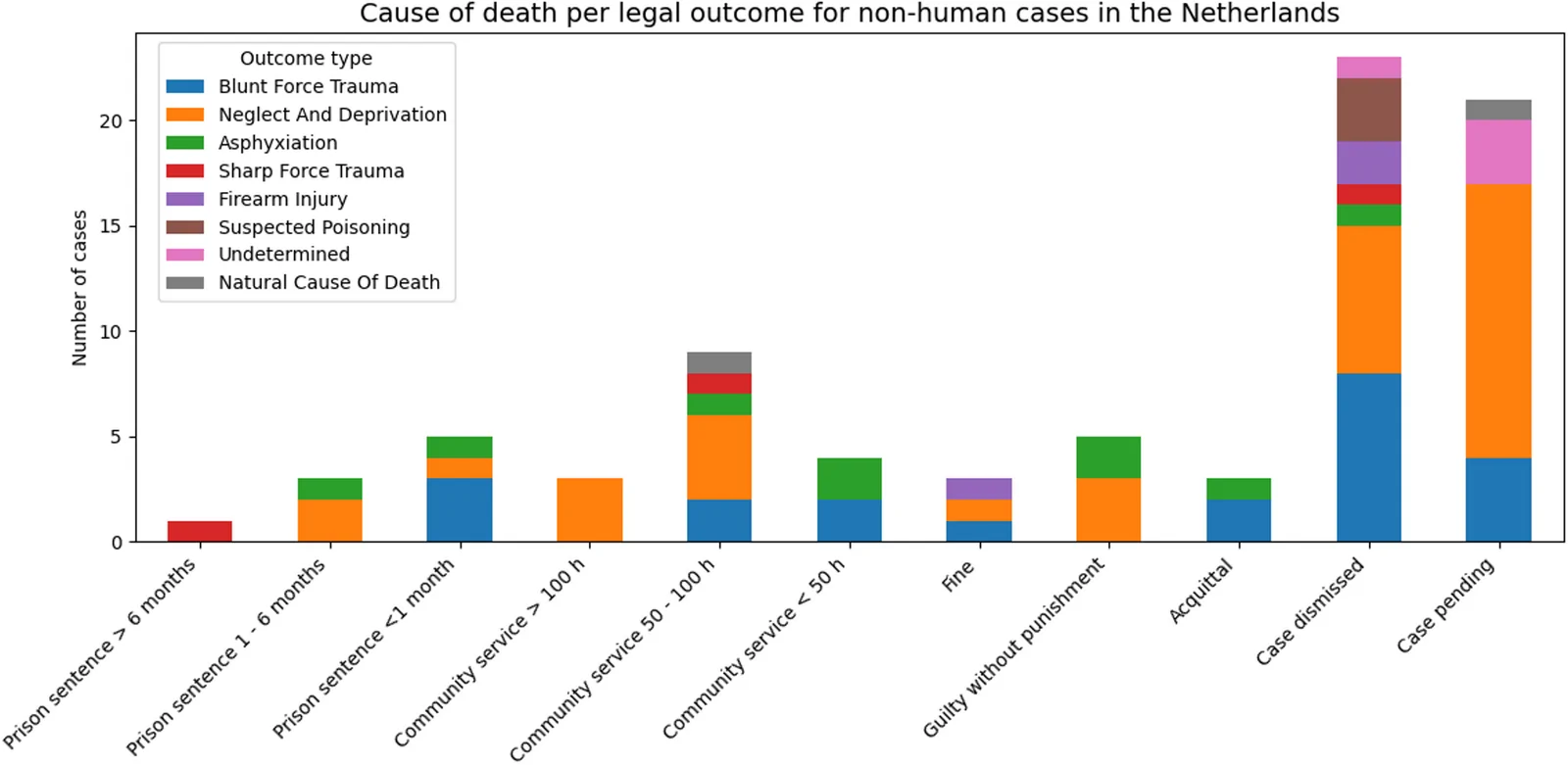
Distribution of legal outcomes for 80 submitted non-human cases that could be found in the Dutch national police system. Each stacked bar shows the presumptive cause of death reported by Stichting Forensisch Dierenonderzoek

Neglect and deprivation (*n* = 21), blunt force trauma (*n* = 18), and asphyxiation (*n* = 9) were the most common presumptive causes of death among resolved cases. Punishment was relatively frequent in asphyxiation (77.8%) and neglect (66.7%) cases, whereas only 44.4% of blunt force trauma cases and 33.3% of firearm injury cases led to sanctions (Fig. 17). Suspected poisoning cases were rarely punished, which may be attributable to evidentiary challenges in proving intent or causality, compounded by limited follow-up on toxicological samples collected for analysis or rejected by the Netherlands Forensic Institute.

Despite these differences, statistical analysis did not show a significant association between presumptive cause of death and legal outcome (χ²(5) = 8.13, *p* = 0.149). It is important to note that the lack of statistical significance does not equate to absence of effect; small sample sizes and case-specific factors, including evidence quality, prosecutorial discretion, and societal perceptions of species, likely influence outcomes.

## Future recommendations

This five-year retrospective study of veterinary forensic necropsies in the Netherlands identified recurring patterns in suspected animal abuse cases, as well as variability in operational practices and case handling procedures. The findings should be interpreted within the context of a developing forensic field in which standardisation and data integration are still evolving.

The majority of cases were submitted by police and governmental agencies. Variability was observed in packaging and storage conditions across submissions, including the use of non-compliant packaging and non-refrigerated storage in a subset of cases. While some improvement was observed in 2024, including increased use of sealed forensic body bags, the findings suggest heterogeneity in pre-submission handling practices. From a forensic perspective, greater consistency in submission, packaging, transport, and storage procedures may support more uniform preservation of evidentiary material. Development or refinement of national-level guidelines, in combination with appropriate logistical support and training, could contribute to standardisation of these processes [22, 29].

Veterinarians were infrequently represented among submitting parties in this dataset. Given their position in animal healthcare systems and their legal obligation to report suspected animal cruelty, this low submission rate may reflect structural or procedural factors such as uncertainty regarding reporting pathways or variability in engagement with investigative authorities. Previous studies have similarly identified uncertainty regarding reporting procedures and legal thresholds as potential barriers [7, 36]. Further work could explore how reporting processes and inter-agency communication influence veterinary involvement in forensic case initiation.

Standardised protocols for submission and handling of animal remains may support more consistent case processing across provinces. Such protocols would ideally be complemented by appropriate infrastructure (e.g., refrigerated storage capacity, standardised packaging materials) and training initiatives across veterinary, law enforcement, animal welfare, and judicial sectors, with emphasis on evidence preservation and interdisciplinary communication.

Future cases would benefit from incorporating ancillary testing, such as histopathology, toxicology, and microbiology, particularly when relevant samples have been collected and are available for further analysis. This would improve diagnostic certainty and strengthen the forensic and scientific interpretation of findings.

To promote systematic knowledge sharing, we also recommend the establishment of a centralized national database of veterinary forensic cases. Such a system, modelled after successful examples in Spain, Brazil, and the United Kingdom, would allow for trend analysis, geographic monitoring, identification of repeat offenders, and evaluation of long-term preventive strategies [24, 25]. At present, veterinary forensic data in the Netherlands are not centrally aggregated, which limits systematic evaluation of trends over time and across regions. Integration of veterinary forensic information into broader public health or violence-related datasets may further support interdisciplinary research, particularly given the documented associations between animal abuse and interpersonal violence [2, 5].

In selected cases, early involvement of veterinary forensic experts at the scene may provide additional contextual information relevant to postmortem interpretation, such as environmental conditions, decomposition stage, and entomological activity. This would allow for example for entomological sampling and more accurate postmortem interval (PMI) estimations, which are otherwise nearly impossible without firsthand environmental context.

Further research is warranted into methodological tools specifically designed for veterinary forensic applications, including postmortem interval (PMI) estimation techniques adapted from human forensic science. Approaches such as temperature-based models [54–56], entomological analyses, and decomposition scoring systems may benefit from further validation in animal cases.

Finally, sustainable institutional support, dedicated funding, and formal integration of veterinary forensic science into national forensic frameworks would help ensure consistency, reduce reliance on ad-hoc arrangements, and maximize the contribution of these investigations to animal welfare enforcement and broader public safety efforts.

## Conclusion

This five-year retrospective analysis of veterinary forensic necropsies in the Netherlands provides a detailed view of patterns of animal abuse and the current state of forensic practice.

Dogs and cats represented the majority of submitted cases, with blunt force trauma and neglect identified as the most frequently assigned categories based on gross necropsy findings. A substantial proportion of cases involved advanced decomposition, which limited the ability to assign definitive cause or manner of death in a subset of submissions.

Variation was observed in the geographic distribution of cases, which likely reflects differences in submission patterns and reporting practices. Urban areas accounted for a larger proportion of submissions compared with rural areas, although no inference can be made regarding underlying incidence.

Most cases were submitted by police and governmental agencies. Variability in packaging and storage practices was observed, although some improvement in standardisation was noted in later years of the study period.

Analysis of species, age, and breed revealed trends that may inform targeted prevention strategies. Mastiff-type breeds were overrepresented among submitted dog cases, and non-companion species such as birds, livestock, and rodents displayed varying levels of forensic relevance.

Legal follow-up was available for a subset of cases only. Within this subset, a range of outcomes was observed, including dismissals and a limited number of sanctions. These findings describe case outcomes within the available dataset and may not represent all legal proceedings related to veterinary forensic submissions.

Overall, this study demonstrates the critical value of veterinary forensic necropsy in identifying, documenting, and understanding animal abuse. To fully realize its potential, the field requires sustainable funding, national guidelines for evidence handling, formal integration into investigative protocols, and interdisciplinary training. Strengthening these elements will improve the legal impact of forensic findings, enhance animal welfare enforcement, and contribute to broader violence prevention and public safety efforts.

## Supplementary Information

Below is the link to the electronic supplementary material.

Supplementary Material 1 (DOCX 19.9 KB)

Supplementary Material 2 (DOCX 23.0 KB)

Supplementary Material 3 (DOCX 673 KB)

Supplementary Material 4 (PDF 4.80 MB)

## Data Availability

The data underlying this study consist of forensic necropsy case records collected over a five-year period. Due to the confidential and sensitive nature of these data, including legal and court-related information, the dataset is not publicly available. Aggregated data supporting the findings of this study may be available from the corresponding author upon reasonable request and subject to applicable legal, ethical, and institutional restrictions.

## Declarations

### Generative AI in scientific writing

During the preparation of this work the authors used ChatGPT, an artificial intelligence developed by OpenAI, in order to assist in their redaction and data analysis. After using this tool/service, the authors reviewed and edited the content as needed and take full responsibility for the content of the publication.
