# Supplementary material for: Five years of veterinary forensic necropsies in the Netherlands: a retrospective analysis of non-human cases
Source: Int J Legal Med. 2026 Jun 3;140(5):2877–901. doi: 10.1007/s00414-026-03840-y (PMC13499755; doi:10.1007/s00414-026-03840-y)
Supplement: Supplementary file 1 [file 414_2026_3840_MOESM4_ESM.pdf]

POLITIE

D.C.G

Dieren binnen Criminaliteit en G.G.P.

# Handelingskader Forensische Opsporing m.b.t. DIEREN

*"Als de mens luistert naar zijn diepste gevoelens, weet hij dat hij goed moet zijn voor dieren, want hij die wreed is tegen dieren, is ook hardvochtig voor mensen. Wij kunnen het hart van een mens beoordelen, door naar zijn behandeling van dieren te kijken."*
*Bron: Immanuel Kant 1724-1804.*

Versie 4 2023

# Handelingskader voor FO voor Dieren binnen Criminaliteit en Gebied Gebonden Politie


- **Voorwoord** — **Pagina 2/4**
- **Inzetcriteria** — **Pagina 5**
- **Verpakken van een kadaver** — **Pagina 6**
- **Stappenplan na veiligstellen kadaver** — **Pagina 7**
- **Vraagstelling PV aanvraag benoeming deskundige** — **Pagina 8**
- **Sectie op het kadaver** — **Pagina 9**
- **Voorbeeld rapportage Drs. Monique Verkerk** — **Pagina 10/12**
- **Forensisch onderzoek levende dieren** — **Pagina 13/15**
- **Wetgeving en bevoegdheden** — **Pagina 16/22**
- **Bestialiteit (seks met dieren)** — **Pagina 23/24**
- **Honden/dieren gevechten** — **Pagina 25/27**
- **Signalen dier, eigenaar en leefomgeving** — **Pagina 28/30**

**Bijlage:** **Curriculum Vitae Drs. Monique Verkerk**
**Veterinaire verklaring sporen bij levende dieren**
**Protocol verpakken**

# Voorwoord

Dit handelingskader is geschreven voor de collega's die werkzaam zijn binnen de Forensische Opsporing. Omdat alle forensisch onderzoekers op eenzelfde wijze moeten kunnen handelen, is er in het kader van informatievoorziening een handelingskader opgemaakt. De bewijsvoering in de rechtbank wordt ook in dierenzaken steeds belangrijker, waardoor de verantwoording van de chain of custody en chain of evidence veel belangrijker is dan voorheen. Voorts is het belangrijk om de professionaliteit van de Forensische Opsporing op deze wijze uit te dragen.

In het gedoogakkoord van 30 september 2010, werd er tussen de VVD, het CDA en de PVV afgesproken dat er vijfhonderd Animal Cops moesten worden aangesteld. Echter in 2012, door een wijziging in de tweede kamer, werd deze fulltime functie omgezet in een taakaccent, Dierenwelzijn. In januari 2013 is de Wet Dieren (WD) in werking getreden. Deze wet maakt het mogelijk om personen die worden verdacht van misdrijven en overtredingen die worden gepleegd met dieren, strafrechtelijk te vervolgen. De Wet Dieren is een kaderwet. Een kaderwet is een wettelijke basis die algemene richtlijnen, verantwoordelijkheden en procedures regelt, maar die nader wordt ingevuld met algemene maatregelen van bestuur. Binnen de Nationale Politie is de aanpak van verwaarlozing en mishandeling van dieren, evenals de noodhulp aan dieren, geborgd binnen het reguliere politiewerk. De werkzaamheden zijn verankerd in het basisteam. Dierenwelzijn behoort daarmee tot de generieke taak van alle medewerkers van het basisteam. De meldingen die de politie krijgt op het gebied van dieren zijn:

- Het mishandelen en doden van dieren
- Bijtincidenten
- Het verwaarlozen van dieren
- Dieren (honden) in te warme auto's
- Seks met dieren
- Hondengevechten
- Dumpen van dieren
- Hondenhandel

De Forensische Opsporing (FO) zal niet bij alle bovenstaande incidenten worden gevraagd om een sporenonderzoek uit te voeren. In het geval van het achterlaten van een hond in een te warme auto zal de FO niet zoveel meerwaarde hebben. In het geval van mishandeling, het doden, ontucht met dieren en dierenhandel kan de expertise van de Forensische Opsporing van groot belang zijn in het strafrechtelijk onderzoek. Afgesproken is, dat in principe de FO in kennis wordt gesteld van een incident waarbij dieren ernstig zijn mishandeld en/of zijn komen te overlijden door toedoen van mensen en dan zelf beoordelen of zij ter plaatse gaan. In artikel 67 van het Wetboek van Strafvordering zijn er enkele artikelen uit de Wet Dieren bij naam genoemd waardoor op bijvoorbeeld dierenmishandeling, zoals beschreven in artikel 2.1 WD, voorlopige hechtenis is toegelaten. Onderzoek toont aan, dat daders van geweldincidenten, zich in veel gevallen eerst schuldig hebben gemaakt aan dierenmishandeling. Dit varieert van vuurwerk in de anus van een levend dier tot aan pijlen door een kop van een vogel schieten. Adequaat handelen is daarom belangrijk om de kans op herhaling van misdrijven in welke vorm dan ook te voorkomen.

### Landelijke werkgroep Forensisch Dieronderzoek

Er is een Landelijke werkgroep Forensisch Dierenonderzoek. Deze werkgroep valt onder het Kwaliteitsnetwerk Forensisch Medische zaken. Het streven is dat alle Politie-eenheden met ten minste één contactpersoon, die werkzaam is bij de Forensische Opsporing, deelneemt aan deze werkgroep. Verder zijn er ook een tactisch coördinatoren aangesloten bij de werkgroep. Als er vragen met betrekking tot forensisch dieronderzoek zijn kan er altijd contact worden opgenomen met de onderstaande contactpersonen. Ook is er een landelijke Agora website DCG. Door de landelijke werkgroep wordt er onderzoek gedaan naar het veiligstellen van sporen bij dieren. Zo heeft een onderzoek uitgewezen dat een bemonstering van een vacht, bij sommige diersoorten een beter DNA-profiel oplevert met behulp van een stub, dan met een swab. Ook is er met diverse technieken geprobeerd vingerafdrukken van een vleugel van een duif veilig te stellen. Er is een bruikbaar dactyloscopisch signalement veiliggesteld op veren met behulp van fluorescerend magnetisch poeder.

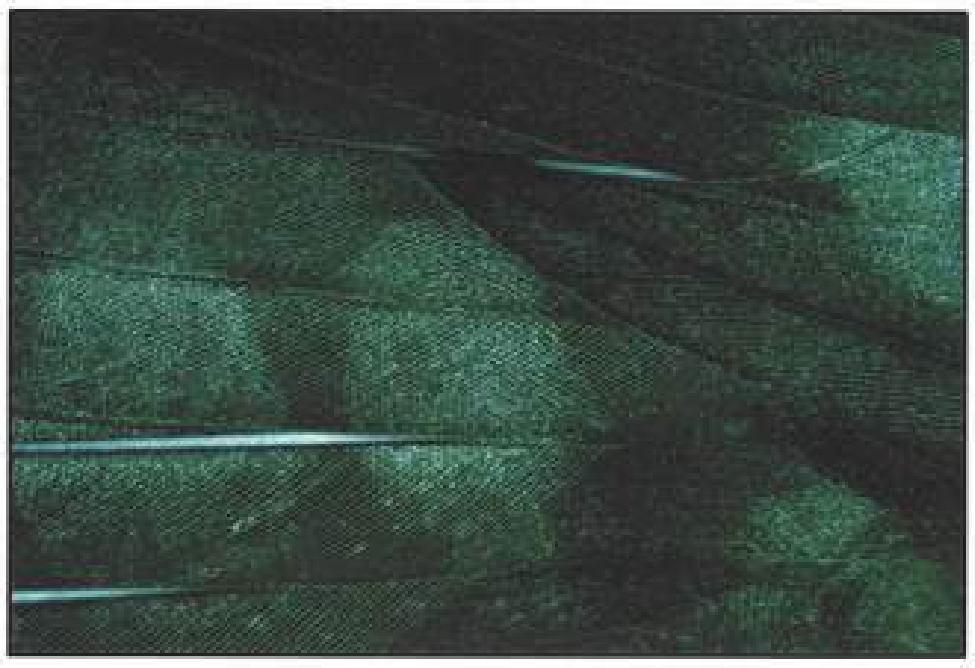

**Contactpersonen DCG FO Landelijk**

- Kwaliteitsnetwerk Forensisch-medische zaken - landelijke werkgroep Forensisch Dierenonderzoek

| | |
|---|---|
| **Eenheid Oost-Nederland** | Tamara van Appeldoorn<br>Kim_Zaaijer |
| **Eenheid Noord- Nederland** | Chantal Leentfaar<br>Marjolein Wessels_ |
| **Eenheid Noord - Holland** | Rianne de Munk<br>Raymond Maarsen en Marissa Brons |
| **Eenheid Amsterdam** | Andre Grazul |
| **Eenheid Rotterdam** | Diana Scheurwater |
| **Eenheid Limburg** | Wendy Gerritsen<br>Ingrid op den Camp |
| **Eenheid Den Haag** | Cees de Mol<br>Gerard Hoeke |
| **Eenheid Zeeland- West- Brabant** | Marleen Appelhof-Kroeze |
| **Eenheid Midden-Nederland** | |
| **Eenheid Oost-Brabant** | Kay Michiels<br>Esther Benthem |

Er wordt voorlichting gegeven over het forensisch sporenonderzoek met betrekking tot dieren in criminaliteit. Deze voorlichting wordt gegeven aan de dierenambulances en andere

organisaties/ketenpartners. De doelstelling van de voorlichting is dat men bewust wordt van de sporen die aanwezig kunnen zijn, zodat contaminatie kan worden voorkomen.
Ook is er goed contact met de taak accenthouders dierenwelzijn en de tactisch coördinator van de DCG in de Eenheid.

**Verpakking van dieren**
Er zijn pakketten gemaakt voorzien van: een verpakprotocol, diverse maten breathable bags/ ademende sealbags, een kinderlijkzak met sluitlabel, handschoenen en mondkapje, steriel laken en een Spoor Identificatie Nummer met label.
Deze pakketten worden de taakaccent houders DCG van de uitgereikt. In sommige eenheden ook bij de dierenambulance. In het protocol staat stap-voor- stap beschreven, welke handelingen moeten worden verricht. Ook aan het voorkomen van contaminatie is gedacht. Op deze wijze kan de chain of custody worden gewaarborgd, mocht de Forensische Opsporing niet ter plaatse kunnen gaan.
*Voorheen werden kadavers verpakt in gebruikte afdekzeilen. Door onder andere de dierenambulances en andere ketenpartners voorlichting te geven over contaminatie en het verpakken van een kadaver, kunnen er daarna nog sporen worden veiliggesteld.*

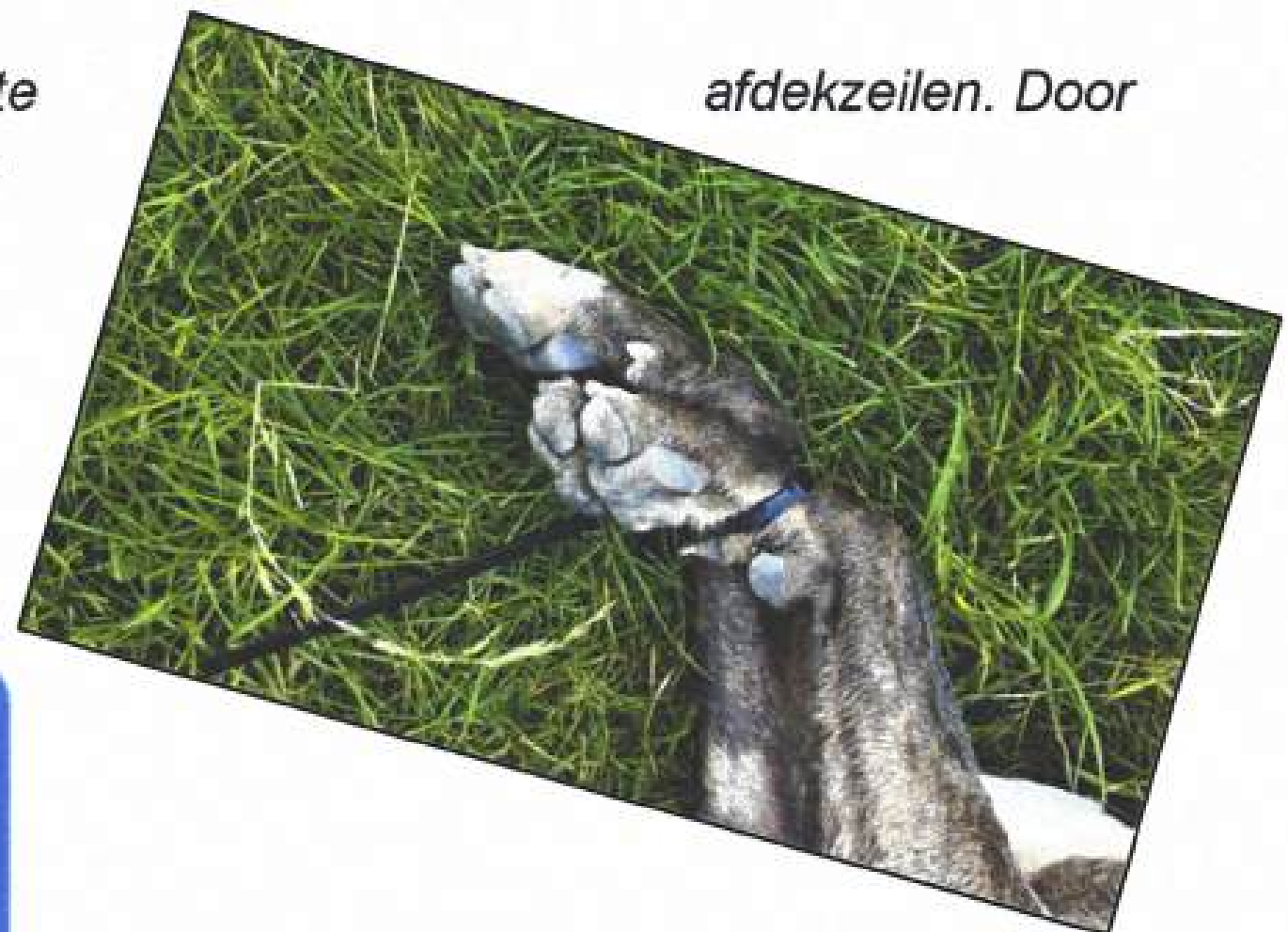

*Hond in sloot gedumpt. Zijn poten waren met een tie-wrap aan elkaar gebonden...*

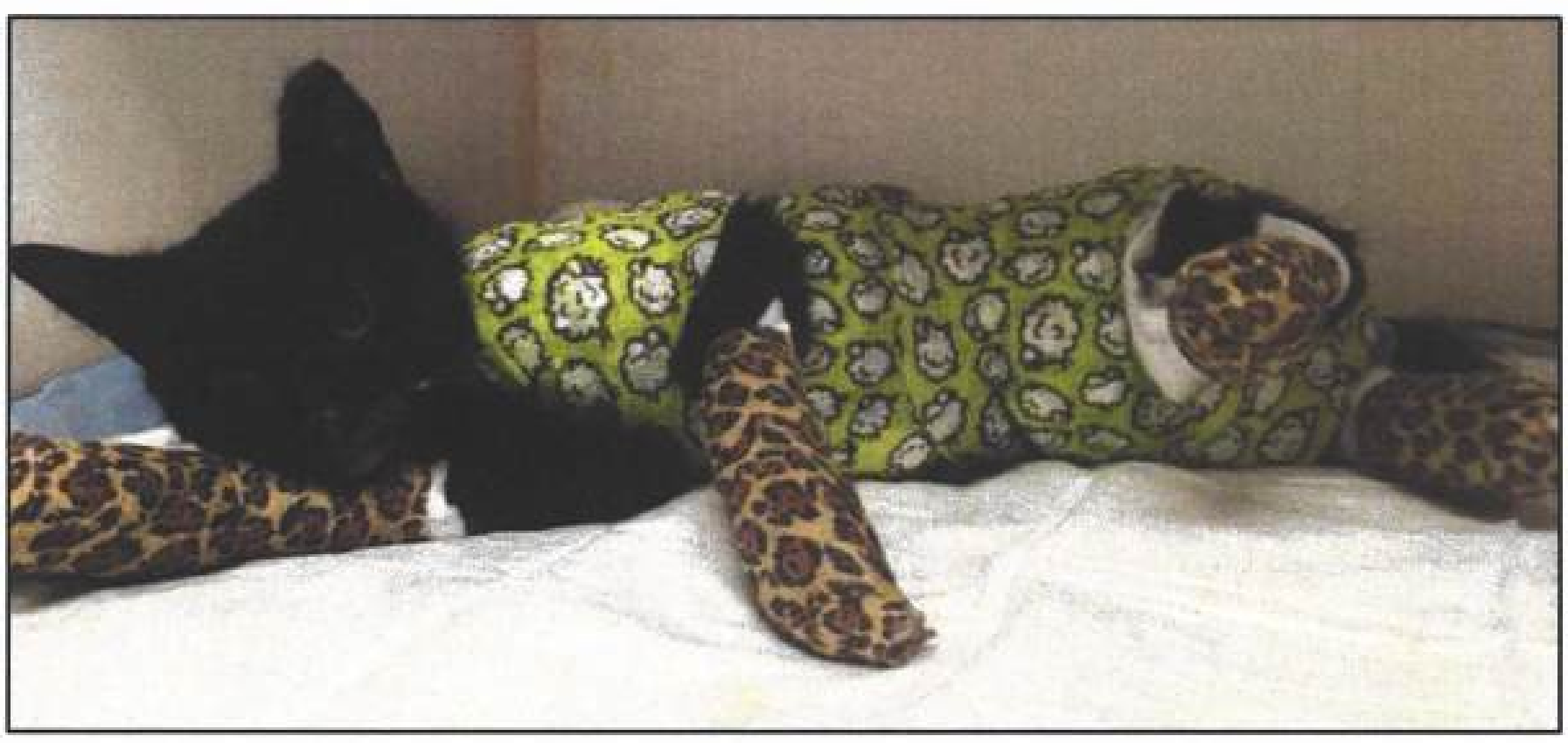

*Dierenbeul stopt kat Sprotje een kwartier in een hete oven.*

# Inzetcriteria FODO

## - Mishandeling dier Art 2.1 WD

- Pijn of letsel veroorzaken bij alle dieren->OA Kop-staart onderzoek: Letsels fotografisch vastleggen met maatvoering met deskundige dierenarts en formulier forensisch dieronderzoek door dierenarts laten invullen.
  PV van bevindingen opmaken en forensisch dossier maken.

- Diergevechten: gezondheid en welzijn van het dier benadelen -> OA PD onderzoek en veiligstellen van attributen die diergevechten kunnen aantonen. Humaan en Niet humaan biologisch celmateriaal veiligstellen op de PD. Kop-staart onderzoek. Wangslijmvlies van hond veiligstellen voor vergelijking.

## Doden van dieren art. 2.10 WD

- Doden van honden, katten en ganzen-> OA. Dier SIN geven en op juiste wijze verpakken Sporen van dier veiligstellen, zoals tie-wrap, tape e.d. Toestemming Sectie aanvragen bij OVJ en veterinair patholoog. Dier laten vervoeren, Aanvraag benoeming deskundige opmaken met vraagstelling onderzoeksvragen. PV van bevindingen opmaken. PD/omgevingsonderzoek.
- Doden van andere dieren, niet op juiste wijze-> OA Dier SIN geven en op juiste wijze verpakken. Casus voorleggen aan OVJ. Is sectie noodzakelijk?

## Onthouden van nodige zorg art 2.2 lid 8 WD

- Verwaarlozing van een dier. Als houders/ eigenaren niet met ziek/gewond dier naar dierenarts gaan. Het dier onthouden van water en voeding. Het dier onjuiste voeding geven. Indien dier is overleden: Dier SIN geven en op juiste wijze verpakken. Toestemming Sectie aanvragen bij OVJ en veterinair patholoog. Dier laten vervoeren, Aanvraag benoeming deskundige opmaken met vraagstelling onderzoeksvragen. PV van bevindingen opmaken. PD/omgevingsonderzoek.

**Bestialiteit:** Kop- staart onderzoek dier en het eventueel het vast leggen van eventuele letsels. Het bemonsteren vagina/penis van het dier door dierenarts. Insturen van DNA sporen. PD onderzoek waar bestialiteit heeft plaatsgevonden.

**Hondenhandel/puppyhandel:**
Doorzoekingen woningen. Ondersteunen team met fotografische opnamen en het veiligstellen van sporen/sporendragers. Onderzoek van dierenpaspoorten, medicatie, chipreaders.
Maken van een PV-doorzoeking.

**Verwantschapsonderzoek:** wangslijmvlies nemen van dieren om aan te tonen of ze verwant zijn met elkaar.

**Bijtincidenten**: Bemonsteren van speeksel van de bijtende hond vanaf het slachtoffer hond. Vastleggen eventuele letsels. (Opzet moet kunnen worden aangetoond)

**Dumpen van (levende) dieren art. 2.1 lid 3 WD:** PD onderzoek; bemonsteren van achtergelaten sporendragers door de eigenaar/ houder van het dier. Bijvoorbeeld hengsel van een tas, bench, blikjes, halsband van hond.

# Verpakken van een kadaver

Een kadaver(*) wordt verpakt in een breathable bag of een kinderlijkzak.
Indien het dier in een breathable bag past, dan gelieve deze te gebruiken. Dit is voor chain of evidence voldoende.
*In dit handelingskader is geen beschermende kleding opgenomen, gezien de doelgroep waar deze voor is bestemd.*

❖ **Breathable bag/ademende zak**:

- Maak foto's van het kadaver en de eventuele zichtbare letsels in detail, met een maatlat.
- Bemonster eventueel plaatsen op de vacht met een stub of swab
- Verwijder eventuele touwtjes, tape, tie-wrap e.d. die op het lichaam van het kadaver zitten. Stel deze sporendragers apart veilig.
- Geef het dier een SIN en label dit om een poot.
- Verpak het dier in een steriel laken.
- Verpak hierna het dier in een passende breathable bag.
- Plak de overige stickers op de breathable bag.
- Sluit de breathable bag af met anti fraude plakstrip.
- Plaats de zak met het dier in de koeling (**) Let op!! **NIET INVRIEZEN**

❖ **Lijkzak**

- Maak foto's van het kadaver en de eventuele zichtbare letsels in detail met maatlat.
- Bemonster eventueel plaatsen op de vacht met een stub of swab.
- Verwijder eventuele touwtjes, tape, tie-wraps e.d. die op het lichaam van het kadaver zitten. Stel deze sporendragers apart veilig.
- Geef het dier een SIN en label dit om een poot.
- Verpak het dier in een steriel laken.
- Verpak het dier in de lijkzak.
- Sluit de lijkzak af met een label door de ritsen en NFI sluitzegel. (zoals bij stoffelijk overschot)
- Plak de overige stickers op de lijkzak.
- Plaats de lijkzak met het dier in de koeling(**). Let op!! **NIET INVRIEZEN**

*(*) Een kadaver die in aanmerking komt voor sectie is en dier dat door menselijk toedoen, in het kader van Wet Dieren art. 2.10 lid 1of 2 i.v.m. 8.11 lid 1, om het leven is gekomen/gebracht. (zie wetgeving)*
*(**)Leg het dier in een koeling. Dit kan bij de dierenambulance of dierenkliniek.*
***In BVH vastleggen waar het kadaver ligt!*** *(zie netwerkkaart)*

# Stappenplan na het veiligstellen van het Kadaver

Vaak is er reeds contact geweest met collega's uit het basisteam met taakaccent DCG. Zij zijn zaaks verantwoordelijk en zullen het onderzoek verder oppakken.
De rol van de Forensische Opsporing is adviserend in de mogelijkheden en handelend op het gebied van het veiligstellen van sporen.

- Maak een proces-verbaal op van het eventueel bemonsteren en het veiligstellen van het kadaver.
- Zet in het proces-verbaal de ambtshandelingen die zijn verricht en redenen van wetenschap. Zet hierin ook de aanleiding van het onderzoek. Voeg ook een fotobijlage toe. De betrokken collega's zullen een proces-verbaal maken van het aantreffen van een dier, ook met een fotobijlage erbij.
- Neem contact op met de officier van justitie, taakaccent dieren. Dit kan telefonisch, per mail, of WhatsApp. (Zie netwerkkaart )
  Vraag hem goedkeuring voor een sectie.
- Neem contact op met Monique Verkerk via de WhatsApp of ze in de gelegenheid is om een sectie te verrichten. Zet hierin heel kort om wat voor dier het gaat en wat de toedracht is. (Zie netwerkkaart voor telefoonnummer Monique).
- Waarschijnlijk moet het kadaver worden vervoerd naar Purmerend of een andere onderzoek locatie. *Uiteraard mogen kadavers ook door de Politie of Forensische Opsporing worden vervoerd.*
- Indien Monique geen gelegenheid heeft, kan worden uitgeweken naar de Universiteit Utrecht- faculteit diergeneeskunde. (Zie netwerkkaart)
- Maak een aanvraag RC benoeming deskundige op en vermeld hierop de onderzoeksvragen. Mail deze naar de officier van Justitie én na benoeming aan Monique Verkerk. Het mag ook worden gemaild naar de collega die de zaak in behandeling heeft. (Zie hoofdstuk; Vraagstellingen in aanvraag benoeming deskundige)
- Een aanvraag NFI hoeft ***niet*** te worden opgemaakt.
- Het kadaver mag **na toestemming** van officier van Justitie, gezien de ontbinding, **vóór** de benoeming afgegeven is al naar onderzoeklocatie worden vervoerd.
- Na de sectie is het van belang dat het kadaver niet in de koeling (bij Monique) blijft liggen. Vraag daarom aan officier van Justitie toestemming voor vernietiging. Indien er een eigenaar bekend is, kan het kadaver in overleg ook aan hen worden teruggegeven. De eventuele kosten die hiermee zijn gemoeid, zijn wel voor de eigenaar.

# Voorbeelden vraagstelling proces-verbaal aanvraag benoeming deskundige

## Wanneer kan een sectie op dieren worden aangevraagd?

In gevallen waarbij een dier is overleden door opzettelijk handelen van mensen zoals omschreven in de Wet Dieren en in het Besluit houders van dieren.

## Aanvraag benoeming deskundige

Beschrijf in het proces-verbaal het incident uitvoerig. Beschrijf kort welk strafbaar feit er is gepleegd. (Zie wetgeving en bevoegdheden) Vermeld in de motivering ook het strafbare feit, zoals bijvoorbeeld; *"Gezien artikel 2.1 van de Wet Dieren, dat het verbiedt bij een dier zonder redelijk doel pijn of letsel te veroorzaken, dan wel de gezondheid of het welzijn te benadelen, dient dit spoor/deze sporendragers te worden onderzocht."*
Stel de vraagstellingen op, zie hieronder voorbeelden.
Dit proces-verbaal wordt ook gemaild naar de uitvoerende patholoog. Aan de hand van de vraagstelling, gesteld in dit proces-verbaal, onderzoekt zij het kadaver.
(LET OP: **Voeg het Curriculum Vitae van Monique Verkerk toe bij een aanvraag benoeming deskundige, indien zij sectie gaat verrichten.**)

### Aanvraag benoeming deskundige: Vraagstelling

Hieronder staan vragen die aan de patholoog kunnen worden gesteld in het kader van de bewijsvoering zoals omschreven in de Wet Dieren, door middel van een aanvraag benoeming deskundige. Het is natuurlijk vrij, andere relevante onderzoek vraagstellingen te gebruiken.

SIN ……… : - VETERINAIRE PATHOLOGIE/RADIOLOGIE/ANTROPOLOGIE-

- Wat is de doodsoorzaak? (is een hele legitieme vraag, behalve bij euthanasie)
- Kunt u iets zeggen over de algehele conditie en leeftijd van de(diersoort)?
- Kunt u iets zeggen over de letsels en of er reeds letsel was voor het incident waarbij het dier is overleden?
- Kunt u iets zeggen over de krachtsinwerking? Zijn de letsels bijvoorbeeld incidenteel ontstaan tijdens het incident of niet-incidenteel ontstaan, door (stelselmatige) mishandeling?
- Zijn er oude letsels aanwezig is, kunt u iets zeggen over wanneer deze letsels ongeveer bij leven zijn ontstaan?
- Zijn er ziekelijke afwijkingen, die de dood kunnen verklaren?

### Aanvraag opdracht en benoeming deskundige

Gezien het bovenstaande wordt ten behoeve van de waarheidsvinding aan de officier van justitie verzocht om een opdracht en benoeming te vorderen van de rechter-commissaris bij de Rechtbank … voor een deskundige van Stichting forensisch dierenonderzoek, mevrouw drs. Monique Verkerk op het gebied van Forensische pathologie.
Deze deskundige wordt aan de hand van het genoemde stuk van overtuiging opgedragen bovenstaande onderzoeksopdracht uit te voeren.

## Sectie op een kadaver

Voorheen werd vaak de Universiteit Utrecht –faculteit diergeneeskunde gevraagd een sectie uit te voeren. Dit bracht veel kosten met zich mee, de rapportage liet lang op zich wachten en het rapport was in (voor een leek onleesbare) medische termen geschreven.
De UU is heel goed in het onderzoeken van de ziekelijke afwijkingen. Wat wij juist willen onderzoeken is het strafrechtelijke deel. Kan er aan de bevindingen van een patholoog een strafbaar feit worden gekoppeld.
Vanuit de Landelijke werkgroep DCG werd dierenarts Drs. Monique Verkerk aanbevolen, mede door haar snelle inzet en leesbare rapportages.
Doordat ze zichzelf heeft bewezen en landelijk haar expertises vaak worden ingezet, heeft ze het drukker gekregen waardoor ze niet alle secties kan uitvoeren.

Zoek daarom via een WhatsApp bericht contact met Monique of ze in de gelegenheid is een sectie uit te voeren. De ervaring is dat ze snel een antwoord terug stuurt. Indien ze geen tijd heeft zal moeten worden uitgeweken naar de UU-diergeneeskunde.

Drs Monique Verkerk
www.forensischdieronderzoek.nl
0622542752
*Alleen* huisdieren

| | |
|---|---|
| *Huisdieren en landbouwdieren* | Faculteit diergeneeskunde Utrecht<br>www.uu.nl<br>030-2533195<br>r.wagensveld@uu.nl |
| *Wilde dieren* | Peter van Tulden, Universiteit Wageningen https://www.wur.nl/nl<br>peter.vantulden@wur.nl |

## Voorbeeld rapportage Drs. Monique Verkerk naar aanleiding van BVH 2022203089 Eenheid Rotterdam

**Inwendig**

Het dier werd geopend via een klassieke snede met preparatie van de vacht. Er was vrijwel geen vetweefsel en minimaal spierweefsel. De weefsels voelden droog en plakkerig aan. Op de buikzijde bij de staartaanzet was een kleine bloeding zichtbaar in het spierweefsel. Geen bloeduitstortingen of tekenen van trauma op de romp en ledematen. Na openen buikholte normale anatomie van de organen. Geen vrij bloed in de buikholte. Lever, milt en nieren iets bleek, verder geen afwijkingen.

Blaas was leeg. Na openen van de maag bleek deze geen voedselbrij te bevatten maar donker gekleurde corpora aliena (voorwerp dat in een lichaam wordt aangetroffen maar daar oorspronkelijk niet thuishoort) vermengd met wat slijm. De stukjes corpora aliena leken op stukjes hout. Ook in de darm bevonden zich geen voedselresten of ontlasting maar eenzelfde soort corpora aliena. Verder waren de darmen leeg. Inwendig geen vetweefsel aantoonbaar. Na openen borstholte normale anatomie van de borstorganen. Geen afwijkingen aan hart of longen, behalve ook hier de organen te bleek. Geen vrij bloed in de borstholte. Het middenrif intact. Geen oude fracturen voelbaar en zichtbaar op de ribben. Na openen luchtpijp bloed en stolsels aantoonbaar in de gehele luchtpijp.

Over de gehele rugzijde van de romp geen bloeduitstortingen of fracturen waarneembaar. Na prepareren van de vacht van de kop uitgebreide bloedingen en stolsels waarneembaar. De schedel was meervoudig gefractureerd met uitpuilen van hersenweefsel onder de huid. Het hersenvlies was volledig weggescheurd. Er lagen losse botfragmenten van de schedel tussen het hersenweefsel.

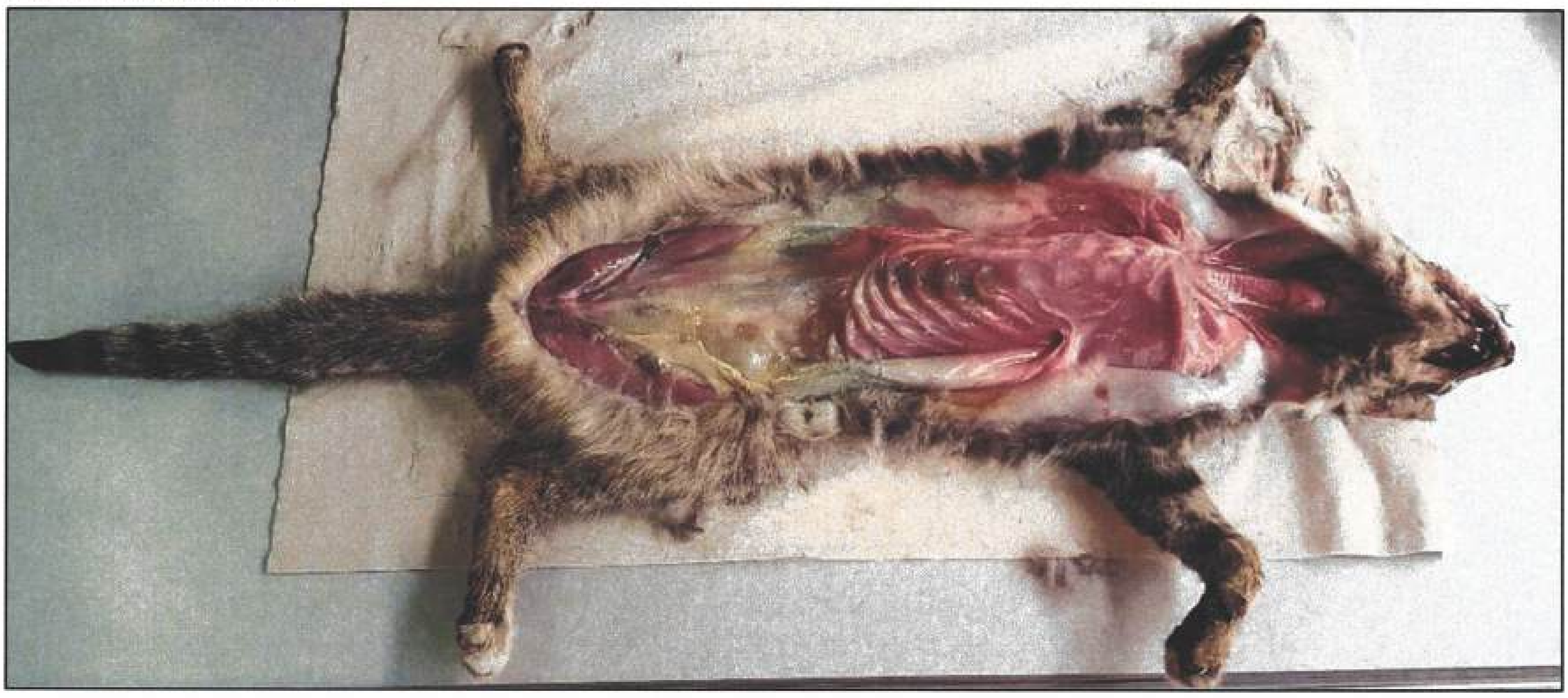

**Samenvatting**

A: Uitwendig

1. Er was geen chip aanwezig
2. Het dier was extreem mager en uitgedroogd
3. De kop was vervormd met uitgebreide bloedingen en fracturen in het kop gebied.
4. Het linkeroog puilde uit de oogkas, het rechteroog was diep in de oogkas verzonken

B: Inwendig

1. Er waren meervoudige fracturen in de schedel en onderkaak
2. Er waren gebroken hoektanden
3. Er zat geen voedselbrij maar corpora aliena in maag en darmen

C: Algemeen
1. Fotodocumentatie werd vervaardigd.
2. Monsters van maaginhoud en darminhoud werden veiliggesteld
3. Na onderzoek werd het dier gesloten.
4. In overleg met de opdrachtgever wordt het dier in de afgesloten koeling van werkplek Purmerend bewaard.

Teneinde de doodsoorzaak aan te wijzen en ter duiding van al wat meer van belang zou mogen blijken, werd om forensisch dierenonderzoek verzocht. Het dier werd aangeboden bij de onderzoeklocatie te Purmerend en veiliggesteld tot onderzoek.
Specifieke vragen waren:
1. Wat is de doodsoorzaak?
2. Wat is de algehele conditie en de leeftijd van de kat?
3. Wat is er te zeggen over de letsels/was er reeds letsel voor de aanrijding met het voertuig?
4. Wat is er te zeggen over de krachtsinwerking? Zijn letsels bijvoorbeeld incidenteel ontstaan door de aanrijding of niet-incidenteel ontstaan, door (stelselmatige) mishandeling?
5. Als er oud letsel bij de kat aanwezig is, is er iets te zeggen over wanneer bij leven deze is ontstaan?
6. Zijn er ziekelijke afwijkingen, die de dood van de kat kunnen verklaren?

Conclusie
Bij sectie werd een (jong)volwassen vrouwelijke intacte kat gezien. De conditie was niet goed, het dier was extreem mager. Het gewicht was 1.35 kg, dit zou een normaal gewicht moeten zijn van een kitten van rond de 10-11 weken (zie tabel 1 pagina 20). Echter, het gebit was uitgewisseld en dit is bij katten van ongeveer 7 maanden het geval. Een kat van dit formaat en leeftijd zou minimaal het dubbele moeten wegen. Het lage gewicht bleek ook uit de matige bespiering, het vrijwel niet aantoonbaar zijn van vetweefsel en de uitdroging. Opvallend was de inhoud van maag en darmen. Er was geen voedselbrij aanwezig maar wel corpora aliena wat op stukjes hout en plantendelen leek.
Dit zou betekend kunnen hebben dat de kat onvoldoende voedsel tot haar beschikking heeft gehad. We zien nogal eens dat dieren die geen of te weinig voedsel krijgen, pica gedrag vertonen. Dit betekent dat, wanneer een dier niet dagelijks voldoende eten binnen krijgt, het niet-eetbare dingen gaat eten zoals steentjes, modder, hout of aarde. Als de kat al agressief zou zijn geweest, zou het kunnen dat het feit dat ze onvoldoende voedsel tot haar beschikking heeft gehad, een reden was om afwijkend gedrag te vertonen. Er was tijdens onderzoek geen medische reden gevonden dat ze niet normaal voedsel zou kunnen eten.
Er waren geen oude letsels zichtbaar. De ribben en ledematen waren zowel op de röntgenfoto's als bij sectie intact zonder oude (genezen) fracturen. Ook de organen oogden normaal zonder oud letsel.
Er is geen reden om aan te nemen dat dit dier stelselmatig werd mishandeld.
De doodsoorzaak is het trauma aan de kop. Zowel de schedel als de onderkaak was meervoudig gefractureerd waarbij botfragmenten los lagen. De schedel was zo beschadigd dat er massale bloedingen zijn opgetreden en het bloed uit bek, neus en oren kwam. Ook de luchtpijp was gevuld met bloed en stolsels. Het hersenweefsel was uit de schedel gekomen en lag los onder de huid. De ogen zijn door het trauma uit de oogkassen los gekomen. Deze verwondingen waren het gevolg van heftig uitwendig botsend mechanisch geweld zoals kan voorkomen bij het met kracht met de kop ergens tegen aan slaan. De verwondingen kunnen zeer goed passen bij het tegen een auto gooien van een levend dier. Gezien de massale bloeduitstortingen en aanwezige stolsels is dit bij leven opgelopen. Het verklaart het intreden

van de dood zondermeer op basis van uitval van neurogene functies. Er waren geen ziekelijke afwijkingen aantoonbaar.

Richtwaarde gewichtstabel kittens.

| **Week** | **Poes/gram** |
|---|---|
| 8 | 890 |
| 9 | 980 |
| 10 | 1260 |
| 11 | 1520 |
| 12 | 1680 |

Dit rapport is naar waarheid, volledig en naar beste inzicht opgemaakt en getekend,

Drs. M. Verkerk, MSc. Veterinair Forensisch Arts

# Forensisch onderzoek levende dieren

We zullen bij de Forensische Opsporing ook te maken krijgen met levende dieren.

**Puppyhandel**

Het kan het voorkomen dat er bijvoorbeeld verwantschap onderzoek wordt ingezet, in zaken waarbij bijvoorbeeld hondenhandelaren zijn betrokken.

In Nederland is de illegale puppyhandel een groot probleem, zeker in tijden van de Corona pandemie. Puppy's worden uit het buitenland gehaald. Ze zijn vaak ziek en worden in Nederland voor heel veel geld verkocht. De dieren worden via Marktplaats of Facebook aangeboden. De moederhond leeft onder erbarmelijke omstandigheden in landen zoals Roemenië, Polen en Bulgarije en heeft vaak nest op nest. Vaak zijn de puppy's niet ingeënt, niet gechipt en hebben een vervalst paspoort. Het risico van het kopen van een jong dier uit het buitenland is, dat ze ziek zijn en lijden aan ziekten zoals Parvo, schurft, Giardia en Zoönose (op mensen overdraagbare ziekten) en deze mee naar Nederland nemen waar onze huisdieren en ook wij zelf ziek van kunnen worden. Het kan voorkomen dat de forensische opsporing ook wordt gevraagd bij doorzoekingen van woningen van deze puppyhandelaren aanwezig te zijn. In dat geval is gewenst fotografische opnamen te maken van de toestand van de dieren. Ook het hebben van bepaalde medicatie en chips/inbrengnaalden is verboden. Dit moet in beslag worden genomen. Neem ook de chipreaders in beslag voor het uitlezen van de gegevens.

*Voorhanden hebben (UDD)diergeneesmiddelen:*
*Antibiotica zijn UDD middelen en die mogen alleen door dierenartsen zelf toegediend worden; dus als iemand (niet zijnde dierenarts) deze middelen voorhanden heeft, gebruikt deze ze niet in overeenstemming met de vergunning*
*Art. 2.20 Wet dieren*
*1. Bij of krachtens algemene maatregel van bestuur worden regels gesteld voor de uitvoering van bindende onderdelen van EU-rechtshandelingen met **betrekking tot diergeneesmiddelen** of gemedicineerde diervoeders.*
*2. Bij of krachtens algemene maatregel van bestuur kunnen voor het onderwerp, bedoeld in het eerste lid, regels worden gesteld die betrekking hebben op onder meer:*
*b. de kleinhandel in diergeneesmiddelen; (dierenartsen hebben namelijk van rechtswege een vergunning voor kleinhandel, 4.3 Besluit diergeneesmiddelen 2022)*

*Noot: Er zijn ook verschillende rescue organisaties die straatdieren uit het buitenland naar Nederland halen voor een beter leven. Bij deze organisaties is uitbuiting van dieren niet aan de orde en honden worden eerst door een dierenarts gezien voordat ze naar Nederland vertrekken. Bij deze dieren zal er een contract moeten worden getekend.*

## Dierenmishandeling/onthouden van nodige zorg

Maar ook zeker als er sprake is van diermishandeling kan de forensische opsporing iets betekenen. Bij mensen wordt een top-teen onderzoek uitgevoerd. Bij dieren heet dit een kop-staart onderzoek en kan zeker van meerwaarde zijn in de rechtszaak en het opleggen van een houdverbod. Een kop-staart onderzoek wordt uitgevoerd door een dierenarts die de forensische module heeft gevolgd bij de Universiteit Utrecht. Tijdens het onderzoek worden er fotografische opnamen gemaakt van de letsels, met maataanduiding. Hierbij wordt het formulier Forensisch onderzoek levende dieren gebruikt. Dit formulier is als bijlage gevoegd bij dit handelingskader en staat op de agorasite van DCG.
In dit formulier wordt ook de algehele conditie van het dier beschreven.

Uit een eerste indruk kunnen we de toestand van het dier concluderen:

- Bewustzijnsniveau (alert, sloom, apathisch)
- Gedrag (angst, stress, agressief, herhaald gedrag- steeds rondjes lopen)
- Houding en gang (hoofd/kop laag holle rug, kreupel)
- Voedingstoestand (body condition scores) #
- Verzorgingstoestand (hoeven, nagels, tanden en vacht)
- In het oog springende klinische afwijkingen (geen staart, mist poot, veel te dik, slechte omgeving)

#Een body condition score is een aanduiding voor de conditie van het dier. Vraag welke condition score de dierenarts hanteert, bijvoorbeeld, body condition score 3 van 1 tot 6. 3 is dan een gemiddelde score, 1 geeft aan dat het dier heel mager is en 6 geeft aan dat het dier overgewicht heeft.

In de onderstaande gevallen zou de Forensische Opsporing met levende dieren te maken kunnen krijgen:

- Dierenmishandeling, verwaarlozing, onthouden van nodige zorg
- Verwantschap onderzoek tussen dieren is mogelijk en wordt uitgevoerd door het NFI. Het onderzoek kan op basis van wangslijmvlies, bloed en haren (met wortel). Niet humaan biologische sporen (NHBS201-maatwerk)
- Bij bijtincidenten het slachtoffer (mens of dier) en hond bemonsteren op biologisch celmateriaal zodat bijtincident kan worden bewezen.
- Gedumpte dieren waar diverse sporendragers (riem, touw, transport bench e.d.) door de eigenaar zijn achter gelaten.
- Dieren die zijn gekneveld met tape, touw of tie-wrap.
- Dieren die zijn geschoten. (dartpijl door snavel van eend)
- Bestialiteit
- Vergiftiging

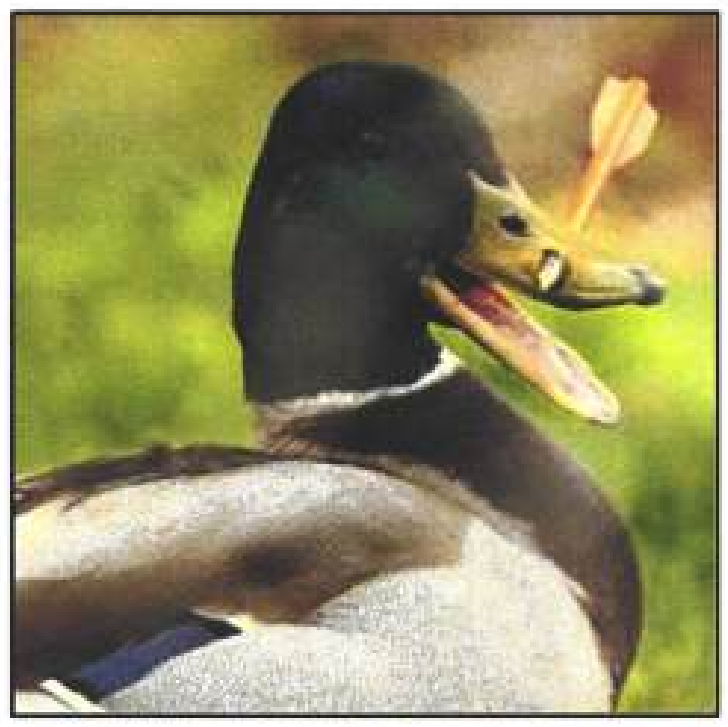

De (Forensisch) Dierenarts zal het dier onderzoeken en het touw, de pijlen, het tape en de tie-wrap verwijderen. Tijdens het onderzoek worden sporen en sporendragers veiliggesteld. In ernstige zaken is het wenselijk dat de Forensische Opsporing en de dierenarts samen het onderzoek verrichten en de sporen veilig stellen. De Forensische Opsporing kan de dierenarts adviseren over de wijze waarop sporen kunnen worden veiliggesteld zodat er geen sporen verloren gaan. Net als bij mensen gaat hulpverlening natuurlijk altijd voor.
Laat de dierenarts het onderzoeksverslag formulier invullen. (zie bijlage)

## Dierenartsen

Dierenartsen zijn zeker bereid om te helpen. Ze hebben een specialisme zoals, dierenartsen voor gezelschapsdieren, bijzondere dieren zoals kleine knagers, slangen, vogels e.d. landbouwdieren en paarden/ezels.

Voordat er een dierenarts wordt ingeschakeld, is het wel belangrijk om te weten wie de opdrachtgever in de zaak is, zodat de kosten door de dierenarts kunnen worden gedeclareerd. Dit zal vaak de officier van Justitie zijn. Geef daarom een visite kaartje met jouw contactgegevens, de naam van de officier van Justitie en het BVH-nummer.
Het is vaak voor dierenartsen lastig, de gemaakte kosten op de eigenaar te verhalen omdat deze geen klant is of financiële problemen heeft.
De beslagkosten zijn voor het Openbaar Ministerie en de **onderzoekskosten** en vervoerskosten zijn voor de **Nederlandse Politie.**
Vraag bij de dierenarts na of de facturering is gelukt. Dat schept vertrouwen en zo kan je een netwerk opbouwen.

1-4-4 RED EEN DIER

## Meldcode dierenartsen

Een dierenarts kan bij vermoeden van diermishandeling het Landelijk expertisecentrum dierenmishandeling (LED) inschakelen. Dit valt onder de beroepsvereniging dierenartsen Nederland, het Koninklijke Nederlandse Maatschappij voor diergeneeskunde (KNMvD). Burgers kunnen hier *geen* meldingdoen van het vermoeden van mishandeling. Burgers kunnen daarvoor nummer 144 bellen.

**Doel van de meldcode**
De meldcode heeft als doel dierenartsen te helpen met het signaleren van dierenmishandeling. Daarnaast wil de KNMvD met deze meldcode dierenartsen ondersteunen bij het maken van een goede afweging bij het melden van deze dierenmishandeling. De meldcode wijst dierenartsen de weg naar instanties die hen kunnen adviseren én waar men melding kan doen. Uiteindelijk moet dit leiden tot snellere detectie en voorkómen van dierenmishandeling.

**Beroepsgeheim**
In de wet is geen beroepsgeheim voor de dierenarts opgenomen. Traditioneel behoren geestelijken, artsen, notarissen en advocaten tot de beroepen met een beroepsgeheim. Wanneer er geen wettelijke bepaling is, kan een beroepsgeheim echter ook voortvloeien uit bijvoorbeeld een overeenkomst, een maatschappelijke norm of uit beroepscodes. In de code voor de dierenarts van de KNMvD is een artikel opgenomen over de geheimhouding van patiënt- en cliëntgebonden gegevens.
Wat is een beroepsgeheim? Het beroepsgeheim geeft vorm aan het al door Hippocrates gehuldigde algemeen rechtsbeginsel dat eenieder zich vrij en zonder vrees voor openbaarmaking van bepaalde informatie tot een dienstverlener moet kunnen wenden. Beroepsgeheim is geen absoluut recht. Natuurlijk dient men terughoudend te zijn met het doorbreken van het beroepsgeheim, maar zwaarwegende (maatschappelijke) belangen kunnen aanleiding zijn het beroepsgeheim te doorbreken. Dit kan na toestemming van de betrokkene, op grond van een wettelijke bepaling en in bepaalde gevallen van noodtoestand of overmacht.

**Schenden van beroepsgeheim**
Normaal kan als het beroepsgeheim wordt geschonden op grond van artikel 272 van het Wetboek van Strafrecht een strafvervolging plaatsvinden en eventueel een tuchtrechtelijke procedure worden gestart. Wanneer een dierenarts echter bij het volgen van deze, door de overheid gestimuleerde, meldcode voor dierenmishandeling een melding doet, is er geen sprake van schending van een beroepsgeheim.

**Toestemming voor melding**
Als de situatie zich voordoet dat toestemming vragen aan de diereigenaar mogelijk is, is dat wenselijk. Een voorbeeld van een dergelijke situatie is als er een ander gezinslid, dan op dat moment aanwezig, betrokken is bij de dierenmishandeling. Maar zelfs als er geen goedkeuring is, óf als een gesprek over de geplande melding om veiligheidsredenen onmogelijk is, kan een dierenarts dierenmishandeling melden.

Zie ook: Meldcode dierenmishandeling voor dierenartsen – KNMvD –

## WETGEVING EN BEVOEGDHEDEN

Dierenwelzijn is wettelijk gezien een lastige materie omdat het onder zoveel verschillende wet en regelgeving valt. De algemene plaatselijke verordeningen (APV), strafrecht, strafvordering, wet economische delicten (WED), algemene bestuur wet, visserijwet, wet op de natuurbescherming en de Wet Dieren.

De Wet Dieren is een kaderwetgeving of een zogenaamde raamwet. Dit betekend dat er onder deze wet diverse besluiten en regelingen hangen. Hoewel er op de meeste misdrijven in de Wet Dieren een straf van minder dan 4 jaar wordt gesteld, zijn er in artikel 67 1c Wetboek van Strafvordering artikelen uit de Wet Dieren met naam genoemd en zijn feiten waar voorlopige hechtenis wordt toegestaan.

# recent heeft de kamer ingestemd met een wetwijziging, waarbij de strafmaat op sommige artikelen van de WD van drie naar vijf jaar wordt verhoogd. Dit is een zogenaamde hamerwet en kan snel in gaan. Wanneer deze wet in gaat is nog niet bekend.

In de Wet Dieren (1.3) worden de intrinsieke waarde van een dier benoemd volgens de **5 vrijheden van Brambell.**

1) Vrij van honger en dorst en onjuiste voeding
2) Vrij van fysiek en fysiologisch ongerief
3) Vrij van pijn verwondingen en ziekten
4) Vrij van angst en chronische stress
5) Vrij om natuurlijk gedrag te vertonen

### Wat is dierenmishandeling?

Artikel 2.1 lid 1
2.1 lid 2, a,b,c,d
2.1 lid 3
- 1.3 besluit houders voor dieren

2.1 lid 6

### Voor welke dieren?

Gehouden dieren en andere dan gehouden dieren
(Huisdieren, landbouwdieren en wilde dieren. Uitgezonderd zijn de dieren die leven in de Oostvaardersplassen. De rechter heeft hier een uitspraak over gedaan.)

### Artikel 2.1. Dierenmishandeling WD

1. Het is verboden om zonder redelijk doel of met overschrijding van wat ter bereiking van zodanig doel toelaatbaar is, bij een dier pijn of letsel te veroorzaken dan wel de gezondheid of het welzijn van het dier te benadelen.
2. Tot de in het eerste lid verboden gedragingen worden in ieder geval gerekend:
   - **a.** een dier arbeid doen verrichten die kennelijk zijn krachten te boven gaat of waartoe het uit hoofde van zijn toestand ongeschikt is;
   - **b.** een koe met overvolle uier vervoeren of op een markt of openbare verkoping ten verkoop houden;
   - **c.** bij de verlossing van een koe gebruikmaken van dierlijke trekkracht of van een niet daarvoor toegelaten krachttoestel, en
   - **d.** een hond als trekkracht gebruiken met uitzondering van de sledehondensport, voor zover toegelaten.

3. Bij algemene maatregel van bestuur kunnen voorts gedragingen worden aangewezen die in ieder geval worden gerekend tot de verboden gedragingen, bedoeld in het eerste lid.
4. Bij ministeriële regeling kunnen regels worden gesteld voor een toelating als bedoeld in het tweede lid, onderdeel c en d, of voor de bij algemene maatregel van bestuur, bedoeld in het derde lid, aangewezen gedragingen.
5. Van de krachtens het derde lid aangewezen gedragingen kan, al dan niet in daarbij aangewezen gevallen, deel uitmaken het gebruik van voorwerpen die bij dieren pijn of letsel kunnen veroorzaken, dan wel de gezondheid of het welzijn kunnen benadelen.
6. Een ieder verleent een hulpbehoevend dier de nodige zorg.
7. Het bij en krachtens het eerste tot en met het zesde lid bepaalde is tevens van toepassing ten aanzien van andere dan gehouden dieren.

## Artikel 8.11 WD strafbaarstelling

1. Gedragingen in strijd met de voorschriften vastgesteld bij of krachtens de artikelen 1.4, 2.1, eerste lid, 2.2, zesde lid, eerste volzin, en achtste lid, 2.8, eerste lid, onderdeel a, derde en vierde lid voor wat betreft de onderwerpen, bedoeld in het vierde lid, onderdelen a, b en c, 2.9, eerste lid, 2.10, eerste lid, en 2.14, eerste lid, of een of meer van de voornoemde bepalingen in samenhang met artikel 6.2, eerste lid, artikel 6.4, eerste lid, of artikel 7.5, derde lid, zijn misdrijven.
2. Gedragingen in strijd met de voorschriften vastgesteld bij of krachtens de artikelen 2.1, zesde lid, 2.2, eerste lid, negende en tiende lid voor wat betreft de onderwerpen, bedoeld in het tiende lid, onderdelen b, c en d, en onderdeel r, voor zover dat onderdeel betrekking heeft op regels als bedoeld in de onderdelen b, c en d, 2.3, derde en vierde lid, 2.4, eerste, tweede en derde lid, 2.8, derde en vierde lid voor wat betreft de onderwerpen, bedoeld in het vierde lid, onderdelen d en e, 2.9, tweede, vierde en vijfde lid, 2.14, tweede lid, 2.15, eerste, tweede, derde, vijfde en zesde lid, en 4.4, eerste lid, of een of meer van de voornoemde bepalingen in samenhang met artikel 6.2, eerste lid, artikel 6.4, eerste lid, of artikel 7.5, derde lid, zijn overtredingen.

## Artikel 8.12 WD Strafmaat

1. Gedragingen in strijd met de voorschriften vastgesteld bij of krachtens de artikelen 2.1 eerste lid, 2.2, zesde lid, eerste volzin, en achtste lid, 2.10, eerste lid en 2.14, eerste lid, of een of meer van de voornoemde bepalingen in samenhang met artikel 6.2, eerste lid, artikel 6.4, eerste lid, of artikel 7.5, derde lid, worden gestraft met gevangenisstraf van ten hoogste drie jaren of een geldboete van de vierde categorie.
2. Gedragingen in strijd met de voorschriften vastgesteld bij of krachtens de artikelen 2.8, eerste lid, onderdeel a, en derde en vierde lid voor wat betreft de onderwerpen, bedoeld in het vierde lid, onderdelen a, b en c, of een of meer van de voornoemde bepalingen in samenhang met artikel 6.2, eerste lid, artikel 6.4, eerste lid, of artikel 7.5, derde lid, voor zover deze gedragingen plaatsvinden anders dan in de uitoefening van een bedrijf waar dieren van krachtens artikel 2.3, tweede lid, aangewezen soorten of categorieën, worden gehouden, worden gestraft met gevangenisstraf van ten hoogste drie jaren of een geldboete van de vierde categorie.
3. Gedragingen in strijd met de voorschriften vastgesteld bij of krachtens de artikelen 1.4, 2.1, zesde lid, 2.3, derde en vierde lid, 2.8, derde en vierde lid voor wat betreft de onderwerpen bedoeld in het vierde lid, onderdelen d en e, 2.9, eerste, tweede, vierde en vijfde lid, 2.14, tweede lid, 2.15, eerste, tweede, derde, vijfde en zesde lid, en 4.4, eerste lid, of een of meer van de voornoemde bepalingen in samenhang met artikel 6.2, eerste lid, artikel 6.4, eerste lid, of artikel 7.5, derde lid, worden gestraft met een hechtenis van ten hoogste zes maanden of een geldboete van de derde categorie.

4. Gedragingen in strijd met de voorschriften vastgesteld bij of krachtens artikel 2.2, eerste lid, negende en tiende lid voor wat betreft de onderwerpen, bedoeld in het tiende lid, onderdelen b, c en d, en 2.4, eerste, tweede en derde lid, of een of meer van de voornoemde bepalingen in samenhang met artikel 6.2, eerste lid, artikel 6.4, eerste lid, of artikel 7.5, derde lid, voor zover deze gedragingen plaatsvinden anders dan in de uitoefening van een bedrijf waar dieren van krachtens artikel 2.3, tweede lid, aangewezen soorten of categorieën, worden gehouden, worden gestraft met een hechtenis van ten hoogste zes maanden of een geldboete van de derde categorie.
5. Indien gedragingen in strijd met de voorschriften vastgesteld bij of krachtens artikel 2.15, derde lid, worden gepleegd in verband met een paarden ren of harddraverij met betrekking tot welke een totalisator als bedoeld in artikel 23, tweede lid, van de Wet op de kansspelen is georganiseerd, wordt de in het derde lid voorziene hechtenis met een derde verhoogd.

**(volgens artikel 2.10 van Wet Dieren is het verboden Honden katten en ganzen te doden. Dit zijn aangewezen diersoorten* in het besluit houders voor dieren. De andere dieren mogen worden gedood, mits er juist wordt gehandeld. Zo mag een dier niet onnodig lijden en moet deze worden bedwelmd alvorens hij wordt gedood.)**

**Artikel 2.10 WD Doden van dieren**

1. Het is verboden om dieren behorend tot bij algemene maatregel van bestuur aangewezen diersoorten* of diercategorieën te doden, behoudens in gevallen waarin een dier wordt gedood voor de bedrijfsmatige productie van dierlijke producten of in bij of krachtens algemene maatregel van bestuur aangewezen gevallen.
2. Bij of krachtens algemene maatregel van bestuur worden regels gesteld voor de uitvoering van bindende onderdelen van EU-rechtshandelingen over het doden, het bedwelmen, het fixeren, het onderbrengen en het verplaatsen van dieren.
3. Bij of krachtens algemene maatregel van bestuur kunnen voor het onderwerp, bedoeld in het tweede lid, regels worden gesteld voor bij deze maatregel aan te wijzen diersoorten of diercategorieën die betrekking hebben op onder meer:

   a. een verbod op het doden van bepaalde dieren;

   b. de wijze waarop dieren worden gedood;

   c. situaties waarin het is toegestaan dieren te doden;

   d. voorwaarden waaronder het is toegestaan dieren te doden;

   e. de personen die dieren doden, of die daarbij betrokken zijn;

   f. de plaats waar dieren worden gedood;

   g. het vervoeren, het aanvoeren en het afvoeren van dieren naar de plaats waar wordt gedood;

   h. het verplaatsen van dieren in de ruimten waar dieren worden gedood;

   i. het onderbrengen van dieren in de ruimten waar dieren worden gedood;

   j. het fixeren van dieren;

k. het bedwelmen van dieren;

l. de inrichting, uitvoering en vormgeving van ruimten waar dieren worden gedood, waaronder de aanwezige voorzieningen;

m. de gegevens over de te doden dieren die voorafgaand aan het doden worden overgelegd;

n. de gezondheidsstatus van de te doden dieren;

o. de onderzoeken aan en met betrekking tot de dieren;

p. de keuring van dieren;

q. de personen die dieren keuren, of die daarbij betrokken zijn;

r. hygiëne, het voorkomen van de verspreiding van dierziekten, zoönose en ziekteverschijnselen, en het weren van ziekteverwekkers, en

s. de instrumenten, installaties en verdere voorzieningen voor het fixeren, bedwelmen of doden van dieren.

4. Het is toegestaan om dieren zonder voorafgaande bedwelming te doden volgens de israëlitische of de islamitische ritus. Bij algemene maatregel van bestuur worden in het belang van de bescherming van de dieren nadere regels gesteld over het doden volgens de israëlitische of de islamitische ritus.
5. De regels, bedoeld in het vierde lid, kunnen betrekking hebben op onder meer:
    a. de wijze waarop dieren worden gedood;
    b. de personen die het doden van dieren uitvoeren;
    c. de inrichting, uitvoering en vormgeving van ruimten waar dieren worden gedood, waaronder de aanwezige voorzieningen, en
    d. de aanwezigheid van een op grond van artikel 8.1 aangewezen ambtenaar, tevens zijnde een dierenarts, en de door die ambtenaar te geven aanwijzingen.

**De volgende artikelen hebben toepassing op** zoogdieren, reptielen, amfibieën en vogels.

**Artikel 1.12. besluit houders van dieren: Besparen vermijdbare vorm van pijn, spanning of lijden**

Bij het doden van dieren en daarmee verband houdende activiteiten wordt de dieren elke vermijdbare vorm van pijn, spanning of lijden bespaard.

**Artikel 1.13. besluit houders van dieren: Methoden**

1. Een dier wordt gedood door middel van een methode die waarborgt dat de dood onmiddellijk of na maar vóórdat de bewusteloosheid is geweken, intreedt.
2. In afwijking van het eerste lid behoeft een dier niet te worden bedwelmd indien een dier moet worden gedood:

    **a.** ter beëindiging of voorkoming van onmiddellijk gevaar voor mens of dier of

    **b.** ter beëindiging van ondraaglijk lijden van het dier.

3. Bij ministeriële regeling kunnen voor de daarin te onderscheiden diersoorten of categorieën dieren nadere regels worden gesteld ten aanzien van de methode, bedoeld in het eerste lid.

**Artikel 1.14. besluit houders van dieren: Kennis**

1. Het doden van dieren en daarmee verband houdende activiteiten worden uitgevoerd door personen die aantoonbaar de nodige kennis en vaardigheden bezitten om de taken humaan en doeltreffend uit te voeren.
2. Bij ministeriële regeling kunnen voor de daarin te onderscheiden diersoorten of categorieën dieren nadere regels worden gesteld ten aanzien van de in het eerste lid bedoelde kennis en vaardigheden.

***Voorbeeld:*** *Een man heeft een slang en voert deze slang dode konijnen. Hij koopt levende konijnen en maakt deze konijnen dood als ze groot genoeg zijn. Hij doet dit door de konijnen de nek om te draaien. Vervolgens legt hij hierna de konijnen in de vriezer. Er blijkt dat enkele dieren niet direct dood zijn.*

*De man is strafbaar omdat hij de dieren niet eerst heeft bedwelmd. Art 1.13 BHVD ivm 2.10 lid 3 WD. Tevens maakt hij zich schuldig aan artikel 1.12 BHVD ivm art 2.1 lid 1 WD voor de lijdende konijnen in de vriezer.*

*Dit is een misdrijf welke onder art 67 SV valt.*

## Belangrijke wetsartikelen voor de FO:

| | | |
|---|---|---|
| **Doden van dieren** (honden/ katten/ganzen) | 2.10 WD i.v.m. 8.11 lid 1 Wet Dieren ivm 1.19 Besluit houders van dieren | **M = Misdrijf/ VH** |
| **Doden van dieren** (zoogdieren, reptielen, amfibieën en vogels) | 2.10 WD lid 2 Besluit houders van dieren 1.12/1.13/1.14 ↓ | **M/VH** |
| **Pijn, angst of lijden te besparen bij doden** | 1.12 Besluit Houders van Dieren i.v.m. 2.10 lid 3 Wet Dieren i.v.m. 1 lid 1 Wet Economische Delicten i.v.m. 2 lid 1 Wet Economische Delicten | **M/VH** |
| **Doden dier zonder bedwelmen/bewusteloos** | 1.13 lid 1 Besluit Houders van Dieren i.v.m. 2.10 lid 3 Wet Dieren i.v.m. 1 lid 1 Wet Economische Delicten i.v.m. 2 lid 1 Wet Economische Delicten | **M/VH** |
| **Dier doden zonder kennis** | 1.14 lid 1 Besluit Houders van Dieren i.v.m. 2.10 lid 3 Wet Dieren i.v.m. 1 lid 1 Wet Economische Delicten i.v.m. 2 lid 1 Wet Economische Delicten | **M/VH** |
| **Dierenmishandeling** | 2.1 lid 1 WD i.v.m. 8.11 lid 1 Wet Dieren | **M/VH** |
| **Schoppen** | 1.3 onder b Besluit houders van dieren i.v.m. 2.1 lid 3 Wet Dieren i.v.m. 2.1 lid 1 Wet Dieren i.v.m. 8.11 lid 1 Wet Dieren | **M/VH** |

| | | |
|---|---|---|
| **Ontdoen dier** | 1.3 onder c Besluit houders van dieren i.v.m. 2.1 lid 3 Wet Dieren i.v.m. 2.1 lid 1 Wet Dieren i.v.m. 8.11 lid 1 Wet Dieren | **M/VH** |
| **Geslagen (letsel)** | 1.3 onder c Besluit houders van dieren i.v.m. 2.1 lid 3 Wet Dieren i.v.m. 2.1 lid 1 Wet Dieren i.v.m. 8.11 lid 1 Wet Dieren | **M/VH** |
| **Explosief. Bijtende of brandende vloeistof** | 1.3 onder d Besluit houders van dieren i.v.m. 2.1 lid 3 Wet Dieren i.v.m. 2.1 lid 1 Wet Dieren i.v.m. 8.11 lid 1 Wet Dieren | **M/VH** |
| **Onthouden van nodige zorg** | 2.2 lid 8 Wet Dieren i.v.m. 8.11 lid 1 Wet Dieren | **M/VH** |
| Vervoermiddel voortbewegen of verplaatsen op een dier (als het kracht te boven gaat) zoals bijv. ponypletters | 1.3 onder f Besluit houders van dieren i.v.m. 2.1 lid 3 Wet Dieren i.v.m. 2.1 lid 1 Wet Dieren i.v.m. 8.11 lid 1 Wet Dieren | **M** |
| Halsband met scherpe delen | 1.3 onder g Besluit houders van dieren i.v.m. 2.1 lid 3 Wet Dieren i.v.m. 2.1 lid 1 Wet Dieren i.v.m. 8.11 lid 1 Wet Dieren | **M** |
| Stroomstoten | 2.1 lid 1 Wet Dieren i.v.m. 8.11 lid 1 Wet Dieren | **M** |
| Arbeid met dier | 2.1 lid 2 onder a Wet Dieren i.v.m. 2.1 lid 1 Wet Dieren i.v.m. 8.11 lid 1 Wet Dieren | **M** |
| Koe met overvolle uiers | 2.1 lid 2 onder b Wet Dieren i.v.m. 2.1 lid 1 Wet Dieren i.v.m. 8.11 lid 1 Wet Dieren | **M** |
| Hond als trekkracht | 2.1 lid 2 onder d Wet Dieren i.v.m. 2.1 lid 1 Wet Dieren i.v.m. 8.11 lid 1 Wet Dieren | **M** |
| Onthouden zorg hulpbehoevend dier | 2.1 lid 6 Wet Dieren i.v.m. 8.11 lid 2 Wet Dieren | **O**= Overtreding |
| Lijden of letsel door beperkte vrijheid | 1.6 lid 1 Besluit houders van Dieren i.v.m. 2.2 lid 10 Wet dieren i.v.m. 8.11 lid 2 Wet Dieren | **O** |
| Ruimte voor fysiologische en ethologische behoefte | 1.6 lid 2 Besluit houders van Dieren i.v.m. 2.2 lid 10 Wet dieren i.v.m. 8.11 lid 2 Wet Dieren | **O** |
| Bescherming tegen weersomstandigheden/roofdieren | 1.6 lid 3 Besluit houders van Dieren i.v.m. 2.2 lid 10 Wet dieren i.v.m. 8.11 lid 2 Wet Dieren | **O** |
| Ontsnappen uit kooi/gebouw tegengaan | 1.6 lid 4 Besluit houders van Dieren i.v.m. 2.2 lid 10 Wet dieren i.v.m. 8.11 lid 2 Wet Dieren | **O** |
| Kennis/vaardigheden om dier te verzorgen | 1.7 onder a i.v.m. 2.2 lid 10 Wet dieren i.v.m. 8.11 lid 2 Wet Dieren | **O** |
| Dier niet onder hoede van capabel iemand | 1.7 onder b i.v.m. 2.2 lid 10 Wet dieren i.v.m. 8.11 lid 2 Wet Dieren | **O** |
| Medische zorg bij gewond dier | 1.7 onder c Besluit Houders van Dieren i.v.m. 2.2 lid 10 Wet dieren i.v.m. 8.11 lid 2 Wet Dieren | **O** |
| Hygiëne verblijf | 1.7 onder d Besluit Houders van Dieren i.v.m. 2.2 lid 10 Wet dieren i.v.m. 8.11 lid 2 Wet Dieren | **O** |
| Juiste voeding en juist toegediend | 1.7 onder e Besluit Houders van Dieren i.v.m. 2.2 lid 10 Wet dieren i.v.m. 8.11 lid 2 Wet Dieren | **O** |
| Water hoeveelheid en kwaliteit | 1.7 onder f Besluit Houders van Dieren i.v.m. 2.2 lid 10 Wet dieren i.v.m. 8.11 lid 2 Wet Dieren | **O** |
| Lucht, ventilatie, zuurstof | 1.7 onder g Besluit Houders van Dieren i.v.m. 2.2 lid 10 Wet dieren i.v.m. 8.11 lid 2 Wet Dieren | **O** |
| Verlichting/belichting | 1.8 lid 1 Besluit Houders van Dieren i.v.m. 2.2 lid 10 Wet dieren i.v.m. 8.11 lid 2 Wet Dieren | **O** |
| Ruimte met scherpe/uitstekende delen | 1.8 lid 2 Besluit Houders van Dieren i.v.m. 2.2 lid 10 Wet dieren i.v.m. 8.11 lid 2 Wet Dieren | **O** |
| Bodembedekking | 1.8 lid 3 Besluit Houders van Dieren i.v.m. 2.2 lid 10 Wet dieren i.v.m. 8.11 lid 2 Wet Dieren | **O** |

| | | |
|---|---|---|
| Eenvoudig kunnen ontsmetten | 1.8 lid 4 Besluit Houders van Dieren i.v.m. 2.2 lid 10 Wet dieren i.v.m. 8.11 lid 2 Wet Dieren | **O** |
| Ketting en ingesloten | 3.1 lid 1 onder a besluit houders van dieren i.v.m. 2.2 lid 10 Wet dieren i.v.m. 8.11 lid 2 Wet Dieren | **O** |
| Eisen vastleggen | 3.2 lid (1 tm 4 mogelijk) besluit houders van dieren i.v.m. 2.2 lid 10 Wet dieren i.v.m. 8.11 lid 2 Wet Dieren | **O** |
| Eisen ren | 3.3 lid (1 tm 4 mogelijk) besluit houders van dieren i.v.m. 2.2 lid 10 Wet dieren i.v.m. 8.11 lid 2 Wet Dieren | **O** |

Gezondheid ouders of pups benadelen door fokken **M/O**
(bv erfelijke afwijkingen)
3.4 lid (1 of 2) besluit houders van dieren i.v.m. 2.6 lid 2 Wet Dieren i.v.m.
- 1 onderdeel 2 Wet Economische Delicten i.v.m. 2 lid 1 Wet Economische Delicten (overtreding, bij opzet een misdrijf) voor wat betreft de onderwerpen 2.6 lid 2 onderdeel a onder 2 en 3 **(fokverbod met dieren die gevaar kunnen opleveren voor mens of dier)**
- artikel 1 onderdeel 4 (overtreding) voor wat betreft de onderwerpen 2.6 lid 2 onderdeel a onder 1, b,c,d,e en f en het derde lid **(fokverbod voor dieren die beschikken over een bepaalde aandoening die, of een uiterlijk kenmerk dat, de gezondheid of welzijn van het dier of de nakomelingen van het dier kan aantasten; methode van fokken, het voorafgaand te verrichten onderzoek, het aantal nesten dat dieren gedurende een bepaalde periode mag krijgen. )**

| | | |
|---|---|---|
| Gezondheid benadelen door fokken (aantal nesten) | 3.4 lid 2 onder e Besluit Houders van Dieren i.v.m. 2.6 lid 2 Wet Dieren i.v.m. 1 onderdeel 2 Wet Economische Delicten i.v.m. 2 lid 1 Wet Economische Delicten | **M** |
| Meer dan 1 nest binnen 12 maanden voor hond | 3.4 lid 3 onder e Besluit Houders van Dieren i.v.m. 2.6 lid 2 Wet Dieren i.v.m. 1 onderdeel 2 Wet Economische Delicten i.v.m. 2 lid 1 Wet Economische Delicten | **M** |
| Meer dan 2 nesten in 12 maanden of 3 nesten in 24 maanden, kat | 3.4 lid 4 onder e Besluit Houders van Dieren i.v.m. 2.6 lid 2 Wet Dieren i.v.m. 1 onderdeel 2 Wet Economische Delicten i.v.m. 2 lid 1 Wet Economische Delicten | **M** |
| Scheiden pup van ouder (hond/kat 7 weken) | 1.20 lid 1 Besluit Houders van Dieren i.v.m. 1.19 Besluit Houders van Dieren i.v.m. 2.2 lid 7 Wet Dieren i.v.m. 1 onder 4 Wet Economische Delicten i.v.m. 2 lid 4 Wet Economische Delicten | **O** |
| Levend aas | 1.18 Besluit Houders van Dieren i.v.m. 2.3 lid 3 Wet Dieren i.v.m 8.11 lid 2 Wet Dieren | **O** |
| Dier als prijs/beloning | 2.13 Wet Dieren i.v.m. 1 onder 4 Wet Economische Delicten i.v.m. 2 lid 4 Wet Economische Delicten | **O** |
| Dierengevecht organiseren | 2.14 lid1 Wet Dieren i.v.m 8.11 lid 1 WD | **M/VH** |
| Dierengevecht bijwonen | 2.14 lid2 Wet Dieren i.v.m 8.11 lid 2 Wet Dieren | **O** |
| Dier met verboden ingreep in de verkoop | 2.7 lid3 Wet Dieren i.v.m. 1 onder 4 Wet Economische Delicten i.v.m. 2 lid 4 Wet Economische Delicten | **O** |
| Dier met verboden ingreep tentoonstellen | 2.16 lid 3 Wet Dieren i.v.m. 1 onder 4 Wet Economische Delicten i.v.m. 2 lid 1 Wet Economische Delicten | **O** |
| Aanbieden dood dier | 3.23 Regeling Dierlijke Producten i.v.m. 3.4 lid 3 Wet Dieren i.v.m. 1a onder 3 Wet Economische Delicten i.v.m. 2 lid 3 Wet Economische Delicten | **O** |
| Dier niet chippen | 6 Besluit identificatie en registratie i.v.m. 96 Gezondheid en Welzijnswet Dieren i.v.m. 1 onder 2 WED i.v.m. 2 lid 1 WED | **O** |

In WED (wet economische delicten) is opzettelijk een misdrijf en schuld een overtreding!!

# BESTIALITEIT seks met dieren (Zoöseksualiteit)

**Bestialiteit:** is een term die seksuele betrekkingen met dieren beschrijft vaak uitgevoerd door mensen met zoöfilische voorkeur.

**Zoöfilie:** is de seksuele voorkeur van mensen die zich erotisch aangetrokken voelen tot dieren. Een vorm van zoöfilie, is dat een mens een relatie aangaat met een dier zoals een mens tot mens relatie.

**Parafilie:** is de verzamelnaam van een groep seksuele gedragingen of fantasieën die over het algemeen als afwijkend van de heersende normen worden beschouwd. Een voorbeeld hiervan is een fetisj en een parafilie waarbij seksuele opwinding wordt geassocieerd met het observeren dat objecten (dieren) worden verpletterd. **Animal crush** is een kwelspel waarbij men ter vermaak, dieren leed aandoet. Bij kwelspelen worden dieren geestelijk of lichamelijk mishandeld, soms tot de dood erop volgt. Animal crush kan men als een moderne variant van het kwelspel zien, waarin men ter vermaak, soms met een seksuele motivatie, kleinere dieren met de (blote) voeten vertrapt. Er zijn crushfilms in de omloop. Een Nederlands voorbeeld van deze vorm is de "ponypletter". In deze zaak werden video's aangetroffen van pony's, paarden, ezels, geiten, varkens, honden, katten, konijnen en een os die werden onderworpen aan handelingen die binnen animal crush vallen.

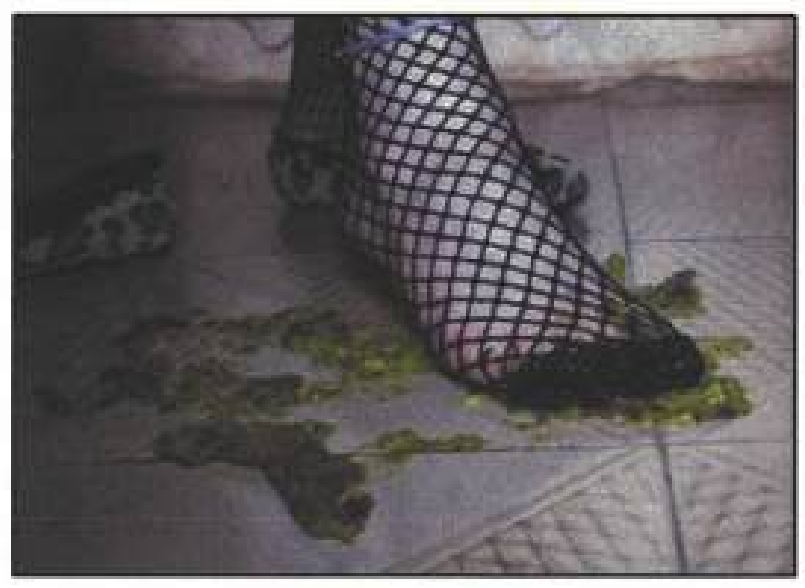

De meeste daders van ontuchtige handelingen met dieren hebben alle aspecten in seks al doorlopen waardoor niets meer spannend is.

Profiel van daders:

- Meer mannen en enkele vrouwen
- Beperkte agressiebeheersing
- Weinig vaardigheden in probleem oplossen
- Moeite met communicatie in de relatie, moeite zich te uiten
- Behoefte de controle te hebben.

In geval van ontucht met dieren zal de Forensische Opsporing worden ingeschakeld. In sommige Politie eenheden zal ook Team zeden aansluiten. Er zal een Forensisch onderzoek plaatsvinden. Onderzoek kan plaatsvinden aan en in het lichaam van het dier. Ook kan er een sporenonderzoek op de locatie plaatsvinden, waar het incident zich heeft afgespeeld. (PD onderzoek).

- **Ontucht met een dier (art 254 Sr, geen VH feit) is veelal ook Dierenmishandeling (art. 2.1 Wet dieren en art. 1.3 Besluit houders van dieren, wel VH feit en genoemd in artikel 67 WvSV)**
- **Pornografie met dieren (254a Sr, genoemd in artikel 67 onder b WvSV)**

Laat inwendige bemonsteringen uitvoeren door een (forensische) dierenarts en stel de sporen veilig. (In principe mogen wij dit ook, er is niet bij wet geregeld welke bevoegdheden een dierenarts heeft. Echter ontbreekt onze kennis hoe diep de vaginale bemonstering zou moeten, waardoor het inschakelen van een dierenarts verstandiger is).
Een cotton swab mag voor de (vaginale) bemonstering gebruikt worden, maar natuurlijk ook de Nylon flocked swabs die worden gebruikt tijdens het Forensisch Medisch Onderzoek (FMO). Neem deze swabs zelf mee naar de locatie waar het dier wordt bemonsterd door een dierenarts. Het gebruiken van een zedenset is ook mogelijk. (Indien er kan volstaan met een enkele swab, gelieve dan losse swabs te gebruiken vanwege de kosten)

Onderzoek eventueel de plaats waar het zedendelict heeft plaatsgevonden. Heeft een verdachte op een ton gestaan (schoensporen) of zijn er nog attributen gebruikt waarvan DNA kan worden veiliggesteld. Denk ook aan digitale sporen zoals locatievoorziening, foto's en filmpjes die kunnen zijn gemaakt.

Vaak kan met de eigenaar van het dier, mits geen verdachte, een afspraak worden gemaakt voor het sporenonderzoek aan het dier en zal hij/zij willen meewerken aan dit onderzoek. Als het echter nodig is om een levend dier in beslag te nemen voor onderzoek, overleg dan met de officier van Justitie en volg verder de procedure van beslagname van een dier. De opslag gaat via RVO.nl. (Zie netwerkkaart). De collega met taakaccent DCG, zal dit in principe afhandelen.

Sporen kunnen hierna worden ingezonden naar het Nederlands Forensisch Instituut, uiteraard na prioritering. Er hoeft geen daderindicatie te zijn.
Voor humane dadersporen is dit regulier HBS, voor dierlijk materiaal is dit NHBS. HBS 004 en 007 kunnen door de officier van Justitie in het prioritering overleg worden aangedragen ivm de SLA-capaciteit. Voor NHBS is ook een benoeming nodig. Deze hoeven niet via het prioritering overleg.

# Hondengevechten/Diergevechten

In de wet zijn gevechten met dieren verboden. Dit valt onder Verbod op dierengevechten
<u>Artikel 2.14 wet dieren</u>
1. Het is verboden dierengevechten te organiseren of dieren aan dierengevechten te doen deelnemen.
2. Het is verboden bij dierengevechten aanwezig te zijn

Strafbaarstelling: Artikel 8.11 lid 1 WD voor wat betreft art 2.14 lid 1 → in art 8.12 lid 1 WD: 3 jaar hechtenis is VH-feit, genoemd in art 67 WvSV.
Artikel 8.11 lid 2 WD voor wat betreft art 2.14 lid 2 → in art 8.12 lid 3 WD: 6 maanden hechtenis.

Hondengevechten vinden plaats in woningen of in leegstaande panden. De radio wordt hard aangezet, de gordijnen gesloten en niemand heeft in de gaten wat er zich binnen afspeelt. Voor hondengevechten worden de zogenaamde hoog-risico honden gebruikt, met name de Pitbull- of Stafford- achtige typen honden. De regels zijn strikt. Zo moet de pit (zie uitleg hieronder) aan bepaalde eisen voldoen.
Het doel van de gevechten is een status te verwerven en er wordt heel veel geld verdiend met het illegaal gokken. Er wordt met veel geld gewed, wat vervolgens wordt geïnvesteerd in criminele activiteiten. Onderzoek leert dat hondengevechten een korte lijn hebben met drugscriminaliteit.
Honden die moeten vechten worden eerst getest en getraind. Een hond moet gedragskenmerken vertonen van "niet te stoppen" en volhardend in het afmaken van de taak (gameness). Deze honden gaan vaak door met gebroken en verminkte ledematen. Het trainen wordt gedaan met levende dieren zoals katten, konijnen of oefenhonden. De oefenhonden worden gestolen of opgehaald uit een asiel of zijn door de hondenvechters zelf gefokt. Honden die verliezen worden vaak verhangen, geëlektrocuteerd of verdronken. Honden die de status van Champions hebben verkregen en puppy's uit die bloedlijn, zijn vaak duizenden euro's waard.

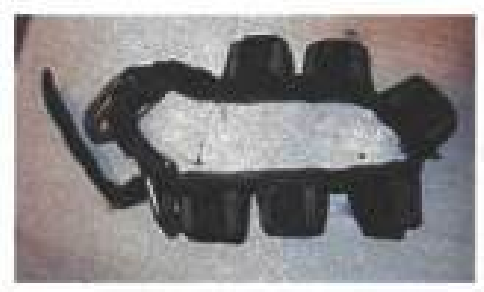

Wat kunnen we aantreffen in een woning waar hondengevechten plaatsvinden?
- Bloed op de grond/muur/vensterbank
- Break stick
- Verzwaarde halsbanden met lood
- Anabolen steroïden
- Medicatie zoals antibiotica, hechtmateriaal (is verboden en voorbehouden aan dierenarts)
- Nesten puppy's
- Attributen om de kaakkracht te trainen, zoals een autoband in de boom waar ze zich aan vast moeten bijten.
- Loopbanden/ spring pole/ treadmill

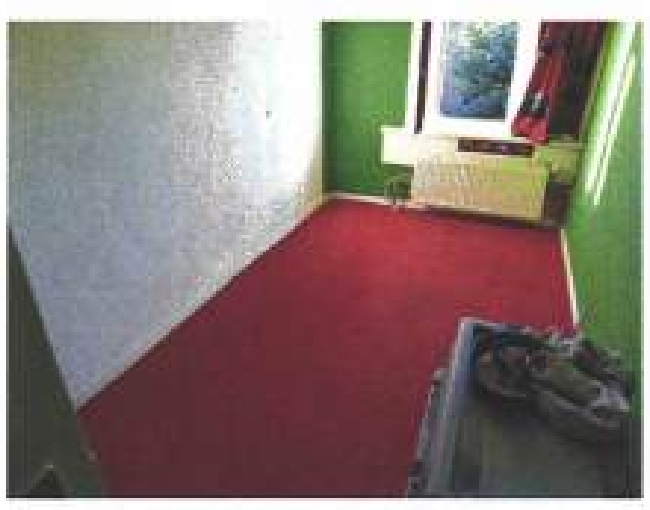
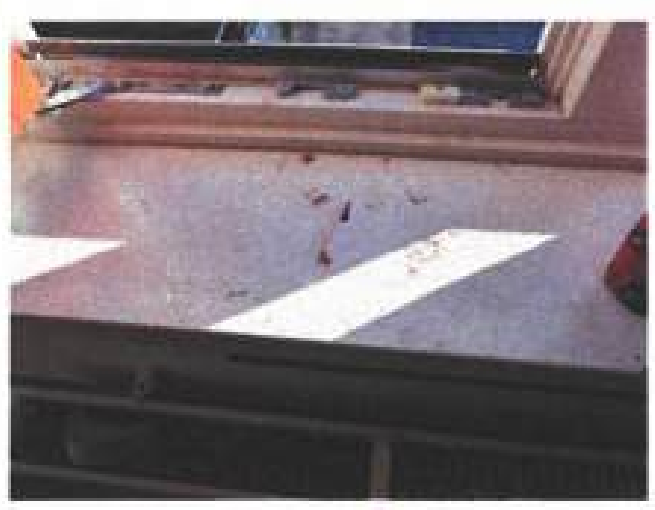
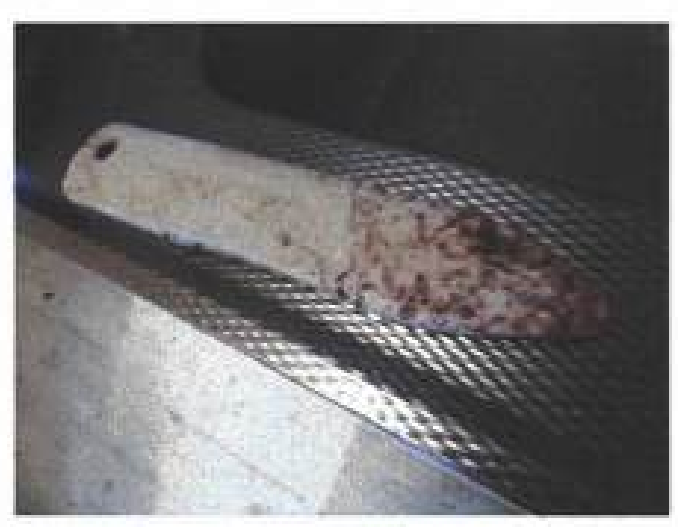

**Pit**: een piste/ring waarin gevechten worden gehouden; een doorsnee pit is gemaakt van wanden van triplexplaten met een hoogte van 61 cm tot 91 cm (24 tot 36 inch) en een oppervlakte van ongeveer 4,5 tot 6 vierkante meter (14 tot 20 vierkante feet), hoewel een pit ook wel wordt gemaakt van beton, metalen platen en hooibalen; de vloer van een pit is normaal gesproken bedekt met een vloerkleed of zeildoek zodat een hond gemakkelijker voort-/meegetrokken kan worden; veel pits zijn te demonteren en derhalve verplaatsbaar

**Break stick (breaking stick, bite stick)**: een wigvormige stok die wordt gebruikt om de kaakgreep van een pit bull te (ver)breken
**Spring pole**: een toestel dat wordt gebruikt om een pit bull te trainen/te laten bewegen
**Treadmill:** een toestel dat wordt gebruikt om de hond op de plaats te laten rennen
**Match**: georganiseerd hondengevecht
**Champion**: hond die drie matches heeft gewonnen
**Grand Champion**: hond die vijf matches heft gewonnen
**Gameness:** De wil van de hond te vechten en door te gaan tot het einde

Hondenvechters zien hondengevechten niet als dierenmishandeling maar als sport. Hondenvechters zeggen de dieren juist te gebruiken waar ze van nature voor zijn geprogrammeerd; vechten. Ze vinden dat ze goed voor hun dier zorgen en dat de honden alle aandacht, beweging en voeding krijgen die ze nodig hebben. De hond heeft er naar hun zeggen plezier in om te vechten en omdat de interactie met de "baas" groot is, zijn ze samen "iets leuks" aan het doen.

**Weightpulling**
Omdat hondengevechten zijn verboden is de hondensport Weightpulling ontstaan. Weightpulling kan worden gezien als een krachtmeting tussen twee honden en is in Nederland eveneens verboden. In de Wet Dieren staat dat een hond niet mag worden ingezet om op het land of in het water een kar, slee, boot of ander voorwerp, mens of dier voort te trekken.
Voor sledehondensport is hiervoor een uitzondering gemaakt. Er een ontheffing worden aangevraagd voor raszuivere hondenrassen (met stamboom) zoals een husky, groenlandse hond, eskimohond, Malamute en Samojeed. Voor alle andere rassen wordt GEEN ontheffing verleend!

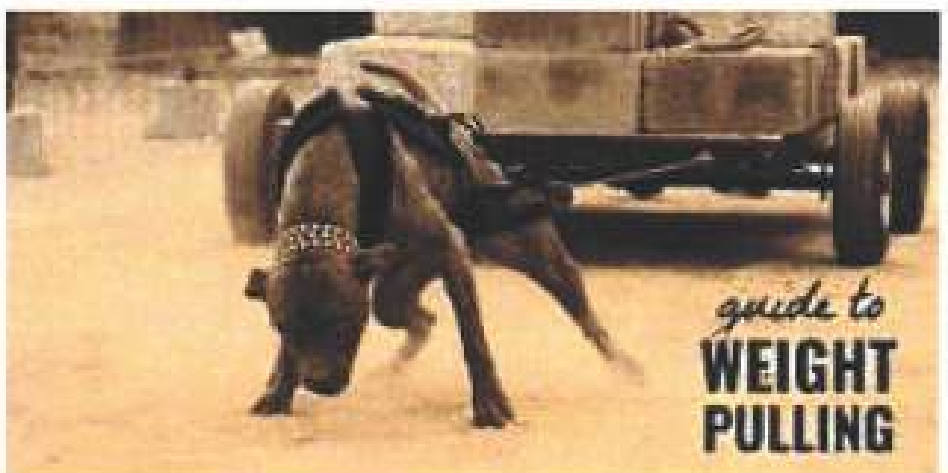


Bron: "Hoog-risico honden een bijtend probleem" en Carin Ottburg-van Dellen tactisch coördinator Politie-Eenheid midden Nederland.

**Hanengevechten**
Bij hanengevechten worden meestal twee (meerdere kan ook) hanen tegenover elkaar gezet in een afgezette ring. De beesten zien de ring als hun territorium en ze dulden daarin geen tegenstander. Ze dagen elkaar uit en vechten vaak tot de dood erop volgt. Hanen dulden geen rivalen in hun territorium en gaan gevechten aan om het te beschermen. De haan zet de veren in zijn nek recht, pikt de indringer en daagt hem uit. Bij een duel proberen de hanen elkaars kam uit te pikken en elkaar te kwetsen met hun sporen.

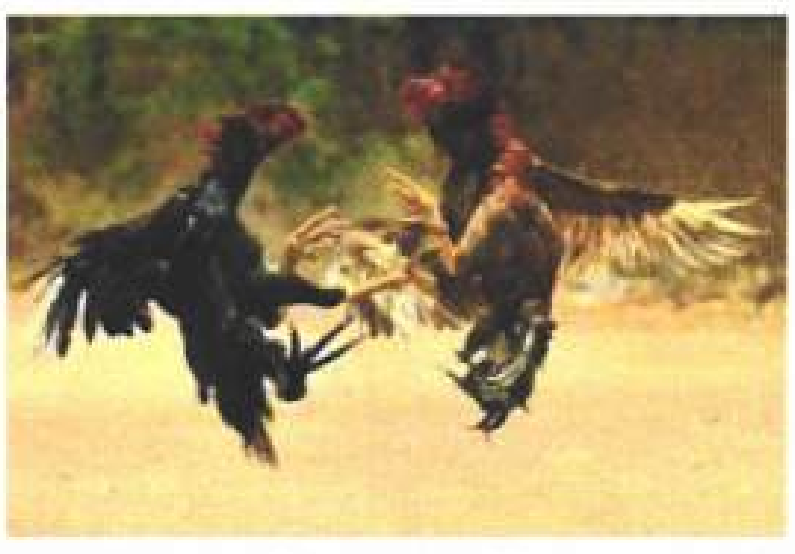

De hanen worden vaak opgejut door mensen die om de ring heen staan. Die mensen gokken (in Nederland illegaal) op de wedstrijden, of zien de vechtende beesten puur als vermaak.
Omdat er geld met gokken te verdienen is, worden hanen speciaal gefokt voor de wedstrijden. Gevechten tussen de dieren kunnen heel lang duren. Om wat meer snelheid in het gevecht te brengen, worden er soms mesjes aan de poten van de hanen bevestigd. Dan gaat de andere haan sneller dood.
Ook hanengevechten zijn in Nederland bij wet verboden. Het bijwonen van dierengevechten is eveneens strafbaar.

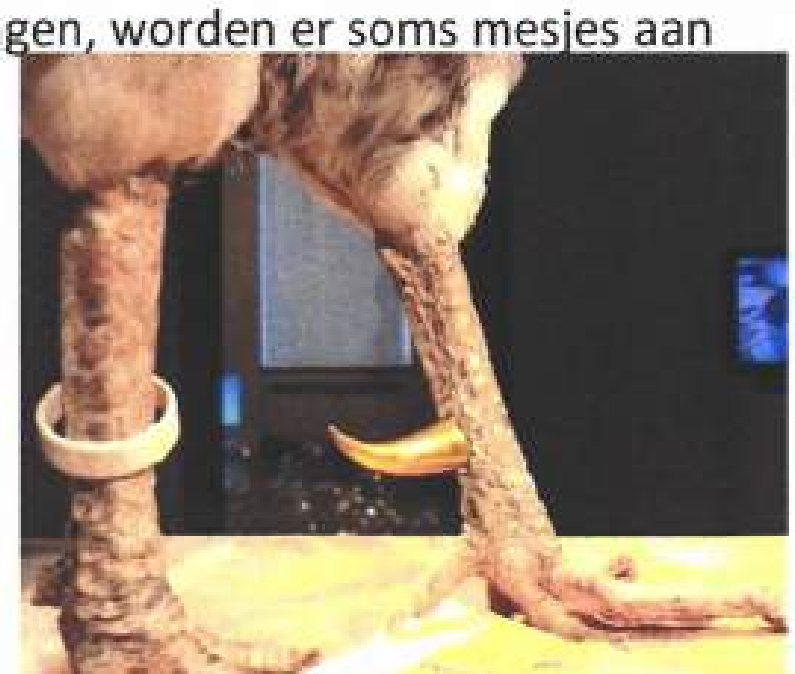

# Signalenkaart politie

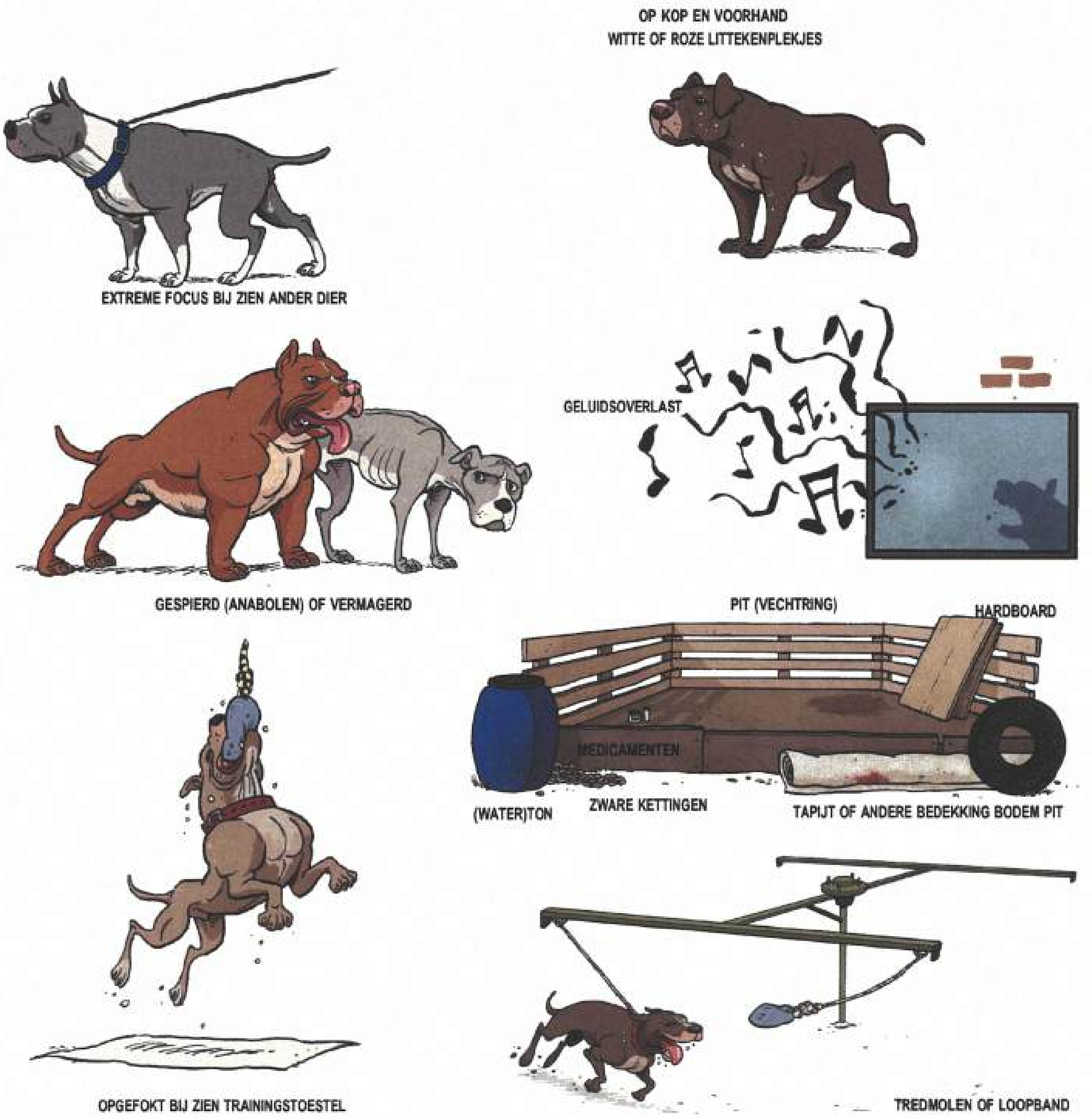


**BELANGRIJKE SIGNALEN**

Littekens en kale plekjes op kop/voorpoten

Verwondingen in verschillende stadia van helen of slecht verzorgd

Sommige dieren mogelijk zeer gespierd, andere juist mager

Honden mogelijk op verschillende adressen ondergebracht

Aanwezigheid van tredmolens, hardboard, anabole steroïden, underlayment, kleden voor de vechtring, al dan niet bebloed

Adres mogelijk al bekend i.v.m. geluidsoverlast (blaffen) of bijtincidenten

# Signalen dier, eigenaar en leefomgeving

Bij levende dieren die stelselmatig worden mishandeld zal een gedragsverandering plaatsvinden. Vooral na bestialiteit is er vaak afwijkend gedrag waarneembaar. Deze constatering, indien onderbouwd, mag in het proces-verbaal worden vermeld.

## Lichamelijke conditie dier

- **In het oog springende afwijkingen, verwondingen, kneuzingen (vaak slecht zichtbaar door vacht)**
- **Heeft (mogelijk door een mens) toegebrachte verwondingen of onverklaarbaar letsel**
- **Breuken en/of wonden in verschillende fasen van genezing**
- **Moeite met staan, bewegen, zitten of liggen**
- Mager voorkomen of juist overgewicht
- Slechte algehele lichamelijke toestand; bekijk goed de vacht, nagels, indien mogelijk het gebit
- Bekijk de ogen, neus en bek van het dier. Zijn deze schoon zonder uitvloeiing, staan ogen helder
- Parasitaire (vlooien, wormen, luizen) en respiratoire aandoeningen (luchtweg problemen)

## Gedrag dier

- **Angstig** (voor eigen baasje), schrikachtig
- Dier zoekt steun bij onbekende, bovenmatig sociaal.
- **Pijn vermijdingsreflex** (Het dier trekt zichzelf of een lichaamsdeel terug bij aanraking)
- Vertoont **stress signalen**; hijgen, vergrote pupillen, oogwit is te zien/wijd opengesperde ogen, trillen, bevriezen
- Vocaliseren; blaffen, schreeuwen, janken, piepen, grommen
- Automutilatie, herhaalde beweging/zelfbeschadiging/zonder fysieke reden continue krabben, kopschudden/neurotisch
- Overdreven **agressief of timide**
- Heeft een afgestompte, depressieve houding
- Plotselinge verandering in gedrag of gedragsverandering t.o.v. voorheen
- Vermijdt contact, vertoont vluchtgedrag

## Signalen in de leefomgeving

- Geluidoverlast (van de bewoners of het dier)
- Onhygiënische leefomgeving
- Weinig beweegruimte, te weinig lichaamsbeweging of uitdaging
- Dier verblijft de hele dag opgesloten (de omgeving weet bijvoorbeeld niet dat een hond in huis is)
- Dier verblijft buiten tijdens extreme weersomstandigheden en/ of het ontbreekt aan een eigen ligplaats of hok
- Geen voer of waterbak
- Wel voer en waterbak maar zeer vies, zonder inhoud of buiten bereik van het dier
- Veel nakomelingen en/of veel (niet gecastreerde) dieren
- Bij hoge aantallen dieren; inteelt, kannibalisme, infectieziekten onder de dieren,
- Feces, urine, braaksel, bloed zichtbaar in woning
- Hoge ammoniak waarden door urine
- Hoarding (verzamelen)

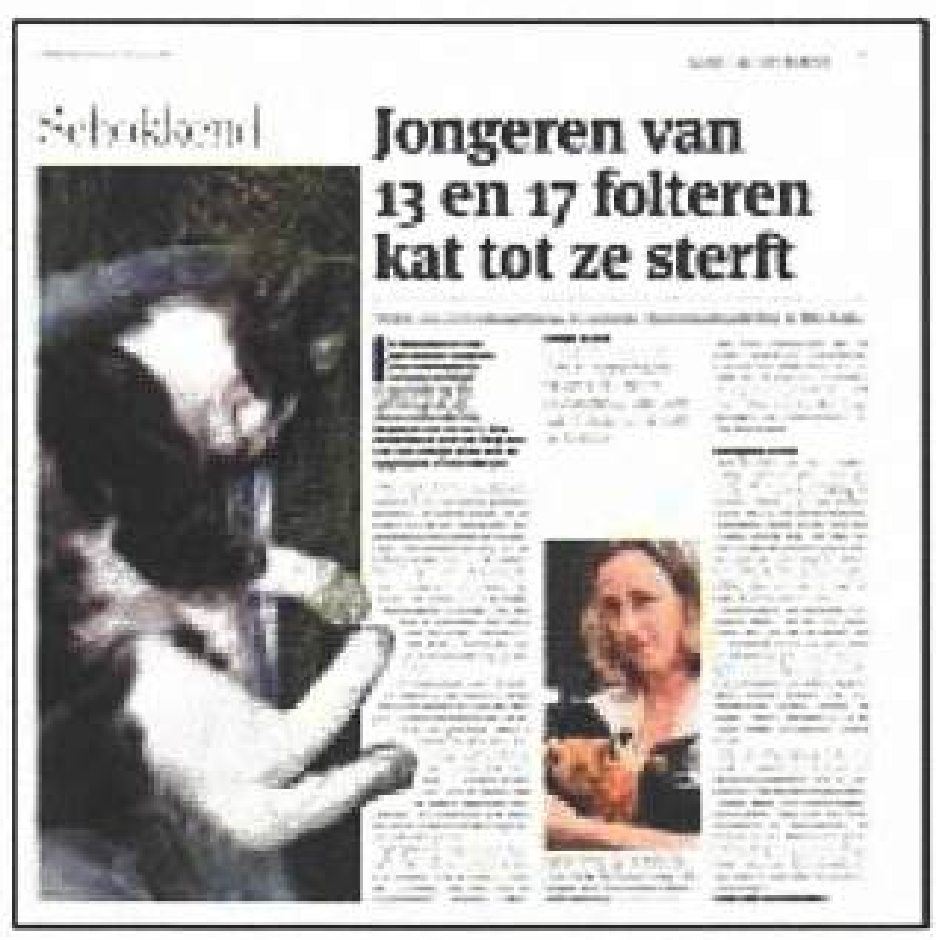
Jongeren van 13 en 17 folteren kat tot ze sterft

## Gedrag eigenaar ten opzichte van het dier

- Onjuiste omgang met dier (bijv. ruw, gehaast, onverschillig, schreeuwen, sadistisch, kleineren)
- Ziet geweld als een middel om (gedrag)problemen op te lossen (denk ook aan gebruik van middelen zoals prik- of stroombanden)
- Heeft irreële verwachtingen van het dier
- Onthouding van nodige basisbehoeften zoals voeding, water, sociale interactie en medische zorg

- Te weinig uitlaten van de hond (minimaal 3-4 x per dag)
- Vertoont geen tot minimale interactie met het dier of vertoont juist overdreven interactie
- Terughoudend in het geven van een verklaring voor letsel bij het dier of de verwaarloosde staat
- Geeft onwaarschijnlijke of verschillende verklaringen voor hetzelfde letsel
- Gezinsleden geven tegenstrijdige verklaringen voor de verwaarloosde staat of het letsel
- Vertelt een onsamenhangend verhaal dat niet passend is bij de situatie
- Reageert ongepast, boos of agressief op vragen of op berichtgeving
- Gebrek aan interesse in - of bezorgdheid om het dier
- Vertoont normafwijkend gedrag: is onverschillig, angstig, nerveus, agressief of ongemakkelijk
- Weigert aangeboden hulp aan te nemen of te zoeken; hulp vermijdend
- Wisselt vaak van dierenkliniek (shopper)
- Wijst anderen als schuldige aan
- Heeft meerdere dieren en toont interesse naar nog meer dieren
- Heeft vaak nieuwe dieren en de oude dieren zijn verdwenen
- Heeft zelf geen overzicht of reëel beeld van de situatie

Algemene verzorgingsnormen van huisdieren https://www.licg.nl/

Deze **signalenkaart** is tot stand gekomen i.s.m.Stichting Mendoo. Deze Stichting draagt bij aan het delen van kennis en het creëren van bewustwording over de **link** tussen **huiselijk geweld en dierenmishandeling**. tel. 06-40261435 info@mendoo.nl www.mendoo.nl

**Wetenschappelijke achtergrond**
De samenhang tussen huiselijk geweld en dierenmishandeling is uitgebreid beschreven in Nederlandstalige onderzoeken door Enders-Slegers en Janssen (2009), Garnier en Enders-Slegers (2012) en Langeveld en Dreunhuizen (2019, & Ascione, 2018; Levitt, Hoffer, & Loper, 2016). Tussen de 24% en 79% van de ondervraagde vrouwelijke slachtoffers van huiselijk geweld (veelal in opvangcentra) rapporteert een samenloop van partnergeweld en dierenmishandeling. Plegers kunnen gemotiveerd zijn huisdieren te mishandelen om hun partner te controleren, dwingen, bedreigen en intimideren (Simmons & Lehmann, 2007). Het dreigen met dierenmishandeling wordt soms ingezet om kinderen stil te houden die het slachtoffer zijn van seksueel misbruik. Slachtoffers van huiselijk geweld met huisdieren rapporteren dat zij hun vlucht uitstellen door gebrek aan geschikte opvang voor het huisdier. Kinderen die zelf mishandeld zijn geweest (McEwen, Moffitt & Arseneault, 2014) of getuige zijn geweest van huiselijk geweld of dierenmishandeling (Ascione, Friedrich, Heath & Hayashi, 2003) lopen een groter risico om zelf dieren te mishandelen.
