# Supplementary material for: Five years of veterinary forensic necropsies in the Netherlands: a retrospective analysis of non-human cases
Source: Int J Legal Med. 2026 Jun 3;140(5):2877–901. doi: 10.1007/s00414-026-03840-y (PMC13499755; doi:10.1007/s00414-026-03840-y)
Supplement: Supplementary file 2 [file 414_2026_3840_MOESM1_ESM.docx]

***Legal framework***

The Netherlands has a well-established legal framework governing animal welfare, primarily codified in the Wet Dieren (Animal Law), which came into effect in 2013.^[1]^ ^[2]^ This law emphasizes the intrinsic value of animals and prohibits both intentional and negligent harm to animals while imposing positive welfare obligations, ensuring species-appropriate care.^1^ Companion animals must receive adequate food, shelter, and veterinary care, while stricter regulations govern commercially kept animals.^1 2^

Criminal offenses related to animal abuse and neglect fall under the Dutch Penal Code.^2^ In the Netherlands, several specific laws guide the punishment of animal cruelty, which is conceptually grounded in the internationally recognized Five Freedoms - freedom from hunger and thirst; discomfort; pain, injury or disease; fear and distress; and the freedom to express normal behavior.^[3]^ Article 2.10, Section 1, Wet Dieren makes it illegal to kill certain animals, with exceptions for the production of animal products. Article 350, Section 2, Wetboek van Strafrecht addresses the unlawful killing or mistreatment of another person's animal. Article 2.1, Sections 1 and 6, Wet Dieren prohibits causing pain, injury, or harm to an animal without a reasonable purpose or exceeding what is necessary to achieve such a purpose. Lastly, Article 254, Wetboek van Strafrecht criminalizes indecent acts with animals. Amendments in recent years have sought to strengthen these protections, reflecting a broader societal recognition of animal welfare as an ethical and legal imperative. Recent legal amendments have significantly increased penalties for animal cruelty. As of January 1, 2024, the maximum prison sentence for severe cases of animal abuse has been raised from three to five years, and fines can now reach up to € 90,000. Additionally, courts have expanded authority to impose lifelong bans on animal ownership for repeat offenders.^[4]^ The stricter enforcement of animal welfare laws and the increase in legal penalties reflect a growing recognition of animal cruelty as a serious criminal offense, closely linked to broader patterns of violence and public safety concerns.

Together, these laws demonstrate the Netherlands' comprehensive approach to protecting animal welfare, with increasingly severe penalties for those who engage in animal cruelty.

1. D. Brouwer, K. Coulter, A. Fitzgerald, Animal Protection Organizations and Public Policy The Case of the Netherlands, Polit. Anim. 8 (2022) 1–16. www.politicsandanimals.org.
2. wetten.nl - Regeling - Wet dieren - BWBR0030250, (n.d.). https://wetten.overheid.nl/BWBR0030250/2025-01-01/?utm_source=chatgpt.com (accessed March 12, 2025).
3. A.M. Voogt, W.W. Ursinus, D.T.H.M. Sijm, J.H. Bongers, From the Five Freedoms to a more holistic perspective on animal welfare in the Dutch Animals Act, Front. Anim. Sci. 4 (2023) 1026224. https://doi.org/10.3389/FANIM.2023.1026224/BIBTEX.
4. New legislation as of 1 January 2024 | News item | Government.nl, (n.d.). https://www.government.nl/ministries/ministry-of-justice-and-security/news/2023/12/18/new-legislation-as-of-1-january-2024?utm_source=chatgpt.com (accessed March 12, 2025).
