# Supplementary material for: Five years of veterinary forensic necropsies in the Netherlands: a retrospective analysis of non-human cases
Source: Int J Legal Med. 2026 Jun 3;140(5):2877–901. doi: 10.1007/s00414-026-03840-y (PMC13499755; doi:10.1007/s00414-026-03840-y)
Supplement: Supplementary file 3 [file 414_2026_3840_MOESM2_ESM.docx]

**Figure A.** A pie chart visualizing the distribution of agencies submitting cases between 2020 and 2024 (*N* = 346), categorized in the following categories: police and governmental agencies (orange), unknown (dark orange), private (red), animal ambulance / animal welfare organization (pink), and veterinarian (blue).

**Figure B.** A bar chart representing the distribution of decomposition stages of the submitted animals at the time of forensic examination (*N* = 346). Percentages are given right to the bars.

**Figure** **C.** Distribution of presumptive manner of death in submitted forensic cases (*N* = 346).

**Figure D.** Distribution of presumptive cause of death among cases classified as non-natural, with blunt force trauma and neglect/deprivation representing the most common causes (*N* = 231).

**Figure E.**  A pie chart of the distribution of animals across submitted cases with presumptive a non-natural cause of death (*N* = 231), categorized in the categories dogs (orange), cats (yellow), birds (red), livestock (blue), rodents (pink) and wild animals (green).
