# Supplementary material for: Five years of veterinary forensic necropsies in the Netherlands: a retrospective analysis of non-human cases
Source: Int J Legal Med. 2026 Jun 3;140(5):2877–901. doi: 10.1007/s00414-026-03840-y (PMC13499755; doi:10.1007/s00414-026-03840-y)
Supplement: Supplementary file 4 [file 414_2026_3840_MOESM3_ESM.docx]

**Decomposition stages**

In the fresh/no decomposition stage, the body showed no signs of decomposition and maintained its normal color and texture. There was no odor, and the body’s temperature began to drop, with rigor mortis and livor mortis developing.

In the beginning/early decomposition stage, visible signs included bloating due to gas formation, skin discoloration (greenish-blue), and the first signs of insect activity, such as fly eggs. The body began to emit a faint odor of decay.

During moderate decomposition, the body experienced severe swelling, skin slippage, and fluid loss from natural openings. An intense odor was present, and there was heavy insect activity, with maggots visible in cavities and wounds.

In advanced decomposition, substantial tissue loss was evident, along with peak insect activity. Maggot activity caused deep tissue damage, and the body showed visible deformation due to gas loss. Adipocere or desiccation also occurred in some cases.

The severe decomposition/partial skeletonization stage was marked by the majority of the flesh being gone, leaving bones visible. Some soft tissues remained, and insect activity decreased, with beetles replacing flies.

In the mummified/dry remains stage, decomposition had halted due to desiccation, leaving leathery or dried tissue. The skin became dry, with minimal odor, and no active insects were present.

Finally, in the skeletal remains/very advanced decomposition stage, only bones remained, with no soft tissue. The bones showed signs of discoloration or encrustation due to environmental factors.

This classification system was based on observable, visible signs of decomposition, providing a systematic method to categorize the postmortem changes in the submitted animals.
